# Get Spliced: Uniting Alternative Splicing and Arthritis

**DOI:** 10.3390/ijms25158123

**Published:** 2024-07-25

**Authors:** Maurice J. H. van Haaren, Levina Bertina Steller, Sebastiaan J. Vastert, Jorg J. A. Calis, Jorg van Loosdregt

**Affiliations:** 1 Center for Translational Immunology, University Medical Center Utrecht, 3584 CX Utrecht, The Netherlands; 2 Division of Pediatric Rheumatology and Immunology, Wilhelmina Children’s Hospital, 3584 CX Utrecht, The Netherlands

**Keywords:** alternative splicing, arthritis, immunology

## Abstract

Immune responses demand the rapid and precise regulation of gene protein expression. Splicing is a crucial step in this process; ~95% of protein-coding gene transcripts are spliced during mRNA maturation. Alternative splicing allows for distinct functional regulation, as it can affect transcript degradation and can lead to alternative functional protein isoforms. There is increasing evidence that splicing can directly regulate immune responses. For several genes, immune cells display dramatic changes in isoform-level transcript expression patterns upon activation. Recent advances in long-read RNA sequencing assays have enabled an unbiased and complete description of transcript isoform expression patterns. With an increasing amount of cell types and conditions that have been analyzed with such assays, thousands of novel transcript isoforms have been identified. Alternative splicing has been associated with autoimmune diseases, including arthritis. Here, GWASs revealed that SNPs associated with arthritis are enriched in splice sites. In this review, we will discuss how alternative splicing is involved in immune responses and how the dysregulation of alternative splicing can contribute to arthritis pathogenesis. In addition, we will discuss the therapeutic potential of modulating alternative splicing, which includes examples of spliceform-based biomarkers for disease severity or disease subtype, splicing manipulation using antisense oligonucleotides, and the targeting of specific immune-related spliceforms using antibodies.

## 1. Understanding Alternative Splicing: Principles, Regulation, and Outcomes

**Figure 1:**
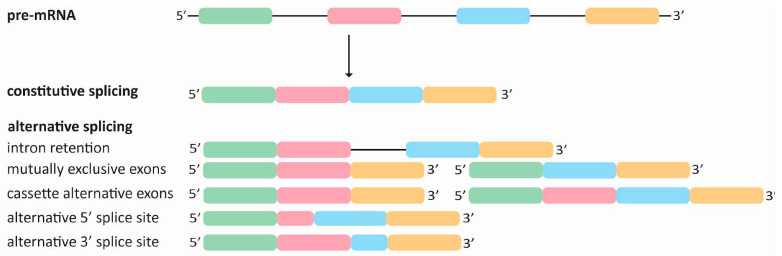
Schematic overview of splicing outcome effects on mRNA. Schematic depiction of a pre-mRNA containing four exons (colored boxes) and three introns (black lines). Constitutive splicing results in a mature transcript that contains all four exons. Different types of alternative splicing can have different outcomes. Intron retention results in the inclusion of one or more introns in the mRNA. With mutually exclusive exons, either one exon or another exon is incorporated but not both exons simultaneously. Cassette alternative exons can either be skipped or incorporated into the mature mRNA. Alternative splicing at alternative 5′ or 3′ splice sites will result in the formation of extended or shortened exons.

Regulating gene expression is important for numerous cellular processes, and vital for cell survival and overall activity. One important mechanism to regulate expression is by splicing. For the majority of mRNA transcripts, splicing occurs constitutively at standard splice sites, meaning that introns are removed and exons are joined together in their genomic order [1]. In addition to constitutive splicing, alternative splicing (AS) can occur (Figure 1). It is estimated that nearly 95% of genes with multiple exons undergo some form of AS [2]. AS serves two primary functional purposes: first, it provides an additional layer of gene expression control, permitting the precise adjustment of protein levels by regulating the degradation of mRNA, and second, AS can give rise to functionally distinct proteins when alternative mRNA transcripts are translated. AS can be classified into five types (Figure 1). (1) Intron retention leading to the inclusion of intron(s) in the mature mRNA, often preventing translation through nonsense-mediated decay (NMD) and sometimes leading to pre-terminal stop codons yielding truncated products. (2) Mutually exclusive exons leading to mRNA containing either one of two exons but never both simultaneously. (3) Cassette alternative exons, where one exon is spliced out or retained, leading to two different isoforms. (4) Alternative 5′ and (5) 3′ splice sites resulting in either shorter or longer exons, with the 5′ and 3′ giving information about the location of the difference. These forms of alternative splicing have the potential to generate a vast array of protein isoforms from a single gene, each with potential biological and developmental implications due to variations in protein properties such as localization signals, binding capabilities, and enzymatic activity. The precise regulation of AS is crucial for maintaining an accurate expression of mRNA and protein isoforms, ultimately contributing to normal cellular function. Variations in alternative splicing (AS) patterns can manifest in response to environmental and developmental cues, exerting significant influence over biological and developmental processes [3]. AS is controlled by a complex and still incompletely understood process involving many regulatory proteins that together form the spliceosome. The details regarding this process are outside the scope of this manuscript and have been reviewed in detail elsewhere [4]. Although the majority of eukaryotic genes undergo AS, the exact molecular mechanisms underlying the AS of individual mRNAs, and the extent to which AS regulation contributes to cellular and developmental processes in different cell types remain to be further elucidated. Splicing mainly takes place co-transcriptionally and several transcriptional mechanisms of AS regulation are identified. In general, several splicing factors, including serine/arginine-rich splicing factors (SRSF) and heterogeneous nuclear ribonucleoproteins (hnRNPs), and other RNA-binding proteins (RBPs), are present on a single mRNA simultaneously, and ultimately, the balance between splicing enhancing and repressing factors determines AS patterns. It is believed that many AS events are currently unknown; however, novel technologies and bioinformatic methodologies can aid in the description of the full isoform landscape. The recent rise of third-generation RNA sequencing technologies such as PacBio single molecule real-time (SMRT) and Oxford Nanopore Technologies (ONT) sequencing (Box 1) have allowed for a more inclusive and rapid identification of novel transcript isoforms, shedding light on the intricacies of AS within these contexts.

Box 1 **Determining transcript isoforms using third generation RNA sequencing techniques.**

  Third-generation sequencing techniques, such as PacBio single molecule, real-time (SMRT) and Oxford Nanopore Technologies (ONT) sequencing, have revolutionized the field of genomics by enabling high-throughput sequencing of individual native DNA and RNA molecules. In PacBio SMRT sequencing, sequencing units are contained within specialized chambers known as zero-mode waveguides, where fluorescent light is emitted and detected as nucleotides are incorporated into the growing sequences. This process allows for the synthesis-based sequencing of transcripts, with read lengths reaching up to 65 kilobases. Nanopore sequencing utilizes a pore, through which electrical currents are monitored. The passage of nucleotides through the nanopore modulates the electrical signal, and this signal is translated into the corresponding DNA or RNA sequence. Notably, nanopore sequencing is not constrained by read length limitations, making it particularly suitable for obtaining full-length transcript sequences. This characteristic enables the straightforward elucidation of splice patterns, facilitating the generation of isoform-level transcriptome profiles.

### Alternative Splicing as a Post-Transcriptional Regulator of Dynamic Cell Responses

As previously mentioned, alternative splicing is a complexly regulated mechanism allowing for rapid cellular adaptation. There are currently many unidentified isoforms, both in normal physiology and disease states, that convolute our understanding of many pathways, including (but not limited to) immune regulatory pathways. By extending our knowledge regarding these unidentified isoforms, we can increase our understanding of diverse pathways. We will further describe how AS is involved in immune activation, with a focus on how AS is (dys)regulated in immune-mediated arthritis. We will also describe future perspectives for elucidating isoform functionality and potential therapeutic possibilities.

## 2. Alternative Splicing Plays an Important Role in the Immune Response

It is increasingly evident that AS plays a pivotal role in complex cellular systems that necessitate rapid and dynamic alterations in gene expression and function in response to developmental and environmental cues [5]. The immune system serves as a prime example of such a complex system. Here, the detection of pathogens initiates signaling cascades that lead to swift and precise changes in gene expression, crucial for the defense against invading pathogens. AS has been described to be involved in these rapid changes in protein expression and diversity [6]. Despite the growing recognition of AS as a regulator of gene expression during immune responses, the precise molecular mechanisms governing AS in various immune cell types and its regulation throughout different stages of the immune response remain largely unknown. Immune cells can be classified into two major categories: Firstly, innate immune cells, which are responsible for the initial, non-specific defense against invading pathogens, such as monocytes, macrophages, natural killer (NK) cells, and dendritic cells (DCs). The second major category of immune cells are those involved in the adaptive immune response that targets specific antigens, encompassing B and T lymphocytes [7]. Advances in RNA sequencing techniques have allowed for the identification and quantification of AS in various cell types during infections [5,8,9,10]. In this context, several instances of alterations in AS in response to infection are described, along with their functional implications in immune cells [11]. RNA-seq has been used to detect thousands of isoform differences upon activation in various immune cell lineages [12]. Here, AS was demonstrated to play a pivotal role in the maturation and differentiation of hematopoietic precursor cells into fully functional immune cells [12]. One such example is the extensively studied CD45 transcript. CD45 is a transmembrane glycoprotein found on T and B cells. The CD45 transcript can be spliced in eight possible mRNA isoforms, five of which have been observed in humans [13,14,15]. The variation in exon inclusion influences TCR signaling and cytokine production, and is also used to identify B and T cell subsets. B cells have exons 4, 5, and 6, naïve T cells have either 4 or 5, and memory T cells lack all three exons. In cases of aberrant splicing due to a C77G polymorphism in exon 4, splicing is prevented and associated with impaired T cell function and with an increased susceptibility to human immunodeficiency virus infection. This leads to an increased CD45RA, which is typically present on naïve and central memory T cells. The specific regulation of the splicing of CD45 is realized by an interplay of multiple factors and regulators and underscores the intricate nature of changes in splicing patterns during the maturation and differentiation of immune cells. While much remains unknown, there are specific examples in the innate immune system that have been thoroughly elucidated.

### 2.1. Alternative Splicing as a Regulator of Cytokine Expression in Innate Immune Cells

**Figure 2:**
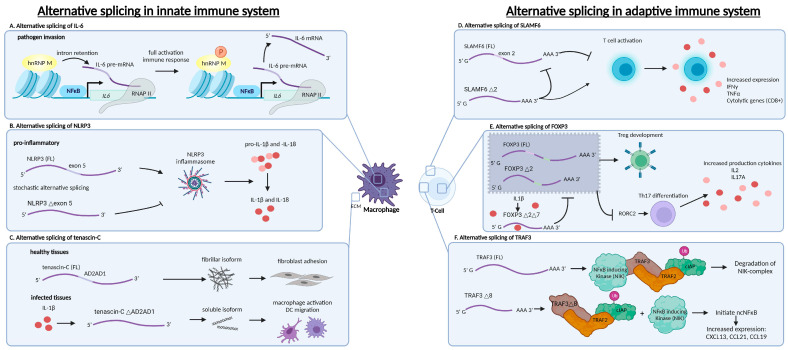
Examples of alternative splicing events that are involved in the innate and adaptive immune response. (**A**) The presence of heterogeneous nuclear ribonucleoproteins (hnRNPs) M on poised chromatin enables immediate interaction with nascent innate immune transcripts, including IL-6. Expression of IL-6 is induced upon the kappa-light-chain-enhancer of B cell (NFκB)-signaling, and the interaction of hnRNP M with the IL-6 pre-mRNAs causes intron retention. This prevents further mRNA processing. Upon full macrophage activation, hnRNP M is phosphorylated and IL-6 transcripts are fully processed and can exit the nucleus. (**B**) In activated macrophages, NLRP3 engages in inflammasome formation and contributes to the cleaving of pro-IL-1β and -IL-18 into functional IL-1β and IL-18. The alternative splicing of NLRP3 results in the generation of short NLRP3 isoforms that lose their pro-inflammatory function. (**C**) IL-1β induces the expression of short isoforms of tenascin-C, which lack the AD2AD1 domain. In contrast to the fibrillar structure of full-length tenascin-C, the short isoform is secreted into the extracellular matrix (ECM) as soluble protein, which activates macrophages and enables dendritic cell (DC) migration. (**D**) SLAMF6 (full length) normally suppresses T cell activation. Absence of exon 2 negates this effect, resulting in T cell activation and subsequent increased expression of cytokines such as IFNγ and TNFα. Additionally, SLAMF6Δ2 inhibits full length SLAMF6. (**E**) FOXP3 (full length) normally inhibits RORC2, thus preventing Th17 differentiation and instead leading T-cells to Treg development. IL-1β induces the alternative splicing of FOXP3 to specifically exclude exon 7 by means of exon skipping. FOXP3(FL) and FOXP3(Δ2) function is inhibited by FOXP3Δ2Δ7, and thus RORC2 is no longer inhibited and T cells will differentiate into Th17 cells with the subsequent production of cytokines IL-2 and IL-17A. (**F**) TRAF3Δ8 is not able to bind NFκB-inducing kinase (NIK), and thus the NIK-complex is not correctly formed and the complex of TRAF3Δ8, TRAF2, and cIAP is degraded. NIK is able to initiate the non-canonical NFκB pathway, which subsequently leads to increased expressions of CXCL13, CCL21, and CCL19.

AS has been demonstrated to be involved in cytokine expression, for instance for IL-6 regulation (Figure 2A) [16]. IL-6 is a central player in host defense and performs various functions, including triggering the acute phase response [17]. In quiescent macrophages, heterogeneous nuclear ribonucleoprotein M (hnRNP M) is poised on chromatin in an inactive state, preventing the maturation of IL-6 transcripts by blocking the removal of introns. Upon even the slightest detection of a pathogen, the nuclear factor kappa-light-chain-enhancer of B cells (NFκB) initiates the transcription of various pro-inflammatory genes, including *IL6*. This will only directly result in a marginal increased expression of IL-6 if hnRNP M is still preventing intron removal. Only upon full activation is the macrophage hnRNP M phosphorylated. Subsequently, the removal of introns is allowed, and mature IL-6 mRNA levels quickly rise for translation. Here, hnRNP M acts as a safeguard, restraining the initial activation of the innate immune response until the pathogen has been fully processed [16].

### 2.2. Alternative Splicing as an Inflammasome Activation Regulator

Additionally, AS has been demonstrated to be involved in inflammasome activation, and more specifically in the NLR family pyrin domain-containing 3 (NLRP3) protein (Figure 2B). NLRP3 is a component of the NLRP3 inflammasome and functions as a pattern recognition receptor. Cell activation results in a switch in AS where exon 5 is included, leading to oligomerization and allowing for the recruitment of apoptosis-associated speck-like proteins (ASC) and pro-caspase-1. Consequently, this active inflammasome complex allows caspase-1 to become activated, thereby cleaving pro-Interleukin-1β (IL-1β) and pro-Interleukin-18 (IL-18) into their active forms. Notably, macrophages have been shown to stochastically transcribe the shorter isoform of NLRP3, which excludes exon 5 [18]. This suggests that AS of NLRP3 may serve as a mechanism for fine-tuning the response of macrophages to pathogenic invasions and regulating caspase-1-mediated programmed cell death in macrophages.

### 2.3. Alternative Splicing as a Regulator of Innate Immune Cell Activation

AS has also been demonstrated to be involved in the activation of innate immune cells through the regulation of the glycoprotein tenascin-c (TNC, Figure 2C) [19]. TNC is a component of the extracellular matrix, and involved in tissue repair, cellular stress, and immune activation. In non-activated DCs and quiescent macrophages, there is a low expression of large TNC isoforms that include the so-called AD2AD1 domains. This protein appears in fibrillar structures and promotes adhesion to fibroblasts and prevents the chemotaxis of DCs and cytokine synthesis by macrophages. Upon activation, IL1β increases the expression of small tenascin-C isoforms, which lack the AD2AD1 domains. This isoform is a secreted soluble form, thereby preventing adhesion to fibroblasts, and consequently DCs can infiltrate the infected tissue and macrophages become activated. The increased expression of these small isoforms is also observed in patients with rheumatoid arthritis (RA) [19]. These examples of AS in innate immune cells demonstrate that AS is required for an efficient innate immune response.

### 2.4. Alternative Splicing as a Regulator of Cytokine Expression in Adaptive Immune Cells

Having discussed some representative examples of AS in innate immune cells, there are also various studies that demonstrate that AS helps to modulate the adaptive immune system [13,15,20,21]. For instance, AS has been demonstrated to be involved in cytokine expression by regulating a member of the SLAM family (SLAMF, Figure 2D) of immune receptors [22]. SLAM receptors are receptors that are capable of activating various downstream pathways. The canonical SLAMF6 transcript is constitutively expressed in T cells. It is hypothesized that this isoform inhibits T cell function by recruiting SLAM associated protein (SAP), thereby effectively preventing cytokine production [22]. In contrast, an alternative SLAM6 isoform that is lacking exon 2 (SLAMF6Δ2) has a co-stimulatory effect on T cell function and leads to increased cytokine expression [22]. This isoform is similar to SLAMF6-FL, regarding transmembrane and intracellular domains, but one major difference is that the immunoglobulin variable is completely absent. Additionally, SLAMF6Δ2 has increased self-binding compared to its SLAMF6-FL counterpart, allowing for the inhibition of SLAMF6-FL. The inhibitory canonical isoform is more prevalent in exhausted T cells that have lost their effector function, such as during chronic inflammation or in some cases of cancer, whereas the shorter isoform is more abundantly present in activated T cells. Given that the splicing ratio of SLAMF6 determines T cell function, manipulating this ratio between the shorter and full-length isoform may be a promising therapeutic approach for treating patients with chronic inflammation or cancer [22].

### 2.5. Alternative Splicing as a Regulator of T Cell Subset Differentiation

AS has been implicated in CD4+ T cell subset differentiation by regulating forkhead box P3 (FOXP3, Figure 2E), which is an essential transcription factor for the function and differentiation of regulatory T (Treg) cells. In unstimulated conditions, full-length FOXP3 stimulates regulatory T cell development and prevents Th17 differentiation through RORC2 inhibition [23,24,25,26]. Upon stimulation and in the presence of IL-1β, exons 2 and 7 of FOXP3 transcripts are spliced out, resulting in a dominant negative isoform. The reason for this is that (1) exon 2 contains an LxxLL motif that interacts with RORA and RORC2 and (2) Exon 7 encodes a leucine zipper domain which is required for FOXP3 dimerization and Treg function [27]. Therefore, T cells are directed toward Th17 differentiation, resulting in IL-2 and IL17A production. The exact mechanisms that regulate the AS of FOXP3 remain unknown.

### 2.6. Alternative Splicing as a Regulator of NFκB Signalling in Adaptive Immune Cells

One final example demonstrates that AS is involved in NFκB signaling by regulating TNF-receptor associated factor 3 (TRAF3, Figure 2F). TRAF3 is an adapter protein and one of the components of the NFκB-inducing kinase complex, together with TRAF2, NFκB-inducing kinase (NIK), and cIAP [28]. In quiescent conditions, NIK binds to TRAF3 which is bound to TRAF2 and cIAP. cIAP is ubiquitinated and therefore the entire complex, including NIK, is degraded. However, upon activation, exon 8 is spliced out. TRAF3Δ8 is unable to bind NIK, and therefore NIK is not degraded with the complex. Then, NIK initiates the non-canonical NFκB pathway by activating IKK-α, which in turn phosphorylates NFKB2, ultimately resulting in the increased expressions of CxCL13, CCL21, and CCL19 [29,30]. In summary, the examples described above underscore the role of AS in the regulation of immune responses during infections. There are only a few detailed examples of AS in the regulation of adaptive immune cells, and much remains to be elucidated. Ideally, a comprehensive atlas should be created of all isoforms in various immune cell subtypes that are exposed to different stimuli. This will provide information about the way that isoforms influence proteins or pathways. This atlas should be extended to include cells from patients with immune-related diseases. Further investigation is necessary to elucidate the mechanisms by which immune activation regulates splicing and the way that alternative splicing is involved in immune-related diseases. In turn, this information can be used to detect biomarkers for immune activation or disease and even assist in designing therapeutic options [31].

## 3. Alternative Splicing in the Scope of Inflammatory Arthritis

AS has been demonstrated to play a significant role in immune cells and has been associated with immune-mediated diseases. Genetic associations have been made between AS and several immune-related diseases, including rheumatoid arthritis (RA). Genome-wide association studies (GWASs) have identified more than 100 genetic loci that link with RA [32]. Whether specific genomic variations could affect splicing can be assessed by splicing quantitative trait loci (sQTLs). sQTLs refer to loci associated with RNA splicing variations and affect the splicing of pre-mRNA, leading to differences in the expression levels of different mRNA isoforms. sQTLs are often located in introns and frequently target global splicing patterns of genes, instead of individual splicing events. sQTLs affect the binding of RBPs by modifying RBP binding sites, thereby altering the splice site strength. There is a significant enrichment of single-nucleotide polymorphisms in sQTLs among disease-associated loci when using the genome-wide association studies data [33]. The top 10 diseases with the largest numbers of genome-wide significantly associated SNPs include multiple sclerosis, psoriasis, and rheumatoid arthritis. Here, a significant enrichment of sQTL SNPs was found among the loci associated with rheumatoid arthritis when excluding MHC (*p* = 0.032, Odds ratio (OR) around 2 when comparing sQTL with non-sQTL, one-tailed Fisher’s exact test with Bonferroni correction) and on the border of significance when including MHC (*p* = 0.064, OR around 1.8). One study examined sQTLs across multiple human tissues and compared this to several diseases [34]. When compared to non-sQTLs, sQTLs displayed a substantial enrichment in variants associated with a wide variety traits and diseases. AS was demonstrated to significantly link immune-related diseases, encompassing several autoimmune disorders and chronic inflammatory conditions such as multiple sclerosis (MS), inflammatory bowel disease (IBD), and arthritis [35,36,37,38,39]. Taken together, these studies indicate that the dysregulation of alternative splicing can contribute to the pathogenesis of immune-related diseases such as rheumatoid arthritis. Gaining a deeper understanding of the specific changes in alternative splicing patterns associated with various diseases could potentially help to understand disease pathogenesis, improve diagnostics, and revolutionize therapeutic strategies [40].

In this review, we focus on inflammatory arthritis, a complex inflammatory disease characterized by the inflammation of one or several joints leading to joint damage and accompanied by pain. Rheumatoid arthritis is the most common form of inflammatory arthritis, with a yearly incidence of 2–5 per 10,000 population in Europe [41]. There are different examples of AS involvement in arthritis pathogenesis, but while significant advances have been made, our understanding of cellular and molecular mechanisms remains limited [32,42].

### 3.1. Alternative Splicing of CD44 in Arthritis

**Figure 3:**
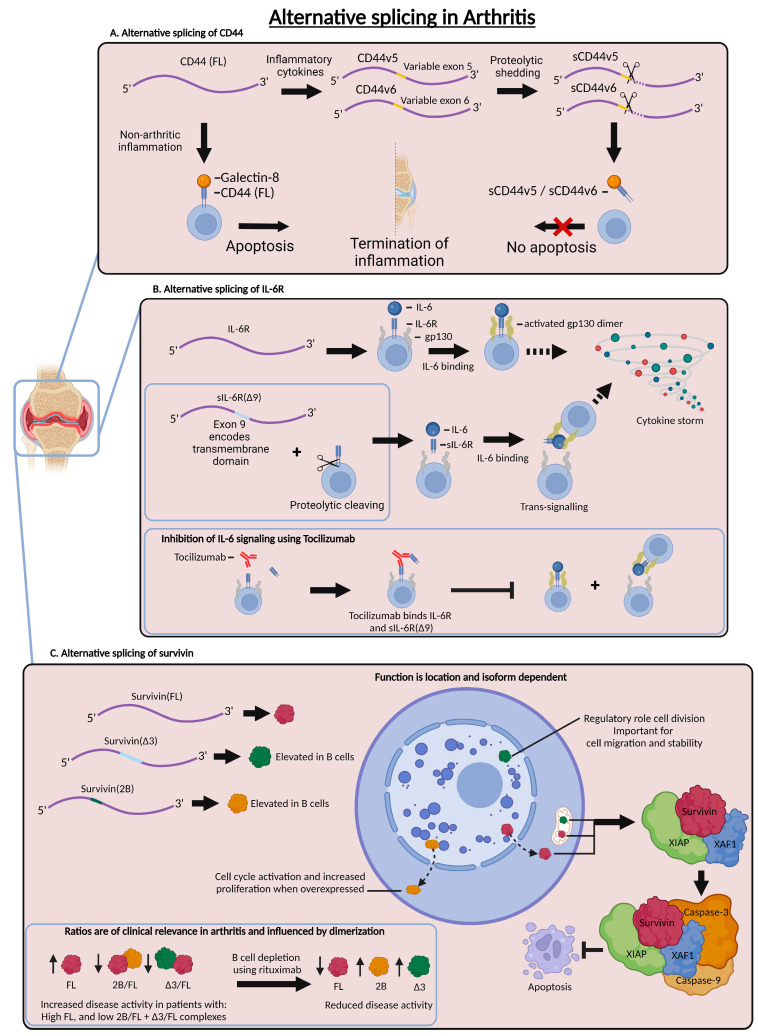
Schematic representation of alternative splicing examples in arthritis. (**A**) Elevated expression levels of CD44v5 and CD44v6 were observed in patients with rheumatoid arthritis. These isoforms were subjected to proteolytic shedding, resulting in soluble isoforms that lack the intracellular signaling domain. Soluble CD44 captures galectin-8 in rheumatoid synovia without subsequently inducing apoptosis, and thus the inflammation will persist. (**B**) In contrast to full-length IL-6R, the delta exon 9 splice variant can be subjected to proteolytic cleaving. Both receptors are overexpressed in patients with arthritis and can induce a cytokine storm upon IL-6 binding and gp130 activation. Tocilizumab binds and inhibits both isoforms of IL-6R and thereby prevents IL-6 signaling. (**C**) Survivin has three different isoforms, FL, Δ3, and 2B. The function of survivin depends on the location and the isoform expressed. Nuclear Δ3 can modulate cell division, migration, and stability. Both FL and Δ3 have anti-apoptotic properties, forming a complex with XIAP and XAF1. This complex binds and inhibits caspase-3 and caspase-9, thereby preventing apoptosis. The 2B can promote cell cycle activation and proliferation. Both Δ3 and 2B are elevated in B cells of arthritis patients, and the ratio between these isoforms influences disease activity. Rituximab-mediated B cell depletion results in reduced FL expression, thereby impairing the expression of FL dimers.

One of the studies that have assessed AS in arthritis focuses on CD44. CD44 is a cell-surface receptor with at least two known ligands, hyaluronic acid and galectin 8 (Figure 3A) [43]. While CD44 is commonly recognized as an important regulator in cancer biology, recent findings indicate its relevance in immunology, where it has many roles, which include regulating apoptosis and inflammation [44,45,46]. CD44 comprises 10 constitutive exons and 10 variable exons. CD44 exhibits significant diversity, with more than 20 isoforms described due to the inclusion or exclusion of the 10 variable exons in various combinations [44,45,47]. In the context of non-arthritic inflammation, the interaction between the full-length CD44 and galectin-8 leads to the induction of apoptosis. However, soluble isoforms, known as sCD44v5 and sCD44v6, exist and have been demonstrated to be expressed in the serum of patients with rheumatoid arthritis. Here, they display elevated expressions compared to both the healthy control and miscellaneous inflammatory rheumatic diseases (MIRD). However, the cellular origin of sCD44 variant isoforms is currently unknown. The presence of sCD44v5 and sCD44v6 correlates with disease severity [48,49]. These isoforms are a product of AS and proteolytic shedding, where the isoforms lose their transmembrane domains. These soluble CD44 isoforms can capture galectin-8 without initiating downstream intracellular signaling pathways. As a result, apoptosis is not induced, and inflammation persists. The use of anti-CD44 monoclonal antibodies has shown a reduction in inflammation in arthritic mice [49]. In summary, the abnormal alternative splicing (AS) of transcripts that encode transmembrane cytokine receptors, in this case CD44, may lead to an imbalance in the expression ratio between surface-bound and soluble isoforms. Similar receptors could be targeted to mitigate pro-inflammatory signaling.

### 3.2. Alternative Splicing of IL-6R in Arthritis

One example of targeting similar receptors to CD44 is the following case. In this case, rheumatoid arthritis (RA) patients are treated with Tocilizumab, a competitive antagonist that targets the interaction between IL-6 and a soluble variant of its receptor IL-6R (sIL-6R, Figure 3B). By blocking the IL-6 interaction with sIL-6R, it prevents a strong pro-inflammatory gp130-mediated signal transduction cascade that can evoke a cytokine storm. sIL-6R is generated from two sources, firstly alternative splicing, which leads to the excision of exon 9, encoding the transmembrane region, and secondly proteases, which can also cleave membrane-bound IL-6R through proteolytic cleaving [26,50]. IL-6 and sIL-6R are both elevated in RA, and the targeting of IL-6 and sIL-6R results in a significant reduction in disease symptoms [50,51,52]. Manipulating the AS of IL-6R to impair the expression of sIL-6R might be worth exploring to reduce the disease burden in these patients.

### 3.3. Alternative Splicing of Survivin in Arthritis

**Figure 4:**
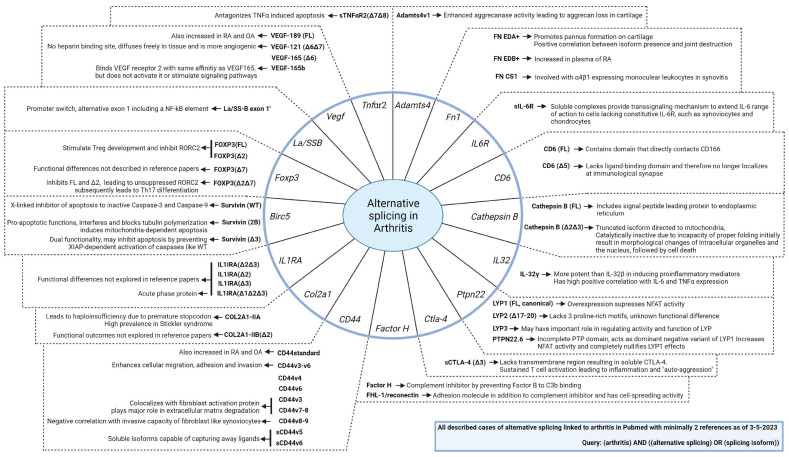
Summarizing overview of alternative splicing events in the scope of arthritis. Known cases of alternative splicing in arthritis are shown in a word web. The gene names are displayed within the outer circle, and corresponding isoforms are described outside of the circle. The splicing isoforms were found using the search query “(arthritis) AND ((alternative splicing) OR (splicing isoform))” on 3rd of May 2023. Isoforms were included when there were at least two references. The references are displayed in a supplementary table and list; respectively, Supplementary SI and SII.

Another case in which AS is involved in RA pertains to survivin (Figure 3C). Survivin is a member of the inhibitor of apoptosis (IAP) family and is encoded by the *BIRC5* gene [53,54]. Survivin has a multitude of functions which depend on both the location and the isoform expressed. The general notion is that survivin is essential for regulating cell division, inhibiting apoptosis and tissue repair. However, the function of survivin differs according to location. Both cytoplasmic and mitochondrial survivin promote cell proliferation and inhibit apoptosis, while nuclear survivin plays a regulatory role in cell division [53]. Survivin can be exported to the cytoplasm from the nucleus to promote anti-apoptotic functions by forming a complex with X-linked IAPs (XIAP). This complex binds and inhibits caspase-3 and -9, thereby effectively inhibiting apoptosis. Mitochondrial survivin binds to pro-apoptotic protein Smac/Diablo, thereby inhibiting the release of Smac/Diablo and thus preventing the activation of caspase-9. In addition to the difference in function based on cellular location, there are also different isoforms exerting different functions. Six different isoforms exist, of which three are the most frequent: full-length survivin (FL), survivin including an additional exon 2 insert (2B), and survivin without exon 3 (Δ3) [53]. While FL and 2B can be actively exported from the nucleus, Δ3 resides in the nucleus as it lacks an export signal. The Δ3 isoform is a dual functional protein that shares anti-apoptotic functions with FL and additionally has roles for cell migration and cell stability. The 2b and Δ3 survivin isoforms can dimerize, forming either homodimers or heterodimers. The function of survivin is determined by the specific dimers that are formed. One study found that CD19^+^ B cells from the peripheral blood of RA patients displayed a high production of 2B and Δ3 compared to healthy controls, whereas the FL levels were similar [53]. These data suggest that it is not the quantity of the individual splice variants but instead the proportional composition between the splice variants that is of clinical relevance in RA. Typically, an excess of FL with low 2B/FL or low Δ3/FL complexes identified patients with increased disease activity. By therapeutically depleting B cells using rituximab, a splice shift can occur, leading to reduced FL and increased 2B and Δ3, which could result in reduced disease activity [53]. While the limited examples above give some insight into the relevance of alternative splicing in the context of arthritis, there is much that remains unknown. It is prudent to compile an inventory of established cases. For this reason, a comprehensive summary (Figure 4) was composed to display the current understanding of alternative splicing in the context of arthritis [2,6,9,10,12,17,21,24,27,30,32,33,34,36,37,38,39,41,42,46,50,51,52,53,54,55,56,57,58,59,60,61,62,63,64,65,66,67,68,69].

## 4. The Clinical Relevance of Alternative Splicing in Arthritis

**Figure 5:**
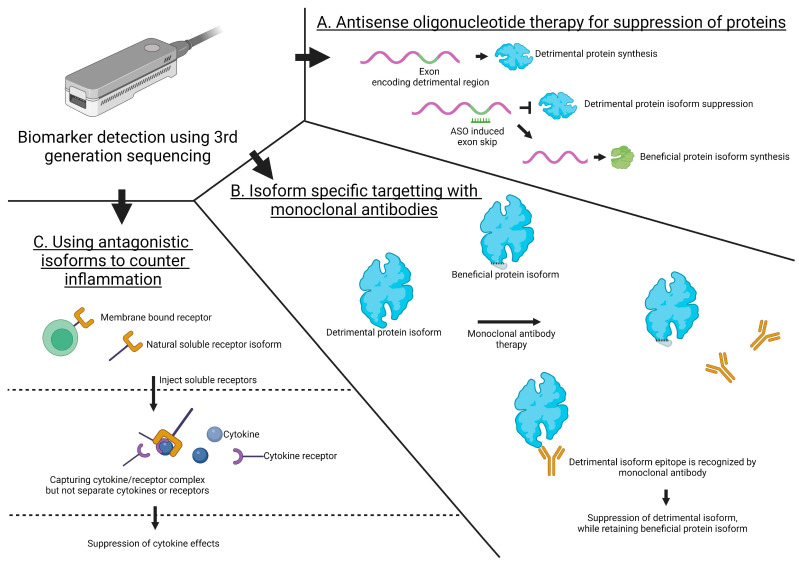
Illustrative examples of potential treatment options based on biomarker detection using 3rd generation sequencing in the scope of arthritis. (**A**) Antisense oligonucleotide (ASO) therapy can be used to induce the specific skipping of a detrimental exon, while maintaining a beneficial protein, depending on the original protein function. (**B**) Monoclonal antibodies capable of recognizing a detrimental protein epitope can be used to specifically target detrimental proteins while retaining beneficial protein isoforms. (**C**) Naturally existing antagonistic isoforms can be used to counter inflammation. Highly purified antagonistic isoforms can be isolated and injected to capture cytokine/receptor complexes, thus suppressing cytokine effects.

Here, we outlined that AS is implicated in immune regulation and in inflammatory arthritis. Mapping changes in how alternative splicing impacts isoform expression and understanding the consequences for immune activation and immune-related diseases could have different clinical implications (Figure 5). More specifically, AS events can be further explored for their therapeutic potential in the development of diagnostic and prognostic biomarkers. Potentially, specific AS events can be targeted with diverse strategies to suppress disease activity. Here, we provide three examples of such strategies.

### 4.1. Antisense Oligonucleotide Therapy for the Suppression of Proteins

Firstly, antisense oligonucleotide (ASO, Figure 5A) therapy can be used to manipulate isoform expression. Here, antisense oligonucleotides can be designed to bind specific splice sites, thereby preventing the binding of the spliceosome and thus skewing splicing towards a specific isoform. This strategy was proved effective in a study that targeted the Tumor Necrosis Factor (TNF) signaling pathway in collagen-induced arthritic (CIA) mouse models [59]. As TNF signaling is directly involved in inflammation, it is interesting to target this pathway. ASOs were utilized to prevent the inclusion of exon 7 in TFN receptor TNFR2. This exon 7 contains the transmembrane domain and therefore TNFR2Δ7 is excreted and capable of capturing circulating TNF-α. Concurrently, the expression of the functional membrane-bound isoform is also reduced, thus offering a two-pronged method to control inflammation. After five days of treatment, the mice displayed a 40% increased survival rate compared to untreated controls. Additionally, these mice displayed significantly reduced paw swelling and reduced clinical scores compared to saline controls. The TNFR2Δ7 protein was actively present in the serum until at least day 50, which was 30 days after the final ASO injection, indicating that it is a stable method for targeting the TNF signaling pathway.

### 4.2. Isoform-Specific Targeting with Monoclonal Antibodies

Secondly, isoform-specific monoclonal antibodies (mAb) can be utilized to detect and bind proteins harboring exons that increase the pro-inflammatory function of specific proteins. One study employed this strategy to target specific adiponectin isoforms and demonstrate their potential for rheumatoid arthritis treatment (Figure 5B). Adiponectin is a hormone protein implicated in the regulation of glucose levels, fatty acid breakdown, and also inflammation [57]. There are three main oligomeric forms, of which two isoforms can increase the expression of chemokines and pro-inflammatory cytokines [70]. Utilizing isoform-specific adiponectin mAbs, a moderate inhibition of the expression of pro-inflammatory cytokines, such as IL-6 and IL-8, was observed in vitro in human cells [70]. Similarly, in a CIA mouse model, injection with these mAbs decreased TNF-α and IL-6 levels; additionally, paw volume and squeaking were reduced, and the anti-arthritic effects were also histologically confirmed.

### 4.3. Utilizing Antagonistic Isoforms to Counter Inflammation

The final therapeutic strategy utilizes naturally existing soluble receptor isoforms (Figure 5C) that are antagonists of cytokine signaling. As recombinant isoforms, they can be administered to disrupt specific pro-inflammatory cytokine/receptor complexes, thereby suppressing cytokine effects. An exemplary case is glycoprotein 130 (gp130) [71]. Gp130 is a member of the IL-6 receptor complex, and has been implicated in many processes such as hematopoiesis, immune response, and inflammation [67]. A recombinant protein was generated from the naturally occurring soluble human gp130 isoform. This soluble isoform acts as an antagonist of IL-6 receptor signaling. In this study, the immunohistochemical analysis of phosphorylated STAT-3 in the synovial tissue of AIA mice demonstrated that soluble gp130 has a concentration-dependent inhibitory effect. This effect was confirmed in vitro in human synovial fibroblasts stimulated with IL-6. Additionally, using soluble gp130 in an experimental murine model of acute peritonitis led to the reduced expression of CCL5, and reduced immune cell recruitment.

### 4.4. Challenges, Limitations, and Future Prospects

The previously mentioned therapeutic examples are just a few of the recent cases that have highlighted the potential of targeting AS and aberrant isoforms in arthritis. It is important to note that there are still technical difficulties to overcome. Some examples of these difficulties include the following: (1) Due to their relatively short length of 15 to 25 nucleotides, ASOs may display some off-target effects in vivo that are difficult to predict [64]. The treatment options must be tested extensively to detect and eliminate the chance of potential off-target effects. One study, using gapmers, short DNA antisense oligonucleotides with RNA-like segments on both sides, showed reduced off-target effects [69]. This study states that this is due to the extension of oligonucleotides. Another study states that the delivery of ASOs can be enhanced by using endosomolytic compounds [55]. These compounds can be used to release ASOs that otherwise accumulate in endosomes. In a similar manner, as there are off-target effects using ASOs, antibodies and antagonistic proteins might also interact with unexpected targets. (2) The in vivo delivery method should be considered thoroughly if these strategies are applied during the treatment of human diseases. These methods listed above have primarily been tested in mouse models or cell lines and might not accurately represent human in vivo conditions, despite biochemical similarities. There might be a difference in stability or efficacy. To conclude, while it is clear that the aforementioned strategies have incredible potential as a therapeutic strategy for immune-related diseases, such as inflammatory arthritis, there are still challenges to overcome that hopefully will be addressed in the near future.

## 5. Conclusions

In this review, we described alternative splicing as a complex post-transcriptional process that is directly implicated in various immunological processes in both the innate and adaptive immune systems. In addition, we discussed AS in the context of autoimmune diseases, specifically in arthritis, and assessed the therapeutic potential of AS manipulation for immune-mediated diseases. Currently there are a small number of splicing modifiers that have been approved by the FDA for treatment of spinal muscular atrophy (SMA) and Duchenne muscular dystrophy (DMD) [65]. These RNA-targeting splicing modifiers open up a plethora of novel treatment options for many diseases, including immune-mediated diseases. In immune cells, the precise functions of many alternatively spliced transcripts still need to be fully understood. Ideally, the third-generation long-read RNA sequencing of specific cell-types in patients and healthy donors may yield valuable information to further elucidate pathways that are currently not well understood. Presently, several efforts are being made to generate a so-called atlas of all isoforms that are expressed in different human immune cells using long-read RNA sequencing [9,10,11,62,66,68]. When AS is better understood in immune activation and immune-mediated diseases, RNA-targeting splicing modifiers can be explored for their potential therapeutic value. In addition, future research focused on understanding how specific single-nucleotide polymorphisms (SNPs) that modulate protein isoform expression can contribute to susceptibility to autoimmune diseases can aid in identifying diagnostic or prognostic biomarkers and the development of personalized therapies. Loci regulating splicing that overlap with genetic risk factors for immune system disorders could serve as valuable starting points for such an approach. There are many steps required to fully understand the complexity of AS and isoform functionality. However, it is clear that there is significant potential in this field.

## Supplementary Materials

The following supporting information can be downloaded at: https://www.mdpi.com/article/10.3390/ijms25158123/s1. Refs. [19,32,38,42,49,52,53,72,73,74,75,76,77,78,79,80,81,82,83,84,85,86,87,88,89,90,91,92,93,94,95,96,97,98,99,100,101,102,103,104,105,106,107,108,109,110,111,112,113,114,115,116,117,118,119,120,121,122,123,124,125,126,127,128,129,130,131,132,133,134,135,136,137,138,139,140,141,142,143,144,145,146,147,148,149,150,151,152,153,154,155,156,157,158,159,160,161,162,163,164,165,166,167,168,169,170,171,172,173,174,175,176,177,178,179,180,181,182,183,184,185,186,187,188,189,190,191,192,193,194,195,196,197,198,199,200,201,202,203,204,205,206,207,208,209,210,211,212,213,214,215,216,217,218,219,220,221,222,223,224,225,226,227,228,229,230,231,232,233,234,235,236,237,238,239,240,241,242,243,244,245,246,247,248,249,250,251,252,253,254,255,256,257,258,259,260,261,262,263,264,265,266,267,268,269,270,271,272,273,274,275,276,277,278,279,280,281,282,283,284,285,286,287,288,289,290,291,292,293,294,295,296,297,298,299,300,301,302,303,304,305,306,307,308,309,310,311,312,313,314,315,316,317,318,319,320,321,322,323,324,325,326,327,328,329,330,331,332,333,334,335,336,337,338,339,340,341,342,343,344,345,346,347,348,349,350,351,352,353,354,355,356,357,358,359,360,361,362,363,364] are cited in Supplementary Materials file.

## References

[1] Wang Y., Liu J., Huang B.O., Xu Y.-M., Li J., Huang L.-F., Lin J., Zhang J., Min Q.-H., Yang W.-M. (2015). Mechanism of alternative splicing and its regulation. Biomed. Rep..

[2] Schaub A., Glasmacher E. (2017). Splicing in immune cells-mechanistic insights and emerging topics. Int. Immunol..

[3] Keren H., Lev-Maor G., Ast G. (2010). Alternative splicing and evolution: Diversification, exon definition and function. Nat. Rev. Genet..

[4] Chen M., Manley J.L. (2009). Mechanisms of alternative splicing regulation: Insights from molecular and genomics approaches. Mol. Cell Biol..

[5] Lynch K.W. (2004). Consequences of regulated pre-mRNA splicing in the immune system. Nat. Rev. Immunol..

[6] Sahoo A., Im S.H. (2010). Interleukin and interleukin receptor diversity: Role of alternative splicing. Int. Rev. Immunol..

[7] Gardiner C.M., Mills K.H.G. (2016). The cells that mediate innate immune memory and their functional significance in inflammatory and infectious diseases. Semin. Immunol..

[8] Martinez N.M., Lynch K.W. (2013). Control of alternative splicing in immune responses: Many regulators, many predictions, much still to learn. Immunol. Rev..

[9] De Bruin R.G., Shiue L., Prins J., de Boer H.C., Singh A., Fagg W.S., van Gils J.M., Duijs J.M., Katzman S., Kraaijeveld A.O. (2016). Quaking promotes monocyte differentiation into pro-atherogenic macrophages by controlling pre-mRNA splicing and gene expression. Nat. Commun..

[10] Liu H., Lorenzini P.A., Zhang F., Xu S., Wong M.S.M., Zheng J., Roca X. (2018). Alternative splicing analysis in human monocytes and macrophages reveals MBNL1 as major regulator. Nucleic Acids Res..

[11] Inamo J., Suzuki A., Ueda M., Yamaguchi K., Nishida H., Suzuki K., Kaneko Y., Takeuchi T., Ishihama Y., Yamamoto K. (2022). Immune Isoform Atlas: Landscape of alternative splicing in human immune cells. bioRxiv.

[12] Ergun A., Doran G., Costello J.C., Paik H.H., Collins J.J., Mathis D., Benoist C., Consortium I., Blair D.A., Dustin M.L. (2013). Differential splicing across immune system lineages. Proc. Natl. Acad. Sci. USA.

[13] Oberdoerffer S., Moita L.F., Neems D., Freitas R.P., Hacohen N., Rao A. (2008). Regulation of CD45 alternative splicing by heterogeneous ribonucleoprotein, hnRNPLL. Science.

[14] Dawes R., Petrova S., Liu Z., Wraith D., Beverley P.C.L., Tchilian E.Z. (2006). Combinations of CD45 isoforms are crucial for immune function and disease. J. Immunol..

[15] Ten Dam G.B., Zilch C.F., Wallace D., Wieringa B., Beverley P.C.L., Poels L.G., Screaton G.R. (2000). Regulation of Alternative Splicing of CD45 by Antagonistic Effects of SR Protein Splicing Factors. J. Immunol..

[16] West K.O., Scott H.M., Torres-Odio S., West A.P., Patrick K.L., Watson R.O. (2019). The Splicing Factor hnRNP M Is a Critical Regulator of Innate Immune Gene Expression in Macrophages. Cell Rep..

[17] Simpson R.J., Hammacher A., Smith D.K., Matthews J.M., Ward L.D. (1997). Interleukin-6: Structure-function relationships. Protein Sci..

[18] Hoss F., Mueller J.L., Rojas Ringeling F., Rodriguez-Alcazar J.F., Brinkschulte R., Seifert G., Stahl R., Broderick L., Putnam C.D., Kolodner R.D. (2019). Alternative splicing regulates stochastic NLRP3 activity. Nat. Commun..

[19] Giblin S.P., Schwenzer A., Midwood K.S. (2020). Alternative splicing controls cell lineage-specific responses to endogenous innate immune triggers within the extracellular matrix. Matrix Biol..

[20] Yabas M., Elliott H., Hoyne G.F. (2015). The Role of Alternative Splicing in the Control of Immune Homeostasis and Cellular Differentiation. Int. J. Mol. Sci..

[21] Banerjee S., Galarza-Munoz G., Garcia-Blanco M.A. (2023). Role of RNA Alternative Splicing in T Cell Function and Disease. Genes..

[22] Hajaj E. (2021). Alternative splicing of SLAMF6 in human T cells creates a co-stimulatory isoform that counteracts the inhibitory effect of the full-length receptor. bioRxiv.

[23] Nik Tavakoli N., Hambly B.D., Sullivan D.R., Bao S. (2008). Forkhead box protein 3: Essential immune regulatory role. Int. J. Biochem. Cell Biol..

[24] Mailer R.K., Joly A.L., Liu S., Elias S., Tegner J., Andersson J. (2015). IL-1beta promotes Th17 differentiation by inducing alternative splicing of FOXP3. Sci. Rep..

[25] McMurchy A.N., Gillies J., Gizzi M.C., Riba M., Manuel Garcia-Manteiga J., Cittaro D., Lazarevic D., Nunzio S.D., Piras I.S., Bulfone A. (2013). A novel function for FOXP3 in humans: Intrinsic regulation of conventional T cells. Blood J. Am. Soc. Hematol..

[26] Zhou L., Lopes J.E., Chong M.M.W., Ivanov I.I., Min R., Victora G.D., Shen Y., Du J., Rubtsov Y.P., Rudensky A.Y. (2008). LETTERS TGF-b-induced Foxp3 inhibits T H 17 cell differentiation by antagonizing RORct function Rorc(gt) gfp/+ CD4 Foxp3 Rorc(gt) gfp/gfp CD4 + GFP int Foxp3 + Foxp3-Foxp3 + Foxp3-CD4 CD4 CD4. Nature.

[27] Du J., Wang Q., Yang S., Chen S., Fu Y., Spath S., Domeier P., Hagin D., Anover-Sombke S., Haouili M. (2022). FOXP3 exon 2 controls T(reg) stability and autoimmunity. Sci. Immunol..

[28] Sun S.-C. (2012). The noncanonical NF-κB pathway. Immunol. Rev..

[29] Michel M., Wilhelmi I., Schultz A.-S., Preussner M., Heyd F. (2014). Activation-induced Tumor Necrosis Factor Receptor-associated Factor 3 (Traf3) Alternative Splicing Controls the Noncanonical Nuclear Factor κB Pathway and Chemokine Expression in Human T Cells. J. Biol. Chem..

[30] Schreuder M.I., van den Brand M., Hebeda K.M., Groenen P., van Krieken J.H., Scheijen B. (2017). Novel developments in the pathogenesis and diagnosis of extranodal marginal zone lymphoma. J. Hematop..

[31] Byun S., Han S., Zheng Y., Planelles V., Lee Y. (2020). The landscape of alternative splicing in HIV-1 infected CD4 T-cells. BMC Med. Genom..

[32] Ren P., Lu L., Cai S., Chen J., Lin W., Han F. (2021). Alternative Splicing: A New Cause and Potential Therapeutic Target in Autoimmune Disease. Front. Immunol..

[33] Takata A., Matsumoto N., Kato T. (2017). Genome-wide identification of splicing QTLs in the human brain and their enrichment among schizophrenia-associated loci. Nat. Commun..

[34] Garrido-Martin D., Borsari B., Calvo M., Reverter F., Guigo R. (2021). Identification and analysis of splicing quantitative trait loci across multiple tissues in the human genome. Nat. Commun..

[35] Evsyukova I., Somarelli J.A., Gregory S.G., Garcia-Blanco M.A. (2010). Alternative splicing in multiple sclerosis and other autoimmune diseases. RNA Biol..

[36] Zhou J., Zhang Q., Zhao Y., Song Y., Leng Y., Chen M., Zhou S., Wang Z. (2023). The regulatory role of alternative splicing in inflammatory bowel disease. Front. Immunol..

[37] Lodde V., Floris M., Zoroddu E., Zarbo I.R., Idda M.L. (2023). RNA-binding proteins in autoimmunity: From genetics to molecular biology. Wiley Interdiscip. Rev. RNA.

[38] Katsoula G., Steinberg J., Tuerlings M., Coutinho de Almeida R., Southam L., Swift D., Meulenbelt I., Wilkinson J.M., Zeggini E. (2022). A molecular map of long non-coding RNA expression, isoform switching and alternative splicing in osteoarthritis. Hum. Mol. Genet..

[39] Zhang Z., Dong L., Tao H., Dong Y., Xiang W., Tao F., Zhao Y. (2024). RNA-binding proteins potentially regulate the alternative splicing of apoptotic genes during knee osteoarthritis progression. BMC Genom..

[40] Su Z., Huang D. (2021). Alternative Splicing of Pre-mRNA in the Control of Immune Activity. Genes.

[41] Lundkvist J., Kastang F., Kobelt G. (2008). The burden of rheumatoid arthritis and access to treatment: Health burden and costs. Eur. J. Health Econ..

[42] Ibanez-Costa A., Perez-Sanchez C., Patino-Trives A.M., Luque-Tevar M., Font P., Arias de la Rosa I., Roman-Rodriguez C., Abalos-Aguilera M.C., Conde C., Gonzalez A. (2022). Splicing machinery is impaired in rheumatoid arthritis, associated with disease activity and modulated by anti-TNF therapy. Ann. Rheum. Dis..

[43] Eshkar Sebban L., Ronen D., Levartovsky D., Elkayam O., Caspi D., Aamar S., Amital H., Rubinow A., Golan I., Naor D. (2007). The Involvement of CD44 and Its Novel Ligand Galectin-8 in Apoptotic Regulation of Autoimmune Inflammation. J. Immunol..

[44] Chen C., Zhao S., Karnad A., Freeman J.W. (2018). The biology and role of CD44 in cancer progression: Therapeutic implications. J. Hematol. Oncol..

[45] Cuff C.A., Puré E. (2001). A crucial role for CD44 in inflammation. TRENDS Mol. Med..

[46] Johnson P., Ruffell B. (2009). CD44 and its role in inflammation and inflammatory diseases. Inflamm. Allergy Drug. Targets.

[47] Barshishat M., Ariel A., Cahalon L., Chowers Y., Lider O., Schwartz B. (2002). TNFα and IL-8 regulate the expression and function of CD44 variant proteins in human colon carcinoma cells. Clin. Exp. Metastasis.

[48] Kittl E.M., Haberhauer G., Ruckser R., Selleny S., Rech-Weichselbraun I., Hinterberger W., Bauer K. (1997). Serum levels of soluble CD44 variant isoforms are elevated in rheumatoid arthritis. Rheumatol. Int..

[49] Naor D., Nedvetzki S. (2003). CD44 in rheumatoid arthritis. Arthritis Res. Ther..

[50] Schumertl T., Lokau J., Rose-John S., Garbers C. (2022). Function and proteolytic generation of the soluble interleukin-6 receptor in health and disease. Biochim. Biophys. Acta Mol. Cell Res..

[51] Hashizume M., Tan S.L., Takano J., Ohsawa K., Hasada I., Hanasaki A., Ito I., Mihara M., Nishida K. (2015). Tocilizumab, a humanized anti-IL-6R antibody, as an emerging therapeutic option for rheumatoid arthritis: Molecular and cellular mechanistic insights. Int. Rev. Immunol..

[52] Lamas J.R., Rodriguez-Rodriguez L., Tornero-Esteban P., Villafuertes E., Hoyas J., Abasolo L., Varade J., Alvarez-Lafuente R., Urcelay E., Fernandez-Gutierrez B. (2013). Alternative splicing and proteolytic rupture contribute to the generation of soluble IL-6 receptors (sIL-6R) in rheumatoid arthritis. Cytokine.

[53] Turkkila M., Andersson K.M., Amu S., Brisslert M., Erlandsson M.C., Silfversward S., Bokarewa M.I. (2015). Suppressed diversity of survivin splicing in active rheumatoid arthritis. Arthritis Res..

[54] Zafari P., Rafiei A., Esmaeili S.A., Moonesi M., Taghadosi M. (2019). Survivin a pivotal antiapoptotic protein in rheumatoid arthritis. J. Cell. Physiol..

[55] Bost J.P., Ojansivu M., Munson M.J., Wesen E., Gallud A., Gupta D., Gustafsson O., Saher O., Radler J., Higgins S.G. (2022). Novel endosomolytic compounds enable highly potent delivery of antisense oligonucleotides. Commun. Biol..

[56] Chatterjee S., Sivanandam V., Wong K.K.M. (2020). Adeno-Associated Virus and Hematopoietic Stem Cells: The Potential of Adeno-Associated Virus Hematopoietic Stem Cells in Genetic Medicines. Hum. Gene Ther..

[57] Choi H.M., Doss H.M., Kim K.S. (2020). Multifaceted Physiological Roles of Adiponectin in Inflammation and Diseases. Int. J. Mol. Sci..

[58] Gil J., Ruiz-Tiscar J.L., Rodriguez-Sainz C., Hernandez A., Santamaria B., Garcia-Sanchez F., Fernandez-Cruz E. (2005). Prevalence of C77G polymorphism in exon 4 of the CD45 gene in the Spanish population. Med. Clin..

[59] Graziewicz M.A., Tarrant T.K., Buckley B., Roberts J., Fulton L., Hansen H., Orum H., Kole R., Sazani P. (2008). An Endogenous TNF-alpha Antagonist Induced by Splice-switching Oligonucleotides Reduces Inflammation in Hepatitis and Arthritis Mouse Models. Mol. Ther..

[60] Hatano H., Ishigaki K. (2023). Functional Genetics to Understand the Etiology of Autoimmunity. Genes.

[61] Kerimov N., Hayhurst J.D., Peikova K., Manning J.R., Walter P., Kolberg L., Samovica M., Sakthivel M.P., Kuzmin I., Trevanion S.J. (2021). A compendium of uniformly processed human gene expression and splicing quantitative trait loci. Nat. Genet..

[62] Ner-Gaon H., Peleg R., Gazit R., Reiner-Benaim A., Shay T. (2023). Mapping the splicing landscape of the human immune system. Front. Immunol..

[63] Robak T., Gladalska A., Stepien H., Robak E. (1998). Serum levels of interleukin-6 type cytokines and soluble interleukin-6 receptor in patients with rheumatoid arthritis. Mediat. Inflamm..

[64] Scharner J., Ma W.K., Zhang Q., Lin K.T., Rigo F., Bennett C.F., Krainer A.R. (2020). Hybridization-mediated off-target effects of splice-switching antisense oligonucleotides. Nucleic Acids Res..

[65] Tang Z., Zhao J., Pearson Z.J., Boskovic Z.V., Wang J. (2021). RNA-Targeting Splicing Modifiers: Drug Development and Screening Assays. Molecules.

[66] Vollmers A.C., Mekonen H.E., Campos S., Carpenter S., Vollmers C. (2021). Generation of an isoform-level transcriptome atlas of macrophage activation. J. Biol. Chem..

[67] White U.A., Stephens J.M. (2011). The gp130 receptor cytokine family: Regulators of adipocyte development and function. Curr. Pharm. Des..

[68] Woolley C.R., Chariker J.H., Rouchka E.C., Ford E.E., Hudson E.A., Waigel S.J., Smith M.L., Mitchell T.C. (2022). Reference long-read isoform-aware transcriptomes of 4 human peripheral blood lymphocyte subsets. G3.

[69] Yasuhara H., Yoshida T., Sasaki K., Obika S., Inoue T. (2022). Reduction of Off-Target Effects of Gapmer Antisense Oligonucleotides by Oligonucleotide Extension. Mol. Diagn. Ther..

[70] Lee Y.A., Hahm D.H., Kim J.Y., Sur B., Lee H.M., Ryu C.J., Yang H.I., Kim K.S. (2018). Potential therapeutic antibodies targeting specific adiponectin isoforms in rheumatoid arthritis. Arthritis Res. Ther..

[71] Richards P.J., Nowell M.A., Horiuchi S., McLoughlin R.M., Fielding C.A., Grau S., Yamamoto N., Ehrmann M., Rose-John S., Williams A.S. (2006). Functional characterization of a soluble gp130 isoform and its therapeutic capacity in an experimental model of inflammatory arthritis. Arthritis Rheum..

[72] Midwood K.S., Chiquet M., Tucker R.P., Orend G. (2016). Tenascin-C at a glance. J. Cell Sci..

[73] Wan Y., Anastasakis D.G., Rodriguez J., Palangat M., Gudla P., Zaki G., Tandon M., Pegoraro G., Chow C.C., Hafner M. (2021). Dynamic imaging of nascent RNA reveals general principles of transcription dynamics and stochastic splice site selection. Cell.

[74] Donaldson L.F., Beazley-Long N. (2016). Alternative RNA splicing: Contribution to pain and potential therapeutic strategy. Drug Discov. Today.

[75] Fan X., Tang L. (2013). Aberrant and alternative splicing in skeletal system disease. Gene.

[76] Aravilli R.K., Vikram S.L., Kohila V. (2021). The Functional Impact of Alternative Splicing and Single Nucleotide Polymorphisms in Rheumatoid Arthritis. Curr. Pharm. Biotechnol..

[77] Lee Y., Wessel A.W., Xu J., Reinke J.G., Lee E., Kim S.M., Hsu A.P., Zilberman-Rudenko J., Cao S., Enos C. (2022). Genetically programmed alternative splicing of NEMO mediates anautoinflammatory disease phenotype. J. Clin. Investig..

[78] Poirier E.Z., Buck M.D., Chakravarty P., Carvalho J., Frederico B., Cardoso A., Healy L., Ulferts R., Beale R., Reis e Sousa C. (2021). An isoform of Dicer protects mammalianstem cells against multiple RNA viruses. Science.

[79] Liu Z., Wang H., Hou Y., Yang Y., Jia J., Wu J., Zuo Z., Gao T., Ren S., Bian Y. (2021). CNC-bZIP protein NFE2L1 regulates osteoclast differentiation in antioxidant-dependent and independent manners. Redox Biol..

[80] Rose K.W.J., Taye N., Karoulias S.Z., Hubmacher D. (2021). Regulation of ADAMTS Proteases. Front. Mol. Biosci..

[81] Romberger D.J. (1997). Fibronectin. Int. J. Biochem. Cell Biol..

[82] Clanchy F.I.L., Borghese F., Bystrom J., Balog A., Penn H., Taylor P.C., Stone T.W., Mageed R.A., Williams R.O. (2022). Disease status in human and experimental arthritis, and response to TNF blockade, is associated with MHC class II invariant chain (CD74) isoform expression. J. Autoimmun..

[83] Heinhuis B., Koenders M.I., van de Loo F.A., Netea M.G., van den Berg W.B., Joosten L.A. (2011). Inflammation-dependent secretion and splicing of IL-32(gamma) in rheumatoid arthritis. Proc. Natl. Acad. Sci. USA.

[84] Baici A., Müntener K., Willimann A., Zwicky R. (2006). Regulation of human cathepsin B by alternative mRNA splicing: Homeostasis, fatal errors and cell death. Biol. Chem..

[85] Shchetynsky K., Protsyuk D., Ronninger M., Diaz-Gallo L.M., Klareskog L., Padyukov L. (2015). Gene-gene interaction and RNA splicing profiles of MAP2K4 gene in rheumatoid arthritis. Clin. Immunol..

[86] Muys B.R., Anastasakis D.G., Claypool D., Pongor L., Li X.L., Grammatikakis I., Liu M., Wang X., Prasanth K.V., Aladjem M.I. (2021). The p53-induced RNA-binding protein ZMAT3 is a splicing regulator that inhibits the splicing of oncogenic CD44 variants in colorectal carcinoma. Genes Dev..

[87] Santiago B., Izquierdo E., Rueda P., Del Rey M.J., Criado G., Usategui A., Arenzana-Seisdedos F., Pablos J.L. (2012). CXCL12γ isoform is expressed on endothelial and dendritic cells in rheumatoid arthritis synovium and regulates T cell activation. Arthritis Rheum..

[88] Cao W., He W. (2004). UL16 binding proteins. Immunobiology.

[89] Mobasheri A., Matta C., Uzielienè I., Budd E., Martín-Vasallo P., Bernotiene E. (2019). The chondrocyte channelome: A narrative review. Jt. Bone Spine.

[90] Lu S., Borst D.E., Horowits R. (2008). Expression and alternative splicing of N-RAP during mouse skeletal muscle development. Cell Motil. Cytoskelet..

[91] Berardi S., Lang A., Kostoulas G., Hörler D., Vilei E.M., Baici A. (2001). Alternative messenger RNA splicing and enzyme forms of cathepsin B in human osteoarthritic cartilage and cultured chondrocytes. Arthritis Rheum..

[92] Da Glória V.G., Martins de Araújo M., Mafalda Santos A., Leal R., de Almeida S.F., Carmo A.M., Moreira A. (2014). T cell activation regulates CD6 alternative splicing by transcription dynamics and SRSF1. J. Immunol..

[93] Bondos S.E., Geraldo Mendes G., Jons A. (2020). Context-dependent HOX transcription factor function in health and disease. Prog. Mol. Biol. Transl. Sci..

[94] Holland D.O., Gotea V., Fedkenheuer K., Jaiswal S.K., Baugher C., Tan H., Fedkenheuer M., Elnitski L. (2022). Characterization and clustering of kinase isoform expression in metastatic melanoma. PLoS Comput. Biol..

[95] Lin E.A., Liu C.J. (2010). The role of ADAMTSs in arthritis. Protein Cell.

[96] Giannopoulou E.G., Elemento O., Ivashkiv L.B. (2015). Use of RNA sequencing to evaluate rheumatic disease patients. Arthritis Res. Ther..

[97] Jordan A.R., Racine R.R., Hennig M.J., Lokeshwar V.B. (2015). The Role of CD44 in Disease Pathophysiology and Targeted Treatment. Front. Immunol..

[98] Gill R.B., Day A., Barstow A., Zaman G., Chenu C., Dhoot G.K. (2012). Mammalian Sulf1 RNA alternative splicing and its significance to tumour growth regulation. Tumour Biol..

[99] Sarkissian M., Winne A., Lafyatis R. (1996). The mammalian homolog of suppressor-of-white-apricot regulates alternative mRNA splicing of CD45 exon 4 and fibronectin IIICS. J. Biol. Chem..

[100] Chang H.H., Tai T.S., Lu B., Iannaccone C., Cernadas M., Weinblatt M., Shadick N., Miaw S.C., Ho I.C. (2012). PTPN22.6, a dominant negative isoform of PTPN22 and potential biomarker of rheumatoid arthritis. PLoS ONE.

[101] Torreggiani S., Torcoletti M., Campos-Xavier B., Baldo F., Agostoni C., Superti- Furga A., Filocamo G. (2019). Progressive pseudorheumatoid dysplasia: A rare childhood disease. Rheumatol. Int..

[102] Porola P., Mackiewicz Z., Laine M., Baretto G., Stegaev V., Takakubo Y., Takagi M., Ainola M., Konttinen Y.T. (2011). Laminin isoform profiles in salivary glands in Sjögren’s syndrome. Adv. Clin. Chem..

[103] Chalaris A., Garbers C., Rabe B., Rose-John S., Scheller J. (2011). The soluble Interleukin 6 receptor: Generation and role in inflammation and cancer. Eur. J. Cell Biol..

[104] Tardif G., Dupuis M., Reboul P., Geng C.S., Pelletier J.P., Ranger P., Martel-Pelletier J. (2003). Identification and differential expression of human collagenase-3 mRNA species derived from internal deletion, alternative splicing, and different polyadenylation and transcription initiation sites. Osteoarthr. Cartil..

[105] Kozyrev S.V., Alarcon-Riquelme M.E. (2007). The genetics and biology of Irf5-mediated signaling in lupus. Autoimmunity.

[106] Kim S. (2014). Interleukin-32 in inflammatory autoimmune diseases. Immune Netw..

[107] Morand E.F. (2007). Effects of glucocorticoids on inflammation and arthritis. Curr. Opin. Rheumatol..

[108] Li H.Z., Lin Z., Xu X.H., Lin N., Lu H.D. (2018). The potential roles of circRNAs in osteoarthritis: A coming journey to find a treasure. Biosci. Rep..

[109] Christmas P., Ursino S.R., Fox J.W., Soberman R.J. (1999). Expression of the CYP4F3 gene. tissue-specific splicing and alternative promoters generate high and low K(m) forms of leukotriene B(4) omega-hydroxylase. J. Biol. Chem..

[110] Arnér E.S. (2009). Focus on mammalian thioredoxin reductases—Important selenoproteins with versatile functions. Biochim. Biophys. Acta.

[111] Frisch R.N., Curtis K.M., Aenlle K.K., Howard G.A. (2016). Hepatocyte growth factor and alternative splice variants-expression, regulation and implications in osteogenesis and bone health and repair. Expert. Opin. Ther. Targets.

[112] Fan Y., Yang J., Xie S., He J., Huang S., Chen J., Jiang S., Yu L., Zhou Y., Cao X. (2022). Systematic analysis of inflammation and pain pathways in a mouse model of gout. Mol. Pain.

[113] Scheller J., Garbers C., Rose-John S. (2014). Interleukin-6: From basic biology to selective blockade of pro-inflammatory activities. Semin. Immunol..

[114] Hersh E.V., Lally E.T., Moore P.A. (2005). Update on cyclooxygenase inhibitors: Has a third COX isoform entered the fray?. Curr. Med. Res. Opin..

[115] Yan S., Sloane B.F. (2003). Molecular regulation of human cathepsin B: Implication in pathologies. Biol. Chem..

[116] Odhams C.A., Cortini A., Chen L., Roberts A.L., Viñuela A., Buil A., Small K.S., Dermitzakis E.T., Morris D.L., Vyse T.J. (2017). Mapping eQTLs with RNA-seq reveals novel susceptibility genes, non-coding RNAs and alternative-splicing events in systemic lupus erythematosus. Hum. Mol. Genet..

[117] Hashimoto T., Yasuda S., Koide H., Kataoka H., Horita T., Atsumi T., Koike T. (2011). Aberrant splicing of the hRasGRP4 transcript and decreased levels of this signaling protein in the peripheral blood mononuclear cells in a subset of patients with rheumatoid arthritis. Arthritis Res. Ther..

[118] Baiyasi A., Barbosa J., Parendo A., Lin X. (2022). Pleiotropy of a Stickler syndrome genotype. Eur. J. Ophthalmol..

[119] Logsdon C.D., Fuentes M.K., Huang E.H., Arumugam T. (2007). RAGE and RAGE ligands incancer. Curr. Mol. Med..

[120] Ward F.J., Dahal L.N., Khanolkar R.C., Shankar S.P., Barker R.N. (2014). Targeting the alternatively spliced soluble isoform of CTLA-4: Prospects for immunotherapy?. Immunotherapy.

[121] Ball A.K., Beilstein K., Wittmann S., Sürün D., Saul M.J., Schnütgen F., Flamand N., Capelo R., Kahnt A.S., Frey H. (2017). Characterization and cellular localization of human 5-lipoxygenase and its protein isoforms 5-LOΔ13, 5-LOΔ4 and 5-LOp12. Biochim. Biophys. Acta Mol. Cell Biol. Lipids.

[122] Liu Q., Niu N., Wada Y., Liu J. (2016). The Role of Cdkn1A-Interacting Zinc Finger Protein 1 (CIZ1) in DNA Replication and Pathophysiology. Int. J. Mol. Sci..

[123] Naor D., Sionov R.V., Ish-Shalom D. (1997). CD44: Structure, function, and association with the malignant process. Adv. Cancer Res..

[124] Nollet M., Bachelier R., Joshkon A., Traboulsi W., Mahieux A., Moyon A., Muller A., Somasundaram I., Simoncini S., Peiretti F. (2022). Involvement of Multiple Variants of Soluble CD146 in Systemic Sclerosis: Identification of a Novel Profibrotic Factor. Arthritis Rheumatol..

[125] Santos R.F., Oliveira L., Carmo A.M. (2016). Tuning T Cell Activation: The Function of CD6 At the Immunological Synapse and in T Cell Responses. Curr. Drug Targets.

[126] Dinarello C.A. (2000). Targeting interleukin 18 with interleukin 18 binding protein. Ann. Rheum. Dis..

[127] Cao X., Li P., Song X., Shi L., Qin L., Chen D., Chu T., Cheng Y. (2022). PCBP1 isassociated with rheumatoid arthritis by affecting RNA products of genes involved in immune response in Th1 cells. Sci. Rep..

[128] Barbezier N., Tessier F.J., Chango A. (2014). Le récepteur des produits de glycation avancée RAGE/AGER: Une vue intégrative pour des applications en clinique [Receptor of advanced glycation endproducts RAGE/AGER: An integrative view for clinical applications]. Ann. Biol. Clin..

[129] Müller-Ladner U., Elices M.J., Kriegsmann J.B., Strahl D., Gay R.E., Firestein G.S., Gay S. (1997). Alternatively spliced CS-1 fibronectin isoform and its receptor VLA-4 in rheumatoid arthritis synovium. J. Rheumatol..

[130] Atsumi T., Suzuki H., Jiang J.J., Okuyama Y., Nakagawa I., Ota M., Tanaka Y., Ohki T., Katsunuma K., Nakajima K. (2017). Rbm10 regulates inflammation development via alternative splicing of Dnmt3b. Int. Immunol..

[131] Geroldi D., Falcone C., Emanuele E. (2006). Soluble receptor for advanced glycation end products: From disease marker to potential therapeutic target. Curr. Med. Chem..

[132] Lin J.D., Chao T.C. (2005). Vascular endothelial growth factor in thyroid cancers. Cancer Biother. Radiopharm..

[133] Hull K.M., Drewe E., Aksentijevich I., Singh H.K., Wong K., McDermott E.M., Dean J., Powell R.J., Kastner D.L. (2002). The TNF receptor-associated periodic syndrome (TRAPS): Emerging concepts of an autoinflammatory disorder. Medicine.

[134] Wittig B.M., Stallmach A., Zeitz M., Günthert U. (2003). Functional involvement of CD44 variant 7 in gut immune response. Pathobiology.

[135] Rose-John S., Waetzig G.H., Scheller J., Grötzinger J., Seegert D. (2007). The IL-6/sIL-6R complex as a novel target for therapeutic approaches. Expert. Opin. Ther. Targets.

[136] Du J., Qiao Y., Sun L., Wang X. (2014). Lymphoid-specific tyrosine phosphatase (Lyp): A potential drug target for treatment of autoimmune diseases. Curr. Drug Targets.

[137] Ebbinghaus C., Scheuermann J., Neri D., Elia G. (2004). Diagnostic and therapeutic applications of recombinant antibodies: Targeting the extra-domain B of fibronectin, a marker of tumor angiogenesis. Curr. Pharm. Des..

[138] Cañete J.D., Albaladejo C., Hernández M.V., Laínez B., Pinto J.A., Ramírez J., López- Armada M.J., Rodríguez-Cros J.R., Engel P., Blanco F.J. (2011). Clinical significance of high levels of soluble tumour necrosis factor-α receptor-2 produced by alternative splicing in rheumatoid arthritis: A longitudinal prospective cohort study. Rheumatology.

[139] Brenol C.V., Veit T.D., Chies J.A., Xavier R.M. (2012). The role of the HLA-G gene and molecule on the clinical expression of rheumatologic diseases. Rev. Bras. Reumatol..

[140] Hanson A.L., Cuddihy T., Haynes K., Loo D., Morton C.J., Oppermann U., Leo P., Thomas G.P., Lê Cao K.A., Kenna T.J. (2018). Genetic Variants in ERAP1 and ERAP2 Associated With Immune-Mediated Diseases Influence Protein Expression and the Isoform Profile. Arthritis Rheumatol..

[141] Friedman B., Larranaga-Vera A., Castro C.M., Corciulo C., Rabbani P., Cronstein B.N. (2023). Adenosine A2A receptor activation reduces chondrocyte senescence. FASEB J..

[142] Mulcahy H., O’Rourke K.P., Adams C., Molloy M.G., O’Gara F. (2006). LST1 and NCR3 expression in autoimmune inflammation and in response to IFN-gamma, LPS and microbial infection. Immunogenetics.

[143] Micheau O. (2003). Cellular FLICE-inhibitory protein: An attractive therapeutic target?. Expert. Opin. Ther. Targets.

[144] Suzuki A., Terao C., Yamamoto K. (2019). Linking of genetic risk variants to disease-specific gene expression via multi-omics studies in rheumatoid arthritis. Semin. Arthritis Rheum..

[145] Tang Y.P., Zhang Q.B., Dai F., Liao X., Dong Z.R., Yi T., Qing Y.F. (2021). Circular RNAs in peripheral blood mononuclear cells from ankylosing spondylitis. Chin. Med. J..

[146] Yoon H.K., Byun H.S., Lee H., Jeon J., Lee Y., Li Y., Jin E.H., Kim J., Hong J.H., Kim J.H. (2013). Intron-derived aberrant splicing of A20 transcript in rheumatoid arthritis. Rheumatology.

[147] Błochowiak K.J., Trzybulska D., Olewicz-Gawlik A., Sikora J.J., Nowak-Gabryel M., Kocięcki J., Witmanowski H., Sokalski J. (2018). Levels of EGF and VEGF in patients with primary and secondary Sjögren’s syndrome. Adv. Clin. Exp. Med..

[148] Nesterovitch A.B., Hoffman M.D., Simon M., Petukhov P.A., Tharp M.D., Glant T.T. (2011). Mutations in the PSTPIP1 gene and aberrant splicing variants in patients with pyoderma gangrenosum. Clin. Exp. Dermatol..

[149] Naor D., Nedvetzki S., Walmsley M., Yayon A., Turley E.A., Golan I., Caspi D., Sebban L.E., Zick Y., Garin T. (2007). CD44 involvement in autoimmune inflammations: The lesson to be learned from CD44-targeting by antibody or from knockout mice. Ann. N. Y. Acad. Sci..

[150] Liu X., Dong H., Gong Y., Wang L., Zhang R., Zheng T., Zheng Y., Shen S., Zheng C., Tian M. (2022). A Novel missense mutation of COL2A1 gene in a large family with stickler syndrome type I. J. Cell. Mol. Med..

[151] Etem E.O., Koca S.S., Erol D., Yolbas S., Oz E., Elyas H., Isık A. (2015). Decreased MEFV gene expression in rheumatoid arthritis patients. Genet. Mol. Res..

[152] Ramos H.L., O’Shea J.J., Watford W.T. (2007). STAT5 isoforms: Controversies and clarifications. Biochem. J..

[153] Peffers M.J., Fang Y., Cheung K., Wei T.K., Clegg P.D., Birch H.L. (2015). Transcriptome analysis of ageing in uninjured human Achilles tendon. Arthritis Res. Ther..

[154] Yoo S.A., Leng L., Kim B.J., Du X., Tilstam P.V., Kim K.H., Kong J.S., Yoon H.J., Liu A., Wang T. (2016). MIF allele-dependent regulation of the MIF coreceptor CD44 and role in rheumatoid arthritis. Proc. Natl. Acad. Sci. USA.

[155] Devauchelle V., Essabbani A., De Pinieux G., Germain S., Tourneur L., Mistou S., Margottin-Goguet F., Anract P., Migaud H., Le Nen D. (2006). Characterization and functional consequences of underexpression of clusterin in rheumatoid arthritis. J. Immunol..

[156] Huang F., Yamaguchi A., Tsuchiya N., Ikawa T., Tamura N., Virtala M.M., Granfors K., Yasaei P., Yu D.T. (1997). Induction of alternative splicing of HLA-B27 by bacterialinvasion. Arthritis Rheum..

[157] Venkatachalam K.V. (2003). Human 3’-phosphoadenosine 5’-phosphosulfate (PAPS)synthase: Biochemistry, molecular biology and genetic deficiency. IUBMB Life.

[158] Lemaire R., Winne A., Sarkissian M., Lafyatis R. (1999). SF2 and SRp55 regulation of CD45 exon 4 skipping during T cell activation. Eur. J. Immunol..

[159] Hulse R.P., Drake R.A., Bates D.O., Donaldson L.F. (2016). The control of alternativesplicing by SRSF1 in myelinated afferents contributes to the development of neuropathic pain. Neurobiol. Dis..

[160] Muys B.R., Shrestha R.L., Anastasakis D.G., Pongor L., Li X.L., Grammatikakis I., Polash A., Chari R., Gorospe M., Harris C.C. (2023). Matrin3 regulates mitotic spindle dynamics by controlling alternative splicing of CDC14B. Cell Rep..

[161] Rong J., Yin J., Su Z. (2015). Natural antisense RNAs are involved in the regulation of CD45 expression in autoimmune diseases. Lupus.

[162] Crawford A.H., Hildyard J.C.W., Rushing S.A.M., Wells D.J., Diez-Leon M., Piercy R.J. (2022). Validation of DE50-MD dogs as a model for the brain phenotype of Duchenne muscular dystrophy. Dis. Model. Mech..

[163] Qin Z., Qin L., Feng X., Li Z., Bian J. (2021). Development of Cdc2-like Kinase 2 Inhibitors: Achievements and Future Directions. J. Med. Chem..

[164] Hitomi Y., Aiba Y., Ueno K., Nishida N., Kawai Y., Kawashima M., Tsuiji M., Iwabuchi C., Takada S., Miyake N. (2022). rs2013278 in the multiple immunological-trait susceptibility locus CD28 regulates the production of non-functional splicing isoforms. Hum. Genom..

[165] Ramsay R.G., Ciznadija D., Vanevski M., Mantamadiotis T. (2003). Transcriptional regulation of cyclo-oxygenase expression: Three pillars of control. Int. J. Immunopathol. Pharmacol..

[166] Aigner T., Bartnik E., Sohler F., Zimmer R. (2004). Functional genomics of osteoarthritis: On the way to evaluate disease hypotheses. Clin. Orthop. Relat. Res..

[167] Jones D.C., Roghanian A., Brown D.P., Chang C., Allen R.L., Trowsdale J., Young N.T. (2009). Alternative mRNA splicing creates transcripts encoding soluble proteins from most LILR genes. Eur. J. Immunol..

[168] Evans C.H., Robbins P.D. (1994). The interleukin-1 receptor antagonist and its delivery by gene transfer. Receptor.

[169] Choi J.D., Bae S.Y., Hong J.W., Azam T., Dinarello C.A., Her E., Choi W.S., Kim B.K., Lee C.K., Yoon D.Y. (2009). Identification of the most active interleukin-32 isoform. Immunology.

[170] Nichols R.C., Raben N., Boerkoel C.F., Plotz P.H. (1995). Human isoleucyl-tRNA synthetase: Sequence of the cDNA, alternative mRNA splicing, and the characteristics of an unusually long C-terminal extension. Gene.

[171] Palangat M., Anastasakis D.G., Fei D.L., Lindblad K.E., Bradley R., Hourigan C.S., Hafner M., Larson D.R. (2019). The splicing factor U2AF1 contributes to cancer progression through a noncanonical role in translation regulation. Genes Dev..

[172] Ward F.J., Dahal L.N., Wijesekera S.K., Abdul-Jawad S.K., Kaewarpai T., Xu H., Vickers M.A., Barker R.N. (2013). The soluble isoform of CTLA-4 as a regulator of T-cell responses. Eur. J. Immunol..

[173] Gouttenoire J., Valcourt U., Ronzière M.C., Aubert-Foucher E., Mallein-Gerin F., Herbage D. (2004). Modulation of collagen synthesis in normal and osteoarthritic cartilage. Biorheology.

[174] Bottini N., Bottini E., Gloria-Bottini F., Mustelin T. (2002). Low-molecular-weight protein tyrosine phosphatase and human disease: In search of biochemical mechanisms. Arch. Immunol. Ther. Exp..

[175] Sterenczak K.A., Willenbrock S., Barann M., Klemke M., Soller J.T., Eberle N., Nolte I., Bullerdiek J., Murua Escobar H. (2009). Cloning, characterisation, and comparative quantitative expression analyses of receptor for advanced glycation end products (RAGE) transcript forms. Gene.

[176] Hattori M., Yabuuchi A., Tanaka H., Kawara T., Wang H., Inoue K., Shiozawa S., Komai K. Expression of ASC splice variant found in Japanese patients with palindromic rheumatism is regulated by rs8056505 single nucleotide polymorphism and interleukin-1 beta. Asian Pac. J. Allergy Immunol..

[177] Wang S., Dong H., Han J., Ho W.T., Fu X., Zhao Z.J. (2010). Identification of a variant form of tyrosine phosphatase LYP. BMC Mol. Biol..

[178] Ichinose K., Zhang Z., Koga T., Juang Y.T., Kis-Tóth K., Sharpe A.H., Kuchroo V., Crispín J.C., Tsokos G.C. (2013). Brief report: Increased expression of a short splice variant of CTLA-4 exacerbates lupus in MRL/lpr mice. Arthritis Rheum..

[179] Watanabe H., Kuroki K., Yamada C., Saburi Y., Maeda N., Maenaka K. (2020). Therapeutic effects of soluble human leukocyte antigen G2 isoform in lupus-prone MRL/lpr mice. Hum. Immunol..

[180] Ramírez-Bello J., Vargas-Alarcón G., Tovilla-Zárate C., Fragoso J.M. (2013). Polimorfismos de un solo nucleótido (SNP): Implicaciones funcionales de los SNP reguladores (rSNP) y de los SNP-ARN estructurales (srSNP) en enfermedades complejas [Single nucleotide polymorphisms (SNPs): Functional implications of regulatory-SNP (rSNP) and structural RNA (srSNPs) in complex diseases]. Gac Med. Mex..

[181] Nakajima M., Miyamoto Y., Ikegawa S. (2011). Cloning and characterization of the osteoarthritis-associated gene DVWA. J. Bone Miner. Metab..

[182] Van Hoolwerff M., Tuerlings M., Wijnen I.J.L., Suchiman H.E.D., Cats D., Mei H., Nelissen R.G.H.H., van der Linden-van der Zwaag H.M.J., Ramos Y.F.M., Coutinho de Almeida R. (2023). Identification and functional characterization of imbalanced osteoarthritis-associated fibronectin splice variants. Rheumatology.

[183] Turner M.W., Hamvas R.M. (2000). Mannose-binding lectin: Structure, function, genetics and disease associations. Rev. Immunogenet..

[184] Parker A.E., Boutell J., Carr A., Maciewicz R.A. (2002). Novel cartilage-specific splice variants of fibronectin. Osteoarthr. Cartil..

[185] Sztrolovics R., Grover J., Cs-Szabo G., Shi S.L., Zhang Y., Mort J.S., Roughley P.J. (2002). The characterization of versican and its message in human articular cartilage and intervertebral disc. J. Orthop. Res..

[186] Peffers M.J., Collins J., Fang Y., Goljanek-Whysall K., Rushton M., Loughlin J., Proctor C., Clegg P.D. (2016). Age-related changes in mesenchymal stem cells identified using a multi-omics approach. Eur. Cell Mater..

[187] Ryder L.R., Bartels E.M., Woetmann A., Madsen H.O., Odum N., Bliddal H., Danneskiold-Samsøe B., Ribel-Madsen S., Ryder L.P. (2012). FoxP3 mRNA splice forms in synovial CD4+ T cells in rheumatoid arthritis and psoriatic arthritis. APMIS.

[188] Toussirot E., Saas P., Deschamps M., Pouthier F., Perrot L., Perruche S., Chabod J., Tiberghien P., Wendling D. (2009). Increased production of soluble CTLA-4 in patients with spondylarthropathies correlates with disease activity. Arthritis Res. Ther..

[189] Lee S., Kim S., Bae S., Choi J., Hong J., Ryoo S., Jhun H., Hong K., Kim E., Jo S. (2010). Interleukin-32 gamma specific monoclonal antibody and developing IL-32 specific ELISA. Hybridoma.

[190] Seth P., Yeowell H.N. (2010). Fox-2 protein regulates the alternative splicing of scleroderma-associated lysyl hydroxylase 2 messenger RNA. Arthritis Rheum..

[191] Grisar J., Munk M., Steiner C.W., Amoyo-Minar L., Tohidast-Akrad M., Zenz P., Steiner G., Smolen J.S. (2012). Expression patterns of CD44 and CD44 splice variants in patients with rheumatoid arthritis. Clin. Exp. Rheumatol..

[192] Lamana A., Ortiz A.M., Alvaro-Gracia J.M., Díaz-Sánchez B., Novalbos J., García- Vicuña R., González-Alvaro I. (2010). Characterization of serum interleukin-15 in healthy volunteers and patients with early arthritis to assess its potential use as a biomarker. Eur. Cytokine Netw..

[193] Friese M.A., Hellwage J., Jokiranta T.S., Meri S., Müller-Quernheim H.J., Peter H.H., Eibel H., Zipfel P.F. (2000). Different regulation of factor H and FHL-1/reconectin by inflammatory mediators and expression of the two proteins in rheumatoid arthritis (RA). Clin. Exp. Immunol..

[194] Jin P., Zhang J., Sumariwalla P.F., Ni I., Jorgensen B., Crawford D., Phillips S., Feldmann M., Shepard H.M., Paleolog E.M. (2008). Novel splice variants derived from the receptor tyrosine kinase superfamily are potential therapeutics for rheumatoid arthritis. Arthritis Res. Ther..

[195] Zikherman J., Weiss A. (2008). Alternative splicing of CD45: The tip of the iceberg. Immunity.

[196] Dahal L.N., Basu N., Youssef H., Khanolkar R.C., Barker R.N., Erwig L.P., Ward F.J. (2016). Immunoregulatory soluble CTLA-4 modifies effector T-cell responses in systemic lupus erythematosus. Arthritis Res. Ther..

[197] Hirata T., Usui T., Kobayashi S., Mimori T. (2015). A novel splice variant of human L-selectin encodes a soluble molecule that is elevated in serum of patients with rheumatic diseases. Biochem. Biophys. Res. Commun..

[198] Salter D.M., Godolphin J.L., Gourlay M.S., Lawson M.F., Hughes D.E., Dunne E. (1996). Analysis of human articular chondrocyte CD44 isoform expression and function in health and disease. J. Pathol..

[199] Takahashi A., Kuroki K., Okabe Y., Kasai Y., Matsumoto N., Yamada C., Takai T., Ose T., Kon S., Matsuda T. (2016). The immunosuppressive effect of domain- deleted dimer of HLA-G2 isoform in collagen-induced arthritis mice. Hum. Immunol..

[200] Korniejewska A., McKnight A.J., Johnson Z., Watson M.L., Ward S.G. (2011). Expression and agonist responsiveness of CXCR3 variants in human T lymphocytes. Immunology.

[201] De Arras L., Laws R., Leach S.M., Pontis K., Freedman J.H., Schwartz D.A., Alper S. (2014). Comparative genomics RNAi screen identifies Eftud2 as a novel regulator of innate immunity. Genetics.

[202] Nascimento A., Bruels C.C., Donkervoort S., Foley A.R., Codina A., Milisenda J.C., Estrella E.A., Li C., Pijuan J., Draper I. (2023). Variants in DTNA cause a mild, dominantly inherited muscular dystrophy. Acta Neuropathol..

[203] Kim J.Y., Yoon H.K., Song S.T., Park S.R., Shim S.C. (2017). Expression of activation- induced cytidine deaminase splicing variants in patients with ankylosing spondylitis. Autoimmunity.

[204] Jones S.A., Horiuchi S., Topley N., Yamamoto N., Fuller G.M. (2001). The soluble interleukin 6 receptor: Mechanisms of production and implications in disease. FASEB J..

[205] Sender L.Y., Gibbert K., Suezer Y., Radeke H.H., Kalinke U., Waibler Z. (2010). CD40 ligand-triggered human dendritic cells mount interleukin-23 responses that are further enhanced by danger signals. Mol. Immunol..

[206] Aigner T., Zien A., Hanisch D., Zimmer R. (2003). Gene expression in chondrocytes assessed with use of microarrays. J. Bone Jt. Surg. Am..

[207] Sun W., Xiao X., Li S., Jia X., Zhang Q. (2020). A novel deep intronic COL2A1 mutation in a family with early-onset high myopia/ocular-only Stickler syndrome. Ophthalmic Physiol. Opt..

[208] Lainez B., Fernandez-Real J.M., Romero X., Esplugues E., Cañete J.D., Ricart W., Engel P. (2004). Identification and characterization of a novel spliced variant that encodes human soluble tumor necrosis factor receptor 2. Int. Immunol..

[209] Schoonheim P.J., Chatzopoulou A., Schaaf M.J. (2010). The zebrafish as an in vivo model system for glucocorticoid resistance. Steroids.

[210] Badot V., Durez P., Van den Eynde B.J., Nzeusseu-Toukap A., Houssiau F.A., Lauwerys B.R. (2011). Rheumatoid arthritis synovial fibroblasts produce a soluble form of the interleukin-7 receptor in response to pro-inflammatory cytokines. J. Cell. Mol. Med..

[211] Scanzello C.R., Markova D.Z., Chee A., Xiu Y., Adams S.L., Anderson G., Zgonis M., Qin L., An H.S., Zhang Y. (2015). Fibronectin splice variation in human knee cartilage, meniscus and synovial membrane: Observations in osteoarthritic knee. J. Orthop. Res..

[212] Zwicky R., Müntener K., Goldring M.B., Baici A. (2002). Cathepsin B expression and down-regulation by gene silencing and antisense DNA in human chondrocytes. Biochem. J..

[213] Feng D., Stone R.C., Eloranta M.L., Sangster-Guity N., Nordmark G., Sigurdsson S., Wang C., Alm G., Syvänen A.C., Rönnblom L. (2010). Genetic variants and disease- associated factors contribute to enhanced interferon regulatory factor 5 expression in blood cells of patients with systemic lupus erythematosus. Arthritis Rheum..

[214] Ni Choileain S., Weyand N.J., Neumann C., Thomas J., So M., Astier A.L. (2011). The dynamic processing of CD46 intracellular domains provides a molecular rheostat for T cell activation. PLoS ONE.

[215] Nambiar M.P., Enyedy E.J., Warke V.G., Krishnan S., Dennis G., Wong H.K., Kammer G.M., Tsokos G.C. (2001). T cell signaling abnormalities in systemic lupus erythematosus are associated with increased mutations/polymorphisms and splice variants of T cell receptor zeta chain messenger RNA. Arthritis Rheum..

[216] Ahmed S., Marotte H., Kwan K., Ruth J.H., Campbell P.L., Rabquer B.J., Pakozdi A., Koch A.E. (2008). Epigallocatechin-3-gallate inhibits IL-6 synthesis and suppresses transsignaling by enhancing soluble gp130 production. Proc. Natl. Acad. Sci. USA.

[217] Bestall S.M., Hulse R.P., Blackley Z., Swift M., Ved N., Paton K., Beazley-Long N., Bates D.O., Donaldson L.F. (2018). Sensory neuronal sensitisation occurs through HMGB-1-RAGE and TRPV1 in high-glucose conditions. J. Cell Sci..

[218] Bachmann M., Hilker M., Grölz D., Tellmann G., Hake U., Kater L., de Wilde P., Tröster H. (1996). Different La/SS-B mRNA isoforms are expressed in salivary gland tissue of patients with primary Sjögren’s syndrome. J. Autoimmun..

[219] Jéru I., Papin S., L’hoste S., Duquesnoy P., Cazeneuve C., Camonis J., Amselem S. (2005). Interaction of pyrin with 14.3.3 in an isoform-specific and phosphorylation- dependent manner regulates its translocation to the nucleus. Arthritis Rheum..

[220] Giuliani A.L., Colognesi D., Ricco T., Roncato C., Capece M., Amoroso F., Wang Q.G., De Marchi E., Gartland A., Di Virgilio F. (2014). Trophic activity of human P2X7 receptor isoforms A and B in osteosarcoma. PLoS ONE.

[221] Rafael M.S., Cavaco S., Viegas C.S., Santos S., Ramos A., Willems B.A., Herfs M., Theuwissen E., Vermeer C., Simes D.C. (2014). Insights into the association of Gla-rich protein and osteoarthritis, novel splice variants and γ-carboxylation status. Mol. Nutr. Food Res..

[222] Rousseau J.C., Sandell L.J., Delmas P.D., Garnero P. (2004). Development and clinical application in arthritis of a new immunoassay for serum type IIA procollagen NH2 propeptide. Methods Mol. Med..

[223] Peffers M.J., Goljanek-Whysall K., Collins J., Fang Y., Rushton M., Loughlin J., Proctor C., Clegg P.D. (2016). Decoding the Regulatory Landscape of Ageing in Musculoskeletal Engineered Tissues Using Genome-Wide DNA Methylation and RNASeq. PLoS ONE.

[224] Zabeau L., Jensen C.J., Seeuws S., Venken K., Verhee A., Catteeuw D., van Loo G., Chen H., Walder K., Hollis J. (2015). Leptin’s metabolic and immune functions can be uncoupled at the ligand/receptor interaction level. Cell. Mol. Life Sci..

[225] Schwager K., Villa A., Rösli C., Neri D., Rösli-Khabas M., Moser G. (2011). A comparative immunofluorescence analysis of three clinical-stage antibodies in head and neck cancer. Head. Neck Oncol..

[226] Polgár A., Brózik M., Tóth S., Holub M., Hegyi K., Kádár A., Hodinka L., Falus A. (2000). Soluble interleukin-6 receptor in plasma and in lymphocyte culture supernatants of healthy individuals and patients with systemic lupus erythematosus and rheumatoid arthritis. Med. Sci. Monit..

[227] Misener V.L., Hui C., Malapitan I.A., Ittel M.E., Joyner A.L., Jongstra J. (1994). Expression of mouse LSP1/S37 isoforms. S37 is expressed in embryonic mesenchymal cells. J. Cell Sci..

[228] Kriegsmann J., Berndt A., Hansen T., Borsi L., Zardi L., Bräuer R., Petrow P.K., Otto M., Kirkpatrick C.J., Gay S. (2004). Expression of fibronectin splice variants and oncofetal glycosylated fibronectin in the synovial membranes of patients with rheumatoid arthritis and osteoarthritis. Rheumatol. Int..

[229] Malik N.M., Jin P., Raatz Y., Sumariwalla P.F., Kiriakidis S., Shepard M., Feldmann M., Paleolog E.M. (2010). Regulation of the angiopoietin-Tie ligand-receptor system with a novel splice variant of Tie1 reduces the severity of murine arthritis. Rheumatology.

[230] Madden J., Shearman C.P., Dunn R.L., Dastur N.D., Tan R.M., Nash G.B., Rainger G.E., Brunner A., Calder P.C., Grimble R.F. (2009). Altered monocyte CD44 expression in peripheral arterial disease is corrected by fish oil supplementation. Nutr. Metab. Cardiovasc. Dis..

[231] Ebe H., Matsumoto I., Kawaguchi H., Kurata I., Tanaka Y., Inoue A., Kondo Y., Tsuboi H., Sumida T. (2018). Clinical and functional significance of STEAP4-splice variant in CD14^+^ monocytes in patients with rheumatoid arthritis. Clin. Exp. Immunol..

[232] Wu J., Edberg J.C., Gibson A.W., Tsao B., Kimberly R.P. (1999). Single-nucleotide polymorphisms of T cell receptor zeta chain in patients with systemic lupus erythematosus. Arthritis Rheum..

[233] Suzuki Y., Ohya S., Yamamura H., Giles W.R., Imaizumi Y. (2016). A New Splice Variant of Large Conductance Ca^2+^-activated K^+^ (BK) Channel α Subunit Alters Human Chondrocyte Function. J. Biol. Chem..

[234] Du M., Roy K.M., Zhong L., Shen Z., Meyers H.E., Nichols R.C. (2006). VEGF gene expression is regulated post-transcriptionally in macrophages. FEBS J..

[235] Friese M.A., Hellwage J., Jokiranta T.S., Meri S., Peter H.H., Eibel H., Zipfel P.F. (1999). FHL-1/reconectin and factor H: Two human complement regulators which are encoded by the same gene are differently expressed and regulated. Mol. Immunol..

[236] Baici A., Lang A., Zwicky R., Müntener K. (2005). Cathepsin B in osteoarthritis: Uncontrolled proteolysis in the wrong place. Semin. Arthritis Rheum..

[237] AlFadhli S., Nizam R. (2014). Differential expression of alternative splice variants of CTLA4 in Kuwaiti autoimmune disease patients. Gene.

[238] Bauer S., Jendro M.C., Wadle A., Kleber S., Stenner F., Dinser R., Reich A., Faccin E., Gödde S., Dinges H. (2006). Fibroblast activation protein is expressed by rheumatoid myofibroblast-like synoviocytes. Arthritis Res. Ther..

[239] Horiuchi S., Ampofo W., Koyanagi Y., Yamashita A., Waki M., Matsumoto A., Yamamoto M., Yamamoto N. (1998). High-level production of alternatively spliced soluble interleukin-6 receptor in serum of patients with adult T-cell leukaemia/HTLV-I- associated myelopathy. Immunology.

[240] Muller I.B., Lin M., Lems W.F., Ter Wee M.M., Wojtuszkiewicz A., Nurmohamed M.T., Cloos J., Assaraf Y.G., Jansen G., de Jonge R. (2021). Association of altered folylpolyglutamate synthetase pre-mRNA splicing with methotrexate unresponsiveness in early rheumatoid arthritis. Rheumatology.

[241] Chiba T., Miyashita K., Sugoh T., Warita T., Inoko H., Kimura M., Sato T. (2011). IκBL, a novel member of the nuclear IκB family, inhibits inflammatory cytokine expression. FEBS Lett..

[242] Hulse R.P., Beazley-Long N., Ved N., Bestall S.M., Riaz H., Singhal P., Ballmer Hofer K., Harper S.J., Bates D.O., Donaldson L.F. (2015). Vascular endothelial growth factor-A165b prevents diabetic neuropathic pain and sensory neuronal degeneration. Clin. Sci..

[243] Punwani D., Wang H., Chan A.Y., Cowan M.J., Mallott J., Sunderam U., Mollenauer M., Srinivasan R., Brenner S.E., Mulder A. (2015). Combined immunodeficiency due to MALT1 mutations, treated by hematopoietic cell transplantation. J. Clin. Immunol..

[244] Lemaire R., Prasad J., Kashima T., Gustafson J., Manley J.L., Lafyatis R. (2002). Stability of a PKCI-1-related mRNA is controlled by the splicing factor ASF/SF2: A novel function for SR proteins. Genes Dev..

[245] Weisbart R.H., Chan G., Li E., Farmani N., Heinze E., Rubell A., Nishimura R.N., Colburn K. (2013). BRAF splice variants in rheumatoid arthritis synovial fibroblasts activate MAPK through CRAF. Mol. Immunol..

[246] Pedretti M., Rancic Z., Soltermann A., Herzog B.A., Schliemann C., Lachat M., Neri D., Kaufmann P.A. (2010). Comparative immunohistochemical staining of atherosclerotic plaques using F16, F8 and LThree clinical-grade fully human antibodies. Atherosclerosis.

[247] Cai L., Brophy R.H., Tycksen E.D., Duan X., Nunley R.M., Rai M.F. (2019). Distinct expression pattern of periostin splice variants in chondrocytes and ligament progenitor cells. FASEB J..

[248] Boyle D.L., Shi Y., Gay S., Firestein G.S. (2000). Regulation of CS1 fibronectin expression and function by IL-1 in endothelial cells. Cell. Immunol..

[249] Schwager K., Kaspar M., Bootz F., Marcolongo R., Paresce E., Neri D., Trachsel E. (2009). Preclinical characterization of DEKAVIL (F8-IL10), a novel clinical-stage immunocytokine which inhibits the progression of collagen-induced arthritis. Arthritis Res. Ther..

[250] Kono M., Kurita T., Yasuda S., Kono M., Fujieda Y., Bohgaki T., Katsuyama T., Tsokos G.C., Moulton V.R., Atsumi T. (2018). Decreased Expression of Serine/Arginine-Rich Splicing Factor 1 in T Cells From Patients With Active Systemic Lupus Erythematosus Accounts for Reduced Expression of RasGRP1 and DNA Methyltransferase 1. Arthritis Rheumatol..

[251] AlFadhli S. (2013). Overexpression and secretion of the soluble CTLA-4 splice variant in various autoimmune diseases and in cases with overlapping autoimmunity. Genet. Test. Mol. Biomark..

[252] Oparina N.Y., Delgado-Vega A.M., Martinez-Bueno M., Magro-Checa C., Fernández C., Castro R.O., Pons-Estel B.A., D’Alfonso S., Sebastiani G.D., Witte T. (2015). PXK locus in systemic lupus erythematosus: Fine mapping and functional analysis reveals novel susceptibility gene ABHD6. Ann. Rheum. Dis..

[253] Wei G., Almeida M., Pintacuda G., Coker H., Bowness J.S., Ule J., Brockdorff N. (2021). Acute depletion of METTL3 implicates *N*^6^-methyladenosine in alternative intron/exon inclusion in the nascent transcriptome. Genome Res..

[254] Thomas H., Beck K., Adamczyk M., Aeschlimann P., Langley M., Oita R.C., Thiebach L., Hils M., Aeschlimann D. (2013). Transglutaminase 6: A protein associated with central nervous system development and motor function. Amino Acids.

[255] Mamegano K., Kuroki K., Miyashita R., Kusaoi M., Kobayashi S., Matsuta K., Maenaka K., Colonna M., Ozaki S., Hashimoto H. (2008). Association of LILRA2 (ILT1, LIR7) splice site polymorphism with systemic lupus erythematosus and microscopic polyangiitis. Genes Immun..

[256] Terkeltaub R., Lotz M., Johnson K., Deng D., Hashimoto S., Goldring M.B., Burton D., Deftos L.J. (1998). Parathyroid hormone-related proteins is abundant in osteoarthritic cartilage, and the parathyroid hormone-related protein 1-173 isoform is selectively induced by transforming growth factor beta in articular chondrocytes and suppresses generation of extracellular inorganic pyrophosphate. Arthritis Rheum..

[257] Reichenbach G., Starzinski-Powitz A., Doll M., Hrgovic I., Valesky E.M., Kippenberger S., Bernd A., Kaufmann R., Meissner M. (2012). Ligand activation of peroxisome proliferator-activated receptor delta suppresses cathepsin B expression in human endothelial cells in a posttranslational manner. Exp. Dermatol..

[258] Chang H.H., Tseng W., Cui J., Costenbader K., Ho I.C. (2014). Altered expression of protein tyrosine phosphatase, non-receptor type 22 isoforms in systemic lupus erythematosus. Arthritis Res. Ther..

[259] Wibulswas A., Croft D., Pitsillides A.A., Bacarese-Hamilton I., McIntyre P., Genot E., Kramer I.M. (2002). Influence of epitopes CD44v3 and CD44v6 in the invasive behavior of fibroblast-like synoviocytes derived from rheumatoid arthritic joints. Arthritis Rheum..

[260] Carrea A., Preisegger M.A., Velasco Zamora J., Dewey R.A. (2021). The mRNA levels of TGF-β Type II receptor splice variants in monocytes are associated with disease activity in patients with rheumatoid arthritis. Clin. Exp. Rheumatol..

[261] Raben N., Nichols R.C., Martiniuk F., Plotz P.H. (1996). A model of mRNA splicing in adult lysosomal storage disease (glycogenosis type II). Hum. Mol. Genet..

[262] Peake N.J., Khawaja K., Myers A., Nowell M.A., Jones S.A., Rowan A.D., Cawston T.E., Foster H.E. (2006). Interleukin-6 signalling in juvenile idiopathic arthritis is limited by proteolytically cleaved soluble interleukin-6 receptor. Rheumatology.

[263] Claudepierre P., Allanore Y., Belec L., Larget-Piet B., Zardi L., Chevalier X. (1999). Increased Ed-B fibronectin plasma levels in spondyloarthropathies: Comparison with rheumatoid arthritis patients and a healthy population. Rheumatology.

[264] Diaz-Gallo L.M., Martin J. (2012). PTPN22 splice forms: A new role in rheumatoid arthritis. Genome Med..

[265] Bäckdahl L., Ekman D., Jagodic M., Olsson T., Holmdahl R. (2014). Identification of candidate risk gene variations by whole-genome sequence analysis of four rat strains commonly used in inflammation research. BMC Genom..

[266] Ala-Kokko L., Prockop D.J. (1990). Completion of the intron-exon structure of the gene for human type II procollagen (COL2A1): Variations in the nucleotide sequences of the alleles from three chromosomes. Genomics.

[267] Kojo S., Tsutsumi A., Goto D., Sumida T. (2003). Low expression levels of soluble CD1d gene in patients with rheumatoid arthritis. J. Rheumatol..

[268] Fattorusso R., Pellecchia M., Viti F., Neri P., Neri D., Wüthrich K. (1999). NMR structure of the human oncofoetal fibronectin ED-B domain, a specific marker for angiogenesis. Structure.

[269] Valas S., Rolland M., Perrin C., Perrin G., Mamoun R.Z. (2008). Characterization of a new 5’ splice site within the caprine arthritis encephalitis virus genome: Evidence for a novel auxiliary protein. Retrovirology.

[270] Carsons S. (2001). Extra domain-positive fibronectins in arthritis: Wolf in sheep’s clothing?. Rheumatology.

[271] Kim I.G., McBride O.W., Wang M., Kim S.Y., Idler W.W., Steinert P.M. (1992). Structure and organization of the human transglutaminase 1 gene. J. Biol. Chem..

[272] Dickie L.J., Aziz A.M., Savic S., Lucherini O.M., Cantarini L., Geiler J., Wong C.H., Coughlan R., Lane T., Lachmann H.J. (2012). Involvement of X-box binding protein 1 and reactive oxygen species pathways in the pathogenesis of tumour necrosis factor receptor-associated periodic syndrome. Ann. Rheum. Dis..

[273] Del Galdo F., Maul G.G., Jiménez S.A., Artlett C.M. (2006). Expression of allograft inflammatory factor 1 in tissues from patients with systemic sclerosis and in vitro differential expression of its isoforms in response to transforming growth factor beta. Arthritis Rheum..

[274] Guo S., Zhu Q., Jiang T., Wang R., Shen Y., Zhu X., Wang Y., Bai F., Ding Q., Zhou X. (2017). Genome-wide DNA methylation patterns in CD4+ T cells from Chinese Han patients with rheumatoid arthritis. Mod. Rheumatol..

[275] Faggian J., Fosang A.J., Zieba M., Wallace M.J., Hooper S.B. (2007). Changes in versican and chondroitin sulfate proteoglycans during structural development of the lung. Am. J. Physiol. Regul. Integr. Comp. Physiol..

[276] Gill R.B., Day A., Barstow A., Liu H., Zaman G., Dhoot G.K. (2011). Sulf2 gene is alternatively spliced in mammalian developing and tumour tissues with functional implications. Biochem. Biophys. Res. Commun..

[277] Croft D.R., Dall P., Davies D., Jackson D.G., McIntyre P., Kramer I.M. (1997). Complex CD44 splicing combinations in synovial fibroblasts from arthritic joints. Eur. J. Immunol..

[278] Michel J., Langstein J., Hofstädter F., Schwarz H. (1998). A soluble form of CD137 (ILA/4-1BB), a member of the TNF receptor family, is released by activated lymphocytes and is detectable in sera of patients with rheumatoid arthritis. Eur. J. Immunol..

[279] Wen F., Ellingson S.M., Kyogoku C., Peterson E.J., Gaffney P.M. (2011). Exon 6 variants carried on systemic lupus erythematosus (SLE) risk haplotypes modulate IRF5 function. Autoimmunity.

[280] Li H., Reksten T.R., Ice J.A., Kelly J.A., Adrianto I., Rasmussen A., Wang S., He B., Grundahl K.M., Glenn S.B. (2017). Identification of a Sjögren’s syndrome susceptibility locus at OAS1 that influences isoform switching, protein expression, and responsiveness to type I interferons. PLoS Genet..

[281] Cutolo M., Picasso M., Ponassi M., Sun M.Z., Balza E. (1992). Tenascin and fibronectin distribution in human normal and pathological synovium. J. Rheumatol..

[282] Schaaf M.J., Cidlowski J.A. (2002). AUUUA motifs in the 3’UTR of human glucocorticoid receptor alpha and beta mRNA destabilize mRNA and decrease receptor protein expression. Steroids.

[283] Kerna I., Kisand K., Suutre S., Murde M., Tamm A., Kumm J., Tamm A. (2014). The ADAM12 is upregulated in synovitis and postinflammatory fibrosis of the synovial membrane in patients with early radiographic osteoarthritis. Jt. Bone Spine.

[284] Rogers G.R., Markova N.G., De Laurenzi V., Rizzo W.B., Compton J.G. (1997). Genomic organization and expression of the human fatty aldehyde dehydrogenase gene (FALDH). Genomics.

[285] Tsukada Y., Ichikawa H., Chai Z., Lai F.P., Dunster K., Sentry J.W., Toh B.H. (2000). Novel variant of p230 trans-Golgi network protein identified by serum from Sjögren’s syndrome patient. Eur. J. Cell Biol..

[286] Nedvetzki S., Walmsley M., Alpert E., Williams R.O., Feldmann M., Naor D. (1999). CD44 involvement in experimental collagen-induced arthritis (CIA). J. Autoimmun..

[287] Mokuda S., Miyazaki T., Ito Y., Yamasaki S., Inoue H., Guo Y., Kong W.S., Kanno M., Takasugi K., Sugiyama E. (2015). The proto-oncogene survivin splice variant 2B is induced by PDGF and leads to cell proliferation in rheumatoid arthritis fibroblast-like synoviocytes. Sci. Rep..

[288] Ohkubo T., Takei M., Mitamura K., Horie T., Fujiwara S., Shimizu K., Ryu J., Shiraiwa H., Sawada S. (2001). Increased soluble CD4 molecules and the role of soluble CD4 production in patients with rheumatoid arthritis. J. Int. Med. Res..

[289] Vollmer S., Vater A., Licha K., Gemeinhardt I., Gemeinhardt O., Voigt J., Ebert B., Schnorr J., Taupitz M., Macdonald R. (2009). Extra domain B fibronectin as a target for near-infrared fluorescence imaging of rheumatoid arthritis affected joints in vivo. Mol. Imaging.

[290] Hikichi Y., Yoshimura K., Takigawa M. (2009). All-trans retinoic acid-induced ADAM28 degrades proteoglycans in human chondrocytes. Biochem. Biophys. Res. Commun..

[291] Roescher N., Vosters J.L., Alsaleh G., Dreyfus P., Jacques S., Chiocchia G., Sibilia J., Tak P.P., Chiorini J.A., Mariette X. (2014). Targeting the splicing of mRNA in autoimmune diseases: BAFF inhibition in Sjögren’s syndrome as a proof of concept. Mol. Ther..

[292] Kapsogeorgou E.K., Manoussakis M.N. (2010). Salivary gland epithelial cells (SGEC): Carriers of exquisite B7-2 (CD86) costimulatory molecules. J. Autoimmun..

[293] Fay J., Varoga D., Wruck C.J., Kurz B., Goldring M.B., Pufe T. (2006). Reactive oxygen species induce expression of vascular endothelial growth factor in chondrocytes and human articular cartilage explants. Arthritis Res. Ther..

[294] Jiang H., Knudson C.B., Knudson W. (2001). Antisense inhibition of CD44 tailless splice variant in human articular chondrocytes promotes hyaluronan internalization. Arthritis Rheum..

[295] Tolboom T.C., Huidekoper A.L., Kramer I.M., Pieterman E., Toes R.E., Huizinga T.W. (2004). Correlation between expression of CD44 splice variant v8-v9 and invasiveness of fibroblast-like synoviocytes in an in vitro system. Clin. Exp. Rheumatol..

[296] Peters J.H., Carsons S., Kalunian K., McDougall S., Yoshida M., Ko F., van der Vliet-Hristova M., Hahn T.J. (2001). Preferential recognition of a fragment species of osteoarthritic synovial fluid fibronectin by antibodies to the alternatively spliced EIIIA segment. Arthritis Rheum..

[297] Niarakis A., Giannopoulou E., Ravazoula P., Panagiotopoulos E., Zarkadis I.K., Aletras A.J. (2013). Detection of a latent soluble form of membrane type 1 matrix metalloprotease bound with tissue inhibitor of matrix metalloproteinases-2 in periprosthetic tissues and fluids from loose arthroplasty endoprostheses. FEBS J..

[298] Hirayasu K., Ohashi J., Kashiwase K., Takanashi M., Satake M., Tokunaga K., Yabe T. (2006). Long-term persistence of both functional and non-functional alleles at the leukocyte immunoglobulin-like receptor A3 (LILRA3) locus suggests balancing selection. Hum. Genet..

[299] Yousaf N., Low W.Y., Onipinla A., Mein C., Caulfield M., Munroe P.B., Chernajovsky Y. (2015). Differences between disease-associated endoplasmic reticulum aminopeptidase 1 (ERAP1) isoforms in cellular expression, interactions with tumour necrosis factor receptor 1 (TNF-R1) and regulation by cytokines. Clin. Exp. Immunol..

[300] Hasegawa M., Nakoshi Y., Muraki M., Sudo A., Kinoshita N., Yoshida T., Uchida A. (2007). Expression of large tenascin-C splice variants in synovial fluid of patients with rheumatoid arthritis. J. Orthop. Res..

[301] Wainwright S.D., Bondeson J., Hughes C.E. (2006). An alternative spliced transcript of ADAMTS4 is present in human synovium from OA patients. Matrix Biol..

[302] Buyon J.P., Tseng C.E., Di Donato F., Rashbaum W., Morris A., Chan E.K. (1997). Cardiac expression of 52beta, an alternative transcript of the congenital heart block- associated 52-kd SS-A/Ro autoantigen, is maximal during fetal development. Arthritis Rheum..

[303] Sciore P., Frank C.B., Hart D.A. (1998). Identification of sex hormone receptors in human and rabbit ligaments of the knee by reverse transcription-polymerase chain reaction: Evidence that receptors are present in tissue from both male and female subjects. J. Orthop. Res..

[304] Greetham D., Ellis C.D., Mewar D., Fearon U., an Ultaigh S.N., Veale D.J., Guesdon F., Wilson A.G. (2007). Functional characterization of NF-kappaB inhibitor-like protein 1 (NFkappaBIL1), a candidate susceptibility gene for rheumatoid arthritis. Hum. Mol. Genet..

[305] Hoornaert K.P., Vereecke I., Dewinter C., Rosenberg T., Beemer F.A., Leroy J.G., Bendix L., Björck E., Bonduelle M., Boute O. (2010). Stickler syndrome caused by COL2A1 mutations: Genotype- phenotype correlation in a series of 100 patients. Eur. J. Hum. Genet..

[306] Alvarez-Errico D., Yamashita Y., Suzuki R., Odom S., Furumoto Y., Yamashita T., Rivera J. (2010). Functional analysis of Lyn kinase A and B isoforms reveals redundant and distinct roles in Fc epsilon RI-dependent mast cell activation. J. Immunol..

[307] Kalinski H., Yaniv A., Mashiah P., Miki T., Tronick S.R., Gazit A. (1991). rev-like transcripts of caprine arthritis encephalitis virus. Virology.

[308] Derijk R.H., Schaaf M.J., Turner G., Datson N.A., Vreugdenhil E., Cidlowski J., de Kloet E.R., Emery P., Sternberg E.M., Detera-Wadleigh S.D. (2001). A human glucocorticoid receptor gene variant that increases the stability of the glucocorticoid receptor beta-isoform mRNA is associated with rheumatoid arthritis. J. Rheumatol..

[309] Shiozawa K., Hino K., Shiozawa S. (2001). Alternatively spliced EDA-containing fibronectin in synovial fluid as a predictor of rheumatoid joint destruction. Rheumatology.

[310] Ray B.K., Murphy R., Ray P., Ray A. (2002). SAF-2, a splice variant of SAF-1, acts as a negative regulator of transcription. J. Biol. Chem..

[311] Lemaire R., Flipo R.M., Migaud H., Fontaine C., Huet G., Dacquembronne E., Lafyatis R. (1997). Alternative splicing of the 5’ region of cathepsin B pre-messenger RNA in rheumatoid synovial tissue. Arthritis Rheum..

[312] Liu J.H., Wei S., Lamy T., Li Y., Epling-Burnette P.K., Djeu J.Y., Loughran T.P. (2002). Blockade of Fas-dependent apoptosis by soluble Fas in LGL leukemia. Blood.

[313] Dugan J., Griffiths E., Snow P., Rosenzweig H., Lee E., Brown B., Carr D.W., Rose C., Rosenbaum J., Davey M.P. (2015). Blau syndrome-associated Nod2 mutation alters expression of full-length NOD2 and limits responses to muramyl dipeptide in knock-in mice. J. Immunol..

[314] Maretzky T., Le Gall S.M., Worpenberg-Pietruk S., Eder J., Overall C.M., Huang X.Y., Poghosyan Z., Edwards D.R., Blobel C.P. (2009). Src stimulates fibroblast growth factor receptor-2 shedding by an ADAM15 splice variant linked to breast cancer. Cancer Res..

[315] Banda N.K., Mehta G., Kjaer T.R., Takahashi M., Schaack J., Morrison T.E., Thiel S., Arend W.P., Holers V.M. (2014). Essential role for the lectin pathway in collagen antibody- induced arthritis revealed through use of adenovirus programming complement inhibitor MAp44 expression. J. Immunol..

[316] Sarkissian M., Lafyatis R. (1998). Transforming growth factor-beta and platelet derived growth factor regulation of fibrillar fibronectin matrix formation by synovial fibroblasts. J. Rheumatol..

[317] Labat-Robert J., Chevalier X. (1991). Fibronectine, vieillissement et pathologies associées [Fibronectin, aging and related pathologies]. C. R. Seances Soc. Biol. Fil..

[318] Wan B., Nie H., Liu A., Feng G., He D., Xu R., Zhang Q., Dong C., Zhang J.Z. (2006). Aberrant regulation of synovial T cell activation by soluble costimulatory molecules in rheumatoid arthritis. J. Immunol..

[319] Proussakova O.V., Rabaya N.A., Moshnikova A.B., Telegina E.S., Turanov A., Nanazashvili M.G., Beletsky I.P. (2003). Oligomerization of soluble Fas antigen induces its cytotoxicity. J. Biol. Chem..

[320] Pufe T., Petersen W., Tillmann B., Mentlein R. (2001). The splice variants VEGF121 and VEGF189 of the angiogenic peptide vascular endothelial growth factor are expressed in osteoarthritic cartilage. Arthritis Rheum..

[321] Wainwright S.D., Bondeson J., Caterson B., Hughes C.E. (2013). ADAMTS-4_v1 is a splice variant of ADAMTS-4 that is expressed as a protein in human synovium and cleaves aggrecan at the interglobular domain. Arthritis Rheum..

[322] Banda N.K., Desai D., Scheinman R.I., Pihl R., Sekine H., Fujita T., Sharma V., Hansen A.G., Garred P., Thiel S. (2018). Targeting of Liver Mannan-Binding Lectin-Associated Serine Protease-3 with RNA Interference Ameliorates Disease in a Mouse Model of Rheumatoid Arthritis. Immunohorizons.

[323] Oda H., Beck D.B., Kuehn H.S., Sampaio Moura N., Hoffmann P., Ibarra M., Stoddard J., Tsai W.L., Gutierrez-Cruz G., Gadina M. (2019). Second Case of HOIP Deficiency Expands Clinical Features and Defines Inflammatory Transcriptome Regulated by LUBAC. Front. Immunol..

[324] Ryder L.R., Ryder L.P., Bartels E.M., Woetmann A., Madsen H.O., Ødum N., Danneskiold- Samsøe B., Ribel-Madsen S., Bliddal H. (2013). Differential effects of decoy receptor- and antibody-mediated tumour necrosis factor blockage on FoxP3 expression in responsive arthritis patients. APMIS.

[325] Claus R., Bittorf T., Walzel H., Brock J., Uhde R., Meiske D., Schulz U., Hobusch D., Schumacher K., Witt M. (2000). High concentration of soluble HLA-DR in the synovial fluid: Generation and significance in "rheumatoid-like" inflammatory joint diseases. Cell. Immunol..

[326] Weissbach L., Tran K., Colquhoun S.A., Champliaud M.F., Towle C.A. (1998). Detection of an interleukin-1 intracellular receptor antagonist mRNA variant. Biochem. Biophys. Res. Commun..

[327] Seperack P.K., Mercer J.A., Strobel M.C., Copeland N.G., Jenkins N.A. (1995). Retroviral sequences located within an intron of the dilute gene alter dilute expression in a tissue-specific manner. EMBO J..

[328] Andersson S.E., Svensson M.N., Erlandsson M.C., Dehlin M., Andersson K.M., Bokarewa M.I. (2012). Activation of Fms-like tyrosine kinase 3 signaling enhances survivin expression in a mouse model of rheumatoid arthritis. PLoS ONE.

[329] Richards A.J., Laidlaw M., Meredith S.P., Shankar P., Poulson A.V., Scott J.D., Snead M.P. (2007). Missense and silent mutations in COL2A1 result in Stickler syndrome but via different molecular mechanisms. Hum. Mutat..

[330] Woolard J., Wang W.Y., Bevan H.S., Qiu Y., Morbidelli L., Pritchard-Jones R.O., Cui T.G., Sugiono M., Waine E., Perrin R. (2004). VEGF165b, an inhibitory vascular endothelial growth factor splice variant: Mechanism of action, in vivo effect on angiogenesis and endogenous protein expression. Cancer Res..

[331] Kaur G., Goodall J.C., Jarvis L.B., Hill Gaston J.S. (2010). Characterisation of Foxp3 splice variants in human CD4+ and CD8+ T cells—Identification of Foxp3Δ7 in human regulatory T cells. Mol. Immunol..

[332] Wibulswas A., Croft D., Bacarese-Hamilton I., McIntyre P., Genot E., Kramer I.M. (2000). The CD44v7/8 epitope as a target to restrain proliferation of fibroblast-like synoviocytes in rheumatoid arthritis. Am. J. Pathol..

[333] Nambiar M.P., Enyedy E.J., Fisher C.U., Krishnan S., Warke V.G., Gilliland W.R., Oglesby R.J., Tsokos G.C. (2002). Abnormal expression of various molecular forms and distribution of T cell receptor zeta chain in patients with systemic lupus erythematosus. Arthritis Rheum..

[334] Aksentijevich I., Galon J., Soares M., Mansfield E., Hull K., Oh H.H., Goldbach- Mansky R., Dean J., Athreya B., Reginato A.J. (2001). The tumor-necrosis-factor receptor-associated periodic syndrome: New mutations in TNFRSF1A, ancestral origins, genotype-phenotype studies, and evidence for further genetic heterogeneity of periodic fevers. Am. J. Hum. Genet..

[335] Miyashita K., Sakashita E., Miyamoto K., Tokita M., Komai T. (1998). Development of the selective adsorbent for EDA containing fibronectin using heparin immobilized cellulose. Int. J. Biol. Macromol..

[336] Elices M.J., Tsai V., Strahl D., Goel A.S., Tollefson V., Arrhenius T., Wayner E.A., Gaeta F.C., Fikes J.D., Firestein G.S. (1994). Expression and functional significance of alternatively spliced CS1 fibronectin in rheumatoid arthritis microvasculature. J. Clin. Investig..

[337] Pufe T., Petersen W., Tillmann B., Mentlein R. (2001). Splice variants VEGF121 and VEGF165 of the angiogenic peptide vascular endothelial cell growth factor are expressed in the synovial tissue of patients with rheumatoid arthritis. J. Rheumatol..

[338] Sherman J.B., Raben N., Nicastri C., Argov Z., Nakajima H., Adams E.M., Eng C.M., Cowan T.M., Plotz P.H. (1994). Common mutations in the phosphofructokinase-M gene in Ashkenazi Jewish patients with glycogenesis VII--and their population frequency. Am. J. Hum. Genet..

[339] Del Galdo F., Jiménez S.A. (2007). T cells expressing allograft inflammatory factor 1 display increased chemotaxis and induce a profibrotic phenotype in normal fibroblasts in vitro. Arthritis Rheum..

[340] Horie R., Ito K., Tatewaki M., Nagai M., Aizawa S., Higashihara M., Ishida T., Inoue J., Takizawa H., Watanabe T. (1996). A variant CD30 protein lacking extracellular and transmembrane domains is induced in HL-60 by tetradecanoylphorbol acetate and is expressed in alveolar macrophages. Blood.

[341] Diaz A., Hu C., Kastner D.L., Schaner P., Reginato A.M., Richards N., Gumucio D.L. (2004). Lipopolysaccharide-induced expression of multiple alternatively spliced MEFV transcripts in human synovial fibroblasts: A prominent splice isoform lacks the C-terminal domain that is highly mutated in familial Mediterranean fever. Arthritis Rheum..

[342] Chan E.K., Di Donato F., Hamel J.C., Tseng C.E., Buyon J.P. (1995). 52-kD SS-A/Ro: Genomic structure and identification of an alternatively spliced transcript encoding a novel leucine zipper-minus autoantigen expressed in fetal and adult heart. J. Exp. Med..

[343] Kozyrev S.V., Lewén S., Reddy P.M., Pons-Estel B., Witte T., Junker P., Laustrup H., Gutiérrez C., Argentine Collaborative Group, German Collaborative Group (2007). Structural insertion/deletion variation in IRF5 is associated with a risk haplotype and defines the precise IRF5 isoforms expressed in systemic lupus erythematosus. Arthritis Rheum..

[344] Hospach T., Lohse P., Heilbronner H., Dannecker G.E., Lohse P. (2005). Pseudodominant inheritance of the hyperimmunoglobulinemia D with periodic fever syndrome in a mother and her two monozygotic twins. Arthritis Rheum..

[345] Raben N., Sherman J., Miller F., Mena H., Plotz P. (1993). A 5’ splice junction mutation leading to exon deletion in an Ashkenazic Jewish family with phosphofructokinase deficiency (Tarui disease). J. Biol. Chem..

[346] Jackson J.R., Minton J.A., Ho M.L., Wei N., Winkler J.D. (1997). Expression of vascular endothelial growth factor in synovial fibroblasts is induced by hypoxia and interleukin 1beta. J. Rheumatol..

[347] Bär K.J., Natura G., Telleria-Diaz A., Teschner P., Vogel R., Vasquez E., Schaibl H.G., Ebersberger A. (2004). Changes in the effect of spinal prostaglandin E2 during inflammation: Prostaglandin E (EP1-EP4) receptors in spinal nociceptive processing of input from the normal or inflamed knee joint. J. Neurosci..

[348] Tröster H., Metzger T.E., Semsei I., Schwemmle M., Winterpacht A., Zabel B., Bachmann M. (1994). One gene, two transcripts: Isolation of an alternative transcript encoding for the autoantigen La/SS-B from a cDNA library of a patient with primary Sjögrens’ syndrome. J. Exp. Med..

[349] Hu S.I., Klein M., Carozza M., Rediske J., Peppard J., Qi J.S. (1999). Identification of a splice variant of neutrophil collagenase (MMP-8). FEBS Lett..

[350] Hino K., Maeda T., Sekiguchi K., Shiozawa K., Hirano H., Sakashita E., Shiozawa S. (1996). Adherence of synovial cells on EDA-containing fibronectin. Arthritis Rheum..

[351] Shevchenko Y.O., Compton J.G., Toro J.R., DiGiovanna J.J., Bale S.J. (2000). Splice-site mutation in TGM1 in congenital recessive ichthyosis in American families: Molecular, genetic, genealogic, and clinical studies. Hum. Genet..

[352] Hübener C., Mincheva A., Lichter P., Schraven B., Bruyns E. (2000). Genomic organization and chromosomal localization of the human gene encoding the T-cell receptor-interacting molecule (TRIM). Immunogenetics.

[353] Møller H.J., Ingemann-Hansen T., Poulsen J.H. (1994). The epidermal growth factor-like domain of the human cartilage large aggregating proteoglycan, aggrecan: Increased serum concentration in rheumatoid arthritis. Br. J. Rheumatol..

[354] Kaufman K.M., Kirby M.Y., McClain M.T., Harley J.B., James J.A. (2001). Lupus autoantibodies recognize the product of an alternative open reading frame of SmB/B’. Biochem. Biophys. Res. Commun..

[355] Chae J.J., Komarow H.D., Cheng J., Wood G., Raben N., Liu P.P., Kastner D.L. (2003). Targeted disruption of pyrin, the FMF protein, causes heightened sensitivity to endotoxin and a defect in macrophage apoptosis. Mol. Cell.

[356] Feyertag J., Haberhauer G., Skoumal M., Kittl E.M., Bauer K., Dunky A. (2000). Serumspiegel löslicher CD44-Isoform-Variante 5 von Patienten mit seropositiver Rheumatoid-Arthritis unter Cyclosporin-A-Therapie [Serum soluble CD44 isoform variant 5 level in patients with seropositive rheumatoid arthritis treated with cyclosporin A]. Acta Med. Austriaca.

[357] Zhang H., Phang D., Laxer R.M., Silverman E.D., Pan S., Doherty P.J. (1997). Evolution of the T cell receptor beta repertoire from synovial fluid T cells of patients with juvenile onset rheumatoid arthritis. J. Rheumatol..

[358] Cole W.G. (1997). Abnormal skeletal growth in Kniest dysplasia caused by type II collagen mutations. Clin. Orthop. Relat. Res..

[359] Gazit A., Mashiah P., Kalinski H., Gast A., Rosin-Abersfeld R., Tronick S.R., Yaniv A. (1996). Two species of Rev proteins, with distinct N termini, are expressed by caprine arthritis encephalitis virus. J. Virol..

[360] Nichols R.C., Rudolphi O., Ek B., Exelbert R., Plotz P.H., Raben N. (1996). Glycogenosis type VII (Tarui disease) in a Swedish family: Two novel mutations in muscle phosphofructokinase gene (PFK-M) resulting in intron retentions. Am. J. Hum. Genet..

[361] Hiraoka M., Saito I., Tsubota K., Sugai S., Miyasaka N. (1997). Augmented expression of CD44 splice variants in lymphoproliferative disorder of the lacrimal gland in Sjögren’s syndrome. Jpn. J. Ophthalmol..

[362] Müller-Ladner U., Kriegsmann J., Strahl D., Gay R.E., Elices M., Gay S. (1996). Messenger-RNA-Expression der alternativ gesplicten CS-1 Fibronektin-Isoformen im Rheumatoid-Arthritis (RA)-Synovium [Messenger RNA expression of alternatively spliced CS-1 fibronectin isoforms in Rheumatoid arthritis (RA) synovium]. Verh. Dtsch. Ges. Pathol..

[363] Li C., Wei P., Wang L., Wang Q., Wang H., Zhang Y. (2023). Integrated Analysis of Transcriptome Changes in Osteoarthritis: Gene Expression, Pathways and Alternative Splicing. Cartilage.

[364] Muller I.B., Lin M., Jonge R., Will N., López-Navarro B., Laken C.V., Struys E.A., Oudejans C.B.M., Assaraf Y.G., Cloos J. (2023). Methotrexate Provokes Disparate Folate Metabolism Gene Expression and Alternative Splicing in Ex Vivo Monocytes and GM-CSF- and M-CSF-Polarized Macrophages. Int. J. Mol. Sci..

