# Supplementary material for: Get Spliced: Uniting Alternative Splicing and Arthritis
Source: Int J Mol Sci. 2024 Jul 25;25(15):8123. doi: 10.3390/ijms25158123 (PMC11311815; doi:10.3390/ijms25158123)
Supplement: Supplementary file 1 [file ijms-25-08123-s001.zip › ijms-3099395-supplementary.pdf]

Supplementary SI

Table summary of alternative splicing events in arthritis. Reference number refers to the references listed in Supplementary SII.

Gene	References
ADAMTS	12, 28, 237, 257
Fibronectin	13,33,64,118, 147, 164, 182, 184, 185, 199, 204, 206, 217,225, 232, 236, 245, 271, 272, 286, 298
IL-6	15,37, 47,70, 140, 162,175, 198
IL-32gamma	16,40, 105, 125
Cathepsin B	17,24, 49, 148, 172, 193, 247
CD44	19, 30, 31,57,69, 85, 90, 127, 134, 166, 174, 195, 213, 222, 230, 231, 268, 292, 297
CD6	25, 60
PTPN22	34, 113, 194, 200
CTLA-4	54, 108, 114, 124, 132, 173, 187, 254
Survivin	59, 223, 264
Tnfar2	73, 144
IL1R	104, 262
Foxp3	123, 260, 267
COL2A1	143, 202, 241, 265
VEGF	170, 178, 229, 256, 266, 273, 282
La/SS-B	154, 284
Factor H and FHL-1 reconectin	129, 171

Supplementary SII

Total query results in pubmed: “(arthritis) AND ((alternative splicing) OR (splicing isoform))” Date: 3-5-2023

1: Ren P, Lu L, Cai S, Chen J, Lin W, Han F. Alternative Splicing: A New Cause and Potential Therapeutic Target in Autoimmune Disease. Front Immunol. 2021 Aug 17;12:713540. doi: 10.3389/fimmu.2021.713540. PMID: 34484216; PMCID: PMC8416054.

2: Midwood KS, Chiquet M, Tucker RP, Orend G. Tenascin-C at a glance. J Cell Sci. 2016 Dec 1;129(23):4321-4327. doi: 10.1242/jcs.190546. Epub 2016 Nov 10. PMID: 27875272.

3: Ibáñez-Costa A, Perez-Sanchez C, Patiño-Trives AM, Luque-Tevar M, Font P, Arias de la Rosa I, Roman-Rodriguez C, Abalos-Aguilera MC, Conde C, Gonzalez A, Pedraza-Arevalo S, Del Rio-Moreno M, Blazquez-Encinas R, Segui P, Calvo J, Ortega Castro R, Escudero-Contreras A, Barbarroja N, Aguirre MA, Castaño JP, Luque RM, Collantes-Estevez E, Lopez-Pedrera C. Splicing machinery is impaired in rheumatoid arthritis, associated with disease activity and modulated by anti-TNF therapy. Ann Rheum Dis. 2022 Jan;81(1):56-67. doi: 10.1136/annrheumdis-2021-220308. Epub 2021 Oct 8. PMID: 34625402; PMCID: PMC8762032.

4: Wan Y, Anastasakis DG, Rodriguez J, Palangat M, Gudla P, Zaki G, Tandon M, Pegoraro G, Chow CC, Hafner M, Larson DR. Dynamic imaging of nascent RNA reveals general principles of transcription dynamics and stochastic splice site selection. Cell. 2021 May 27;184(11):2878-2895.e20. doi: 10.1016/j.cell.2021.04.012. Epub 2021 May 11. PMID: 33979654; PMCID: PMC8183334.

5: Donaldson LF, Beazley-Long N. Alternative RNA splicing: contribution to pain and potential therapeutic strategy. Drug Discov Today. 2016 Nov;21(11):1787-1798. doi: 10.1016/j.drudis.2016.06.017. Epub 2016 Jun 18. PMID: 27329269; PMCID: PMC5405051.

6: Fan X, Tang L. Aberrant and alternative splicing in skeletal system disease. Gene. 2013 Oct 1;528(1):21-6. doi: 10.1016/j.gene.2013.06.027. Epub 2013 Jun 23. PMID: 23800666.

7: Aravilli RK, Vikram SL, Kohila V. The Functional Impact of Alternative Splicing and Single Nucleotide Polymorphisms in Rheumatoid Arthritis. Curr Pharm Biotechnol. 2021;22(8):1014-1029. doi: 10.2174/1389201021666201001142416. PMID: 33001009.

8: Lee Y, Wessel AW, Xu J, Reinke JG, Lee E, Kim SM, Hsu AP, Zilberman-RudenkoJ, Cao S, Enos C, Brooks SR, Deng Z, Lin B, de Jesus AA, Hupalo DN, Piotto DG, Terreri MT, Dimitriades VR, Dalgard CL, Holland SM, Goldbach-Mansky R, Siegel RM, Hanson EP. Genetically programmed alternative splicing of NEMO mediates anautoinflammatory disease phenotype. J Clin Invest. 2022 Mar 15;132(6):e128808.doi: 10.1172/JCI128808. PMID: 35289316; PMCID: PMC8920334.

9: Poirier EZ, Buck MD, Chakravarty P, Carvalho J, Frederico B, Cardoso A, Healy L, Ulferts R, Beale R, Reis e Sousa C. An isoform of Dicer protects mammalianstem cells against multiple RNA viruses. Science. 2021 Jul 9;373(6551):231-236. doi: 10.1126/science.abg2264. PMID: 34244417; PMCID: PMC7611482.

10: Katsoula G, Steinberg J, Tuerlings M, Coutinho de Almeida R, Southam L, Swift D, Meulenbelt I, Wilkinson JM, Zeggini E. A molecular map of long non-coding RNA expression, isoform switching and alternative splicing in osteoarthritis. Hum Mol Genet. 2022 Jun 22;31(12):2090-2105. doi:10.1093/hmg/ddac017. PMID: 35088088; PMCID: PMC9239745.

11: Liu Z, Wang H, Hou Y, Yang Y, Jia J, Wu J, Zuo Z, Gao T, Ren S, Bian Y, Liu S, Fu J, Sun Y, Li J, Yamamoto M, Zhang Q, Xu Y, Pi J. CNC-bZIP protein NFE2L1 regulates osteoclast differentiation in antioxidant-dependent and independent manners. Redox Biol. 2021 Nov 6;48:102180. doi: 10.1016/j.redox.2021.102180. Epub ahead of print. PMID: 34763297; PMCID: PMC8591424.

12: Rose KWJ, Taye N, Karoulias SZ, Hubmacher D. Regulation of ADAMTS Proteases.Front Mol Biosci. 2021 Jun 29;8:701959. doi: 10.3389/fmolb.2021.701959. PMID:34268335; PMCID: PMC8275829.

13: Romberger DJ. Fibronectin. Int J Biochem Cell Biol. 1997 Jul;29(7):939-43.doi: 10.1016/s1357-2725(96)00172-0. PMID: 9375374.

14: Clanchy FIL, Borghese F, Bystrom J, Balog A, Penn H, Taylor PC, Stone TW,Mageed RA, Williams RO. Disease status in human and experimental arthritis, and response to TNF blockade, is associated with MHC class II invariant chain (CD74) isoform expression. J Autoimmun. 2022 Apr;128:102810. doi:10.1016/j.jaut.2022.102810. Epub 2022 Mar 1. PMID: 35245865.

15: Lamas JR, Rodríguez-Rodríguez L, Tornero-Esteban P, Villafuertes E, Hoyas J, Abasolo L, Varadé J, Alvarez-Lafuente R, Urcelay E, Fernández-Gutiérrez B. Alternative splicing and proteolytic rupture contribute to the generation of soluble IL-6 receptors (sIL-6R) in rheumatoid arthritis. Cytokine. 2013 Mar;61(3):720-3. doi: 10.1016/j.cyto.2012.12.025. Epub 2013 Jan 29. PMID:23375120.

16: Heinhuis B, Koenders MI, van de Loo FA, Netea MG, van den Berg WB, Joosten LA. Inflammation-dependent secretion and splicing of IL-32(gamma) in rheumatoid arthritis. Proc Natl Acad Sci U S A. 2011 Mar 22;108(12):4962-7. doi:10.1073/pnas.1016005108. Epub 2011 Mar 7. PMID: 21383200; PMCID: PMC3064318.

17: Baici A, Müntener K, Willimann A, Zwicky R. Regulation of human cathepsin B by alternative mRNA splicing: homeostasis, fatal errors and cell death. Biol Chem. 2006 Aug;387(8):1017-21. doi: 10.1515/BC.2006.125. PMID: 16895470.

18: Shchetynsky K, Protsyuk D, Ronninger M, Diaz-Gallo LM, Klareskog L, Padyukov L. Gene-gene interaction and RNA splicing profiles of MAP2K4 gene in rheumatoid arthritis. Clin Immunol. 2015

May;158(1):19-28. doi: 10.1016/j.clim.2015.02.011.Epub 2015 Feb 27. PMID: 25732927.

19: Muys BR, Anastasakis DG, Claypool D, Pongor L, Li XL, Grammatikakis I, Liu M, Wang X, Prasanth KV, Aladjem MI, Lal A, Hafner M. The p53-induced RNA-binding protein ZMAT3 is a splicing regulator that inhibits the splicing of oncogenic CD44 variants in colorectal carcinoma. Genes Dev. 2021 Jan 1;35(1-2):102-116. doi: 10.1101/gad.342634.120. Epub 2020 Dec 17. PMID: 33334821; PMCID: PMC7778265.

20: Santiago B, Izquierdo E, Rueda P, Del Rey MJ, Criado G, Usategui A, Arenzana-Seisdedos F, Pablos JL. CXCL12γ isoform is expressed on endothelial and dendritic cells in rheumatoid arthritis synovium and regulates T cell activation. Arthritis Rheum. 2012 Feb;64(2):409-17. doi: 10.1002/art.33345. PMID: 21953583.

21: Cao W, He W. UL16 binding proteins. Immunobiology. 2004;209(3):283-90. doi:10.1016/j.imbio.2004.04.008. PMID: 15518340.

22: Mobasheri A, Matta C, Uzielienè I, Budd E, Martín-Vasallo P, Bernotiene E. The chondrocyte channelome: A narrative review. Joint Bone Spine. 2019 Jan;86(1):29-35. doi: 10.1016/j.jbspin.2018.01.012. Epub 2018 Feb 13. PMID: 29452304.

23: Lu S, Borst DE, Horowits R. Expression and alternative splicing of N-RAP during mouse skeletal muscle development. Cell Motil Cytoskeleton. 2008 Dec;65(12):945-54. doi: 10.1002/cm.20317. PMID: 18792955; PMCID: PMC2754055.

24: Berardi S, Lang A, Kostoulas G, Hörler D, Vilei EM, Baici A. Alternative messenger RNA splicing and enzyme forms of cathepsin B in human osteoarthritic cartilage and cultured chondrocytes. Arthritis Rheum. 2001 Aug;44(8):1819-31. doi: 10.1002/1529-0131(200108)44:8<1819::AID-ART319>3.0.CO;2-4. PMID: 11508434.

25: da Glória VG, Martins de Araújo M, Mafalda Santos A, Leal R, de Almeida SF,Carmo AM, Moreira A. T cell activation regulates CD6 alternative splicing by transcription dynamics and SRSF1. J Immunol. 2014 Jul 1;193(1):391-9. doi:10.4049/jimmunol.1400038. Epub 2014 Jun 2. PMID: 24890719.

26: Bondos SE, Geraldo Mendes G, Jons A. Context-dependent HOX transcription factor function in health and disease. Prog Mol Biol Transl Sci.2020;174:225-262. doi: 10.1016/bs.pmbts.2020.05.003. Epub 2020 Jul 6. PMID:32828467.

27: Holland DO, Gotea V, Fedkenheuer K, Jaiswal SK, Baugher C, Tan H,Fedkenheuer M, Elnitski L. Characterization and clustering of kinase isoform expression in metastatic melanoma. PLoS Comput Biol. 2022 May 13;18(5):e1010065.doi: 10.1371/journal.pcbi.1010065. PMID: 35560144; PMCID: PMC9132324.

28: Lin EA, Liu CJ. The role of ADAMTSs in arthritis. Protein Cell. 2010 Jan;1(1):33-47. doi: 10.1007/s13238-010-0002-5. Epub 2010 Feb 7. PMID: 21203996; PMCID: PMC4418016.

29: Giannopoulou EG, Elemento O, Ivashkiv LB. Use of RNA sequencing to evaluate rheumatic disease patients. Arthritis Res Ther. 2015 Jul 1;17(1):167. doi: 10.1186/s13075-015-0677-3. PMID: 26126608; PMCID: PMC4488125.

30: Naor D, Nedvetzki S. CD44 in rheumatoid arthritis. Arthritis Res Ther. 2003;5(3):105-15. doi: 10.1186/ar746. Epub 2003 Feb 28. PMID: 12723975; PMCID: PMC165042.

31: Jordan AR, Racine RR, Hennig MJ, Lokeshwar VB. The Role of CD44 in Disease Pathophysiology and Targeted Treatment. Front Immunol. 2015 Apr 21;6:182. doi: 10.3389/fimmu.2015.00182. PMID: 25954275; PMCID: PMC4404944.

32: Gill RB, Day A, Barstow A, Zaman G, Chenu C, Dhoot GK. Mammalian Sulf1 RNA alternative splicing and its significance to tumour growth regulation. Tumour Biol. 2012 Oct;33(5):1669-80. doi: 10.1007/s13277-012-0423-2. Epub 2012 Jun 15. PMID: 22700218.

33: Sarkissian M, Winne A, Lafyatis R. The mammalian homolog of suppressor-of-white-apricot regulates alternative mRNA splicing of CD45 exon 4 and fibronectin IIICS. J Biol Chem. 1996 Dec 6;271(49):31106-14. doi: 10.1074/jbc.271.49.31106.PMID: 8940107.

34: Chang HH, Tai TS, Lu B, Iannaccone C, Cernadas M, Weinblatt M, Shadick N, Miaw SC, Ho IC. PTPN22.6, a dominant negative isoform of PTPN22 and potential biomarker of rheumatoid arthritis. PLoS One. 2012;7(3):e33067. doi: 10.1371/journal.pone.0033067. Epub 2012 Mar 12. PMID: 22427951; PMCID: PMC3299735.

35: Torreggiani S, Torcoletti M, Campos-Xavier B, Baldo F, Agostoni C, Superti- Furga A, Filocamo G. Progressive pseudorheumatoid dysplasia: a rare childhood disease. Rheumatol Int. 2019 Mar;39(3):441-452. doi: 10.1007/s00296-018-4170-6.Epub 2018 Oct 16. PMID: 30327864.

36: Porola P, Mackiewicz Z, Laine M, Baretto G, Stegaev V, Takakubo Y, Takagi M, Ainola M, Konttinen YT. Laminin isoform profiles in salivary glands in Sjögren's syndrome. Adv Clin Chem. 2011;55:35-59. doi: 10.1016/b978-0-12-387042-1.00003-4.PMID: 22126023.

37: Chalaris A, Garbers C, Rabe B, Rose-John S, Scheller J. The soluble Interleukin 6 receptor: generation and role in inflammation and cancer. Eur J Cell Biol. 2011 Jun-Jul;90(6-7):484-94. doi: 10.1016/j.ejcb.2010.10.007. Epub 2010 Dec 8. PMID: 21145125.

38: Tardif G, Dupuis M, Reboul P, Geng CS, Pelletier JP, Ranger P, Martel-Pelletier J. Identification and differential expression of human collagenase-3 mRNA species derived from internal deletion, alternative splicing, and different polyadenylation and transcription initiation sites. Osteoarthritis Cartilage.2003 Jul;11(7):524-37. doi: 10.1016/s1063-4584(03)00079-7. PMID: 12814616.

39: Kozyrev SV, Alarcon-Riquelme ME. The genetics and biology of Irf5-mediated signaling in lupus. Autoimmunity. 2007 Dec;40(8):591-601. doi: 10.1080/08916930701510905. PMID: 18075793.

40: Kim S. Interleukin-32 in inflammatory autoimmune diseases. Immune Netw. 2014 Jun;14(3):123-7. doi: 10.4110/in.2014.14.3.123. Epub 2014 Jun 19. PMID: 24999308; PMCID: PMC4079818.

41: Morand EF. Effects of glucocorticoids on inflammation and arthritis. Curr Opin Rheumatol. 2007 May;19(3):302-7. doi: 10.1097/BOR.0b013e32805e87d0. PMID:17414960.

42: Li HZ, Lin Z, Xu XH, Lin N, Lu HD. The potential roles of circRNAs in osteoarthritis: a coming journey to find a treasure. Biosci Rep. 2018 Oct 31;38(5):BSR20180542. doi: 10.1042/BSR20180542. PMID: 30279209; PMCID: PMC6209588.

43: Christmas P, Ursino SR, Fox JW, Soberman RJ. Expression of the CYP4F3 gene. tissue-specific splicing and alternative promoters generate high and low K(m) forms of leukotriene B(4) omega-hydroxylase. J Biol Chem. 1999 Jul 23;274(30):21191-9. doi: 10.1074/jbc.274.30.21191. PMID: 10409674.

44: Arnér ES. Focus on mammalian thioredoxin reductases—important selenoproteins with versatile functions. Biochim Biophys Acta. 2009 Jun;1790(6):495-526. doi: 10.1016/j.bbagen.2009.01.014. Epub 2009 Feb 11. PMID:19364476.

45: Frisch RN, Curtis KM, Aenlle KK, Howard GA. Hepatocyte growth factor and alternative splice variants - expression, regulation and implications in osteogenesis and bone health and repair. Expert Opin Ther Targets. 2016 Sep;20(9):1087-98. doi: 10.1517/14728222.2016.1162293. Epub 2016 Mar 21. PMID:26941128; PMCID: PMC5031543.

46: Fan Y, Yang J, Xie S, He J, Huang S, Chen J, Jiang S, Yu L, Zhou Y, Cao X,Ji X, Zhang Y. Systematic analysis of inflammation and pain pathways in a mouse model of gout. Mol Pain. 2022 Jan-Dec;18:17448069221097760. doi: 10.1177/17448069221097760. PMID: 35430901; PMCID: PMC9069606.

47: Scheller J, Garbers C, Rose-John S. Interleukin-6: from basic biology to selective blockade of pro-inflammatory activities. Semin Immunol. 2014 Feb;26(1):2-12. doi: 10.1016/j.smim.2013.11.002. Epub 2013 Dec 8. PMID: 24325804.

48: Hersh EV, Lally ET, Moore PA. Update on cyclooxygenase inhibitors: has a third COX isoform entered the fray? Curr Med Res Opin. 2005 Aug;21(8):1217-26. doi: 10.1185/030079905X56367. PMID: 16083531.

49: Yan S, Sloane BF. Molecular regulation of human cathepsin B: implication in pathologies. Biol Chem. 2003 Jun;384(6):845-54. doi: 10.1515/BC.2003.095. PMID:12887051.

50: Odhams CA, Cortini A, Chen L, Roberts AL, Viñuela A, Buil A, Small KS,Dermitzakis ET, Morris DL, Vyse TJ, Cunninghame Graham DS.

Mapping eQTLs with RNA-seq reveals novel susceptibility genes, non-coding RNAs and alternative-splicing events in systemic lupus erythematosus. Hum Mol Genet. 2017 Mar1;26(5):1003-1017. doi: 10.1093/hmg/ddw417. PMID: 28062664; PMCID: PMC5409091.

51: Hashimoto T, Yasuda S, Koide H, Kataoka H, Horita T, Atsumi T, Koike T.Aberrant splicing of the hRasGRP4 transcript and decreased levels of this signaling protein in the peripheral blood mononuclear cells in a subset of patients with rheumatoid arthritis. Arthritis Res Ther. 2011;13(5):R154. doi: 10.1186/ar3470. Epub 2011 Sep 20. PMID: 21933395; PMCID: PMC3308084.

52: Baiyasi A, Barbosa J, Parendo A, Lin X. Pleiotropy of a Stickler syndrome genotype. Eur J Ophthalmol. 2022 Nov;32(6):NP10-NP12. doi:10.1177/11206721211035611. Epub 2021 Jul 27. PMID: 34313156.

53: Logsdon CD, Fuentes MK, Huang EH, Arumugam T. RAGE and RAGE ligands incancer. Curr Mol Med. 2007 Dec;7(8):777-89. doi: 10.2174/156652407783220697.PMID: 18331236.

54: Ward FJ, Dahal LN, Khanolkar RC, Shankar SP, Barker RN. Targeting the alternatively spliced soluble isoform of CTLA-4: prospects for immunotherapy? Immunotherapy. 2014;6(10):1073-84. doi: 10.2217/imt.14.73. PMID: 25428646.

55: Ball AK, Beilstein K, Wittmann S, Sürün D, Saul MJ, Schnütgen F, Flamand N,Capelo R, Kahnt AS, Frey H, Schaefer L, Marschalek R, Häfner AK, Steinhilber D. Characterization and cellular localization of human 5-lipoxygenase and its protein isoforms 5-LOΔ13, 5-LOΔ4 and 5-LOp12. Biochim Biophys Acta Mol Cell Biol Lipids. 2017 May;1862(5):561-571. doi: 10.1016/j.bbalip.2017.02.015. Epub 2017 Feb 28. PMID: 28257804.

56: Liu Q, Niu N, Wada Y, Liu J. The Role of Cdkn1A-Interacting Zinc Finger Protein 1 (CIZ1) in DNA Replication and Pathophysiology. Int J Mol Sci. 2016 Feb 5;17(2):212. doi: 10.3390/ijms17020212. PMID: 26861296; PMCID: PMC4783944.

57: Naor D, Sionov RV, Ish-Shalom D. CD44: structure, function, and association with the malignant process. Adv Cancer Res. 1997;71:241-319. doi:10.1016/s0065-230x(08)60101-3. PMID: 9111868.

58: Nollet M, Bachelier R, Joshkon A, Traboulsi W, Mahieux A, Moyon A, Muller A,Somasundaram I, Simoncini S, Peiretti F, Leroyer AS, Guillet B, Granel B,Dignat-George F, Bardin N, Foucault-Bertaud A, Blot-Chabaud M. Involvement of Multiple Variants of Soluble CD146 in Systemic Sclerosis: Identification of a Novel Profibrotic Factor. Arthritis Rheumatol. 2022 Jun;74(6):1027-1038. doi:10.1002/art.42063. Epub 2022 Apr 11. PMID: 35001552.

59: Turkkila M, Andersson KM, Amu S, Brisslert M, Erlandsson MC, Silfverswärd S,Bokarewa MI. Suppressed diversity of survivin splicing in active rheumatoid arthritis. Arthritis Res Ther. 2015 Jul 10;17:175. doi:10.1186/s13075-015-0689-z. PMID: 26160473; PMCID: PMC4702383.

60: Santos RF, Oliveira L, Carmo AM. Tuning T Cell Activation: The Function of CD6 At the Immunological Synapse and in T Cell Responses. Curr Drug Targets.2016;17(6):630-9. doi: 10.2174/1389450116666150531152439. PMID: 26028048.

61: Dinarello CA. Targeting interleukin 18 with interleukin 18 binding protein.Ann Rheum Dis. 2000 Nov;59 Suppl 1(Suppl 1):i17-20. doi:10.1136/ard.59.suppl_1.i17. PMID: 11053080; PMCID: PMC1766611.

62: Cao X, Li P, Song X, Shi L, Qin L, Chen D, Chu T, Cheng Y. PCBP1 isassociated with rheumatoid arthritis by affecting RNA products of genes involved in immune response in Th1 cells. Sci Rep. 2022 May 19;12(1):8398. doi:10.1038/s41598-022-12594-7. PMID: 35589811; PMCID: PMC9120163.

63: Barbezier N, Tessier FJ, Chango A. Le récepteur des produits de glycation avancée RAGE/AGER : une vue intégrative pour des applications en clinique [Receptor of advanced glycation endproducts RAGE/AGER: an integrative view for clinical applications]. Ann Biol Clin (Paris). 2014 Nov-Dec;72(6):669-80. French. doi: 10.1684/abc.2014.1010. PMID: 25486663.

64: Müller-Ladner U, Elices MJ, Kriegsmann JB, Strahl D, Gay RE, Firestein GS, Gay S. Alternatively spliced CS-1 fibronectin isoform and its receptor VLA-4 in rheumatoid arthritis synovium. J Rheumatol. 1997 Oct;24(10):1873-80. PMID: 9330926.

65: Atsumi T, Suzuki H, Jiang JJ, Okuyama Y, Nakagawa I, Ota M, Tanaka Y, Ohki T, Katsunuma K, Nakajima K, Hasegawa Y, Ohara O, Ogura H, Arima Y, Kamimura D, Murakami M. Rbm10 regulates inflammation development via alternative splicing of Dnmt3b. Int Immunol. 2017 Dec 31;29(12):581-591. doi: 10.1093/intimm/dxx067. PMID: 29309623.

66: Geroldi D, Falcone C, Emanuele E. Soluble receptor for advanced glycation end products: from disease marker to potential therapeutic target. Curr Med Chem. 2006;13(17):1971-8. doi: 10.2174/092986706777585013. PMID: 16842191.

67: Lin JD, Chao TC. Vascular endothelial growth factor in thyroid cancers. Cancer Biother Radiopharm. 2005 Dec;20(6):648-61. doi: 10.1089/cbr.2005.20.648. PMID: 16398617.

68: Hull KM, Drewe E, Aksentijevich I, Singh HK, Wong K, McDermott EM, Dean J, Powell RJ, Kastner DL. The TNF receptor-associated periodic syndrome (TRAPS): emerging concepts of an autoinflammatory disorder. Medicine (Baltimore). 2002 Sep;81(5):349-68. doi: 10.1097/00005792-200209000-00002. PMID: 12352631.

69: Wittig BM, Stallmach A, Zeitz M, Günthert U. Functional involvement of CD44 variant 7 in gut immune response. Pathobiology. 2002-2003;70(3):184-9. doi: 10.1159/000068152. PMID: 12571424.

70: Rose-John S, Waetzig GH, Scheller J, Grötzinger J, Seegert D. The IL-6/sIL-6R complex as a novel target for therapeutic approaches. Expert

OpinTher Targets. 2007 May;11(5):613-24. doi: 10.1517/14728222.11.5.613. PMID:17465721.

71: Du J, Qiao Y, Sun L, Wang X. Lymphoid-specific tyrosine phosphatase (Lyp): a potential drug target for treatment of autoimmune diseases. Curr Drug Targets.2014 Mar;15(3):335-46. doi: 10.2174/13894501113146660236. PMID: 24188455.

72: Ebbinghaus C, Scheuermann J, Neri D, Elia G. Diagnostic and therapeutic applications of recombinant antibodies: targeting the extra-domain B of fibronectin, a marker of tumor angiogenesis. Curr Pharm Des. 2004;10(13):1537-49. doi: 10.2174/1381612043384808. PMID: 15134574.

73: Cañete JD, Albaladejo C, Hernández MV, Laínez B, Pinto JA, Ramírez J, López- Armada MJ, Rodríguez-Cros JR, Engel P, Blanco FJ, Sanmartí R. Clinical significance of high levels of soluble tumour necrosis factor-α receptor-2 produced by alternative splicing in rheumatoid arthritis: a longitudinal prospective cohort study. Rheumatology (Oxford). 2011 Apr;50(4):721-8. doi: 10.1093/rheumatology/keq381. Epub 2010 Dec 6. PMID: 21134963.

74: Brenol CV, Veit TD, Chies JA, Xavier RM. The role of the HLA-G gene and molecule on the clinical expression of rheumatologic diseases. Rev Bras Reumatol. 2012 Jan-Feb;52(1):82-91. English, Portuguese. PMID: 22286648.

75: Hanson AL, Cuddihy T, Haynes K, Loo D, Morton CJ, Oppermann U, Leo P, Thomas GP, Lê Cao KA, Kenna TJ, Brown MA. Genetic Variants in ERAP1 and ERAP2 Associated With Immune-Mediated Diseases Influence Protein Expression and the Isoform Profile. Arthritis Rheumatol. 2018 Feb;70(2):255-265. doi:10.1002/art.40369. Epub 2017 Dec 29. PMID: 29108111.

76: Giblin SP, Schwenzer A, Midwood KS. Alternative splicing controls cell lineage-specific responses to endogenous innate immune triggers within the extracellular matrix. Matrix Biol. 2020 Nov;93:95-114. doi:10.1016/j.matbio.2020.06.003. Epub 2020 Jun 27. PMID: 32599145.

77: Friedman B, Larranaga-Vera A, Castro CM, Corciulo C, Rabbani P, Cronstein BN. Adenosine A2A receptor activation reduces chondrocyte senescence. FASEB J.2023 Apr;37(4):e22838. doi: 10.1096/fj.202201212RR. PMID: 36884388.

78: Mulcahy H, O'Rourke KP, Adams C, Molloy MG, O'Gara F. LST1 and NCR3 expression in autoimmune inflammation and in response to IFN-gamma, LPS and microbial infection. Immunogenetics. 2006 Jan;57(12):893-903. doi:10.1007/s00251-005-0057-2. Epub 2005 Dec 17. PMID: 16362817.

79: Micheau O. Cellular FLICE-inhibitory protein: an attractive therapeutic target? Expert Opin Ther Targets. 2003 Aug;7(4):559-73. doi:10.1517/14728222.7.4.559. PMID: 12885274; PMCID: PMC2984612.

80: Suzuki A, Terao C, Yamamoto K. Linking of genetic risk variants to disease-specific gene expression via multi-omics studies in rheumatoid arthritis. SeminArthritis Rheum. 2019 Dec;49(3S):S49-S53. doi: 10.1016/j.semarthrit.2019.09.007.PMID: 31779853.

81: Tang YP, Zhang QB, Dai F, Liao X, Dong ZR, Yi T, Qing YF. Circular RNAs in peripheral blood mononuclear cells from ankylosing spondylitis. Chin Med J (Engl). 2021 Oct 19;134(21):2573-2582. doi: 10.1097/CM9.0000000000001815. PMID:34670246; PMCID: PMC8577680.

82: Yoon HK, Byun HS, Lee H, Jeon J, Lee Y, Li Y, Jin EH, Kim J, Hong JH, Kim JH, Seok JH, Kang SW, Lee WH, Hur GM. Intron-derived aberrant splicing of A20 transcript in rheumatoid arthritis. Rheumatology (Oxford). 2013 Mar;52(3):427-37. doi: 10.1093/rheumatology/kes292. Epub 2012 Nov 12. PMID:23148088.

83: Błochowiak KJ, Trzybulska D, Olewicz-Gawlik A, Sikora JJ, Nowak-Gabryel M, Kocięcki J, Witmanowski H, Sokalski J. Levels of EGF and VEGF in patients with primary and secondary Sjögren's syndrome. Adv Clin Exp Med. 2018 Apr;27(4):455-461. doi: 10.17219/acem/70800. PMID: 29558032.

84: Nesterovitch AB, Hoffman MD, Simon M, Petukhov PA, Tharp MD, Glant TT.Mutations in the PSTPIP1 gene and aberrant splicing variants in patients with pyoderma gangrenosum. Clin Exp Dermatol. 2011 Dec;36(8):889-95. doi: 10.1111/j.1365-2230.2011.04137.x. Epub 2011 Jul 25. PMID: 21790734.

85: Naor D, Nedvetzki S, Walmsley M, Yayon A, Turley EA, Golan I, Caspi D, Sebban LE, Zick Y, Garin T, Karussis D, Assayag-Asherie N, Raz I, Weiss L, Slavin S, Golan I. CD44 involvement in autoimmune inflammations: the lesson to be learned from CD44-targeting by antibody or from knockout mice. Ann N Y Acad Sci. 2007 Sep;1110:233-47. doi: 10.1196/annals.1423.025. PMID: 17911438.

86: Liu X, Dong H, Gong Y, Wang L, Zhang R, Zheng T, Zheng Y, Shen S, Zheng C,Tian M, Liu N, Zhang X, Zheng QY. A Novel missense mutation of COL2A1 gene in a large family with stickler syndrome type I. J Cell Mol Med. 2022 Mar;26(5):1530-1539. doi: 10.1111/jcmm.17187. Epub 2022 Jan 21. PMID: 35064646; PMCID: PMC8899160.

87: Etem EO, Koca SS, Erol D, Yolbas S, Oz E, Elyas H, Isık A. Decreased MEFV gene expression in rheumatoid arthritis patients. Genet Mol Res. 2015 Feb 6;14(1):1000-7. doi: 10.4238/2015.February.6.3. PMID: 25730039.

88: Ramos HL, O'Shea JJ, Watford WT. STAT5 isoforms: controversies and clarifications. Biochem J. 2007 May 15;404(1):e1-2. doi: 10.1042/BJ2007420. PMID: 17447893; PMCID: PMC1868835.

89: Peffers MJ, Fang Y, Cheung K, Wei TK, Clegg PD, Birch HL. Transcriptome analysis of ageing in uninjured human Achilles tendon. Arthritis Res Ther. 2015 Feb 18;17(1):33. doi: 10.1186/s13075-015-0544-2. PMID: 25888722; PMCID: PMC4355574.

90: Yoo SA, Leng L, Kim BJ, Du X, Tilstam PV, Kim KH, Kong JS, Yoon HJ, Liu A, Wang T, Song Y, Sauler M, Bernhagen J, Ritchlin CT, Lee P, Cho CS, Kim WU, Bucala R. MIF allele-dependent regulation of the MIF coreceptor CD44 and role in rheumatoid arthritis. Proc Natl Acad Sci U S A. 2016 Dec 6;113(49):E7917-E7926. doi: 10.1073/pnas.1612717113. Epub 2016 Nov 21. PMID: 27872288; PMCID: PMC5150393.

91: Devauchelle V, Essabbani A, De Pinieux G, Germain S, Tourneur L, Mistou S,Margottin-Goguet F, Anract P, Migaud H, Le Nen D, Lequerré T, Saraux A, Dougados M, Breban M, Fournier C, Chiocchia G. Characterization and functional consequences of underexpression of clusterin in rheumatoid arthritis. J Immunol. 2006 Nov 1;177(9):6471-9. doi: 10.4049/jimmunol.177.9.6471. PMID: 17056579.

92: Huang F, Yamaguchi A, Tsuchiya N, Ikawa T, Tamura N, Virtala MM, Granfors K,Yasaei P, Yu DT. Induction of alternative splicing of HLA-B27 by bacterialinvasion. Arthritis Rheum. 1997 Apr;40(4):694-703. doi: 10.1002/art.1780400414.PMID: 9125251.

93: Venkatachalam KV. Human 3'-phosphoadenosine 5'-phosphosulfate (PAPS)synthase: biochemistry, molecular biology and genetic deficiency. IUBMB Life.2003 Jan;55(1):1-11. doi: 10.1080/1521654031000072148. PMID: 12716056.

94: Lemaire R, Winne A, Sarkissian M, Lafyatis R. SF2 and SRp55 regulation of CD45 exon 4 skipping during T cell activation. Eur J Immunol. 1999 Mar;29(3):823-37. doi: 10.1002/(SICI)1521-4141(199903)29:03<823::AID-IMMU823>3.0.CO;2-C. PMID: 10092085.

95: Hulse RP, Drake RA, Bates DO, Donaldson LF. The control of alternativesplicing by SRSF1 in myelinated afferents contributes to the development of neuropathic pain. Neurobiol Dis. 2016 Dec;96:186-200. doi:10.1016/j.nbd.2016.09.009. Epub 2016 Sep 9. PMID: 27616424; PMCID: PMC5113660.

96: Muys BR, Shrestha RL, Anastasakis DG, Pongor L, Li XL, Grammatikakis I,Polash A, Chari R, Gorospe M, Harris CC, Aladjem MI, Basrai MA, Hafner M, Lal A.Matrin3 regulates mitotic spindle dynamics by controlling alternative splicing of CDC14B. Cell Rep. 2023 Mar 28;42(3):112260. doi:10.1016/j.celrep.2023.112260. Epub 2023 Mar 15. PMID: 36924503; PMCID:PMC10132239.

97: Rong J, Yin J, Su Z. Natural antisense RNAs are involved in the regulation of CD45 expression in autoimmune diseases. Lupus. 2015 Mar;24(3):235-9. doi: 10.1177/0961203314558856. Epub 2014 Nov 7. PMID: 25381328.

98: Crawford AH, Hildyard JCW, Rushing SAM, Wells DJ, Diez-Leon M, Piercy RJ. Validation of DE50-MD dogs as a model for the brain phenotype of Duchenne muscular dystrophy. Dis Model Mech. 2022 Mar 1;15(3):dmm049291. doi: 10.1242/dmm.049291. Epub 2022 Mar 2. PMID: 35019137; PMCID: PMC8906169.

99: Qin Z, Qin L, Feng X, Li Z, Bian J. Development of Cdc2-like Kinase 2 Inhibitors: Achievements and Future Directions. J Med Chem. 2021 Sep

23;64(18):13191-13211. doi: 10.1021/acs.jmedchem.1c00985. Epub 2021 Sep 14. PMID: 34519506.

100: Hitomi Y, Aiba Y, Ueno K, Nishida N, Kawai Y, Kawashima M, Tsuiji M, Iwabuchi C, Takada S, Miyake N, Nagasaki M, Tokunaga K, Nakamura M. rs2013278 in the multiple immunological-trait susceptibility locus CD28 regulates the production of non-functional splicing isoforms. Hum Genomics. 2022 Oct 21;16(1):46. doi: 10.1186/s40246-022-00419-7. PMID: 36271469; PMCID: PMC9585755.

101: Ramsay RG, Ciznadija D, Vanevski M, Mantamadiotis T. Transcriptional regulation of cyclo-oxygenase expression: three pillars of control. Int J Immunopathol Pharmacol. 2003 May-Aug;16(2 Suppl):59-67. PMID: 14552705.

102: Aigner T, Bartnik E, Sohler F, Zimmer R. Functional genomics of osteoarthritis: on the way to evaluate disease hypotheses. Clin Orthop Relat Res. 2004 Oct;(427 Suppl):S138-43. PMID: 15480056.

103: Jones DC, Roghanian A, Brown DP, Chang C, Allen RL, Trowsdale J, Young NT. Alternative mRNA splicing creates transcripts encoding soluble proteins from most LILR genes. Eur J Immunol. 2009 Nov;39(11):3195-206. doi: 10.1002/eji.200839080. PMID: 19658091.

104: Evans CH, Robbins PD. The interleukin-1 receptor antagonist and its delivery by gene transfer. Receptor. 1994 Spring;4(1):9-15. PMID: 8038709. 105: Choi JD, Bae SY, Hong JW, Azam T, Dinarello CA, Her E,

105: Choi WS, Kim BK, Lee CK, Yoon DY, Kim SJ, Kim SH. Identification of the most active interleukin-32 isoform. Immunology. 2009 Apr;126(4):535-42. doi: 10.1111/j.1365-2567.2008.02917.x. Epub 2008 Sep 2. PMID: 18771438; PMCID: PMC2673365.

106: Nichols RC, Raben N, Boerkoel CF, Plotz PH. Human isoleucyl-tRNA synthetase: sequence of the cDNA, alternative mRNA splicing, and the characteristics of an unusually long C-terminal extension. Gene. 1995 Apr 3;155(2):299-304. doi: 10.1016/0378-1119(94)00634-5. PMID: 7721108.

107: Palangat M, Anastasakis DG, Fei DL, Lindblad KE, Bradley R, Hourigan CS, Hafner M, Larson DR. The splicing factor U2AF1 contributes to cancer progression through a noncanonical role in translation regulation. Genes Dev. 2019 May 1;33(9-10):482-497. doi: 10.1101/gad.319590.118. Epub 2019 Mar 6. PMID: 30842218; PMCID: PMC6499322.

108: Ward FJ, Dahal LN, Wijesekera SK, Abdul-Jawad SK, Kaewarpai T, Xu H, Vickers MA, Barker RN. The soluble isoform of CTLA-4 as a regulator of T-cell responses. Eur J Immunol. 2013 May;43(5):1274-85. doi: 10.1002/eji.201242529. Epub 2013 Mar 6. PMID: 23400950.

109: Gouttenoire J, Valcourt U, Ronzière MC, Aubert-Foucher E, Mallein-Gerin F, Herbage D. Modulation of collagen synthesis in normal and osteoarthritic cartilage. Biorheology. 2004;41(3-4):535-42. PMID: 15299284.

110: Bottini N, Bottini E, Gloria-Bottini F, Mustelin T. Low-molecular-weight protein tyrosine phosphatase and human disease: in search of biochemical mechanisms. Arch Immunol Ther Exp (Warsz). 2002;50(2):95-104. PMID: 12022706.

111: Sterenczak KA, Willenbrock S, Barann M, Klemke M, Soller JT, Eberle N, Nolte I, Bullerdiek J, Murua Escobar H. Cloning, characterisation, and comparative quantitative expression analyses of receptor for advanced glycation end products (RAGE) transcript forms. Gene. 2009 Apr 1;434(1-2):35-42. doi: 10.1016/j.gene.2008.10.027. Epub 2008 Nov 12. PMID: 19061941.

112: Hattori M, Yabuuchi A, Tanaka H, Kawara T, Wang H, Inoue K, Shiozawa S, Komai K. Expression of ASC splice variant found in Japanese patients with palindromic rheumatism is regulated by rs8056505 single nucleotide polymorphism and interleukin-1 beta. Asian Pac J Allergy Immunol. 2022 Aug 22. doi: 10.12932/AP-010322-1339. Epub ahead of print. PMID: 35964242.

113: Wang S, Dong H, Han J, Ho WT, Fu X, Zhao ZJ. Identification of a variant form of tyrosine phosphatase LYP. BMC Mol Biol. 2010 Nov 2;11:78. doi: 10.1186/1471-2199-11-78. PMID: 21044313; PMCID: PMC2987843.

114: Ichinose K, Zhang Z, Koga T, Juang YT, Kis-Tóth K, Sharpe AH, Kuchroo V, Crispín JC, Tsokos GC. Brief report: increased expression of a short splice variant of CTLA-4 exacerbates lupus in MRL/lpr mice. Arthritis Rheum. 2013 Mar;65(3):764-9. doi: 10.1002/art.37790. PMID: 23203389; PMCID: PMC3582760.

115: Watanabe H, Kuroki K, Yamada C, Saburi Y, Maeda N, Maenaka K. Therapeutic effects of soluble human leukocyte antigen G2 isoform in lupus-prone MRL/lpr mice. Hum Immunol. 2020 Apr;81(4):186-190. doi: 10.1016/j.humimm.2019.11.002. Epub 2019 Nov 14. PMID: 31733925.

116: Ramírez-Bello J, Vargas-Alarcón G, Tovilla-Zárate C, Fragoso JM. Polimorfismos de un solo nucleótido (SNP): implicaciones funcionales de los SNP reguladores (rSNP) y de los SNP-ARN estructurales (srSNP) en enfermedades complejas [Single nucleotide polymorphisms (SNPs): functional implications of regulatory-SNP (rSNP) and structural RNA (srSNPs) in complex diseases]. Gac Med Mex. 2013 Mar-Apr;149(2):220-8. Spanish. PMID: 23652189.

117: Nakajima M, Miyamoto Y, Ikegawa S. Cloning and characterization of the osteoarthritis-associated gene DVWA. J Bone Miner Metab. 2011 May;29(3):300-8. doi: 10.1007/s00774-010-0230-z. Epub 2010 Nov 6. PMID: 21057832.

118: van Hoolwerff M, Tuerlings M, Wijnen IJL, Suchiman HED, Cats D, Mei H, Nelissen RGHH, van der Linden-van der Zwaag HMJ, Ramos YFM, Coutinho de Almeida R, Meulenbelt I. Identification and functional characterization of imbalanced osteoarthritis-associated fibronectin splice variants. Rheumatology (Oxford). 2023 Feb 1;62(2):894-904. doi: 10.1093/rheumatology/keac272. PMID: 35532170; PMCID: PMC9891405.

119: Turner MW, Hamvas RM. Mannose-binding lectin: structure, function, genetics and disease associations. Rev Immunogenet. 2000;2(3):305-22. PMID: 11256742.

120: Parker AE, Boutell J, Carr A, Maciewicz RA. Novel cartilage-specific splice variants of fibronectin. Osteoarthritis Cartilage. 2002 Jul;10(7):528-34. doi: 10.1053/joca.2002.0792. PMID: 12127832.

121: Sztrolovics R, Grover J, Cs-Szabo G, Shi SL, Zhang Y, Mort JS, Roughley PJ. The characterization of versican and its message in human articular cartilage and intervertebral disc. J Orthop Res. 2002 Mar;20(2):257-66. doi: 10.1016/S0736-0266(01)00110-3. PMID: 11918305.

122: Peffers MJ, Collins J, Fang Y, Goljanek-Whysall K, Rushton M, Loughlin J, Proctor C, Clegg PD. Age-related changes in mesenchymal stem cells identified using a multi-omics approach. Eur Cell Mater. 2016 Feb 8;31:136-59. doi: 10.22203/ecm.v031a10. PMID: 26853623.

123: Ryder LR, Bartels EM, Woetmann A, Madsen HO, Odum N, Bliddal H, Danneskiold-Samsøe B, Ribel-Madsen S, Ryder LP. FoxP3 mRNA splice forms in synovial CD4+ T cells in rheumatoid arthritis and psoriatic arthritis. APMIS. 2012 May;120(5):387-96. doi: 10.1111/j.1600-0463.2011.02848.x. Epub 2011 Dec 14. PMID: 22515293.

124: Toussirot E, Saas P, Deschamps M, Pouthier F, Perrot L, Perruche S, Chabod J, Tiberghien P, Wendling D. Increased production of soluble CTLA-4 in patients with spondylarthropathies correlates with disease activity. Arthritis Res Ther. 2009;11(4):R101. doi: 10.1186/ar2747. Epub 2009 Jul 1. PMID: 19570209; PMCID: PMC2745776.

125: Lee S, Kim S, Bae S, Choi J, Hong J, Ryoo S, Jhun H, Hong K, Kim E, Jo S, Her E, Kim S. Interleukin-32 gamma specific monoclonal antibody and developing IL-32 specific ELISA. Hybridoma (Larchmt). 2010 Dec;29(6):501-9. doi: 10.1089/hyb.2010.0059. Epub 2010 Nov 18. PMID: 21087097.

126: Seth P, Yeowell HN. Fox-2 protein regulates the alternative splicing of scleroderma-associated lysyl hydroxylase 2 messenger RNA. Arthritis Rheum. 2010 Apr;62(4):1167-75. doi: 10.1002/art.27315. PMID: 20131247; PMCID: PMC2915826.

127: Grisar J, Munk M, Steiner CW, Amoyo-Minar L, Tohidast-Akrad M, Zenz P, Steiner G, Smolen JS. Expression patterns of CD44 and CD44 splice variants in patients with rheumatoid arthritis. Clin Exp Rheumatol. 2012 Jan- Feb;30(1):64-72. Epub 2012 Mar 6. PMID: 22261341.

128: Lamana A, Ortiz AM, Alvaro-Gracia JM, Díaz-Sánchez B, Novalbos J, García- Vicuña R, González-Alvaro I. Characterization of serum interleukin-15 in healthy volunteers and patients with early arthritis to assess its potential use as a biomarker. Eur Cytokine Netw. 2010 Sep;21(3):186-94. doi: 10.1684/ecn.2010.0203. Epub 2010 Aug 24. PMID: 20736143.

129: Friese MA, Hellwage J, Jokiranta TS, Meri S, Müller-Quernheim HJ, Peter HH, Eibel H, Zipfel PF. Different regulation of factor H and FHL-

1/reconectin by inflammatory mediators and expression of the two proteins in rheumatoid arthritis (RA). Clin Exp Immunol. 2000 Aug;121(2):406-15. doi: 10.1046/j.1365-2249.2000.01285.x. PMID: 10931160; PMCID: PMC1905714.

130: Jin P, Zhang J, Sumariwalla PF, Ni I, Jorgensen B, Crawford D, Phillips S, Feldmann M, Shepard HM, Paleolog EM. Novel splice variants derived from the receptor tyrosine kinase superfamily are potential therapeutics for rheumatoid arthritis. Arthritis Res Ther. 2008;10(4):R73. doi: 10.1186/ar2447. Epub 2008 Jul 1. PMID: 18593464; PMCID: PMC2575619.

131: Zikherman J, Weiss A. Alternative splicing of CD45: the tip of the iceberg. Immunity. 2008 Dec 19;29(6):839-41. doi: 10.1016/j.immuni.2008.12.005. PMID: 19100695.

132: Dahal LN, Basu N, Youssef H, Khanolkar RC, Barker RN, Erwig LP, Ward FJ. Immunoregulatory soluble CTLA-4 modifies effector T-cell responses in systemic lupus erythematosus. Arthritis Res Ther. 2016 Aug 4;18:180. doi: 10.1186/s13075-016-1075-1. PMID: 27487771; PMCID: PMC4973056.

133: Hirata T, Usui T, Kobayashi S, Mimori T. A novel splice variant of human L-selectin encodes a soluble molecule that is elevated in serum of patients with rheumatic diseases. Biochem Biophys Res Commun. 2015 Jul 10;462(4):371-7. doi: 10.1016/j.bbrc.2015.05.002. Epub 2015 May 14. PMID: 25982478.

134: Salter DM, Godolphin JL, Gourlay MS, Lawson MF, Hughes DE, Dunne E. Analysis of human articular chondrocyte CD44 isoform expression and function in health and disease. J Pathol. 1996 Aug;179(4):396-402. doi: 10.1002/(SICI)1096-9896(199608)179:4<396::AID-PATH606>3.0.CO;2-G. PMID: 8869287.

135: Takahashi A, Kuroki K, Okabe Y, Kasai Y, Matsumoto N, Yamada C, Takai T, Ose T, Kon S, Matsuda T, Maenaka K. The immunosuppressive effect of domain- deleted dimer of HLA-G2 isoform in collagen-induced arthritis mice. Hum Immunol. 2016 Sep;77(9):754-9. doi: 10.1016/j.humimm.2016.01.010. Epub 2016 Jan 21. PMID: 26805457.

136: Korniejewska A, McKnight AJ, Johnson Z, Watson ML, Ward SG. Expression and agonist responsiveness of CXCR3 variants in human T lymphocytes. Immunology. 2011 Apr;132(4):503-15. doi: 10.1111/j.1365-2567.2010.03384.x. Epub 2011 Jan 24. PMID: 21255008; PMCID: PMC3075504.

137: De Arras L, Laws R, Leach SM, Pontis K, Freedman JH, Schwartz DA, Alper S. Comparative genomics RNAi screen identifies Eftud2 as a novel regulator of innate immunity. Genetics. 2014 Jun;197(2):485-96. doi: 10.1534/genetics.113.160499. Epub 2013 Dec 20. PMID: 24361939; PMCID: PMC4063909.

138: Nascimento A, Bruels CC, Donkervoort S, Foley AR, Codina A, Milisenda JC, Estrella EA, Li C, Pijuan J, Draper I, Hu Y, Stafki SA, Pais LS, Ganesh VS, O'Donnell-Luria A, Syeda SB, Carrera-García L,

Expósito-Escudero J, Yubero D, Martorell L, Pinal-Fernandez I, Lidov HGW, Mammen AL, Grau-Junyent JM, Ortez C, Palau F, Ghosh PS, Darras BT, Jou C, Kunkel LM, Hoenicka J, Bönnemann CG, Kang PB, Natera-de Benito D. Variants in DTNA cause a mild, dominantly inherited muscular dystrophy. Acta Neuropathol. 2023 Apr;145(4):479-496. doi: 10.1007/s00401-023-02551-7. Epub 2023 Feb 17. PMID: 36799992.

139: Kim JY, Yoon HK, Song ST, Park SR, Shim SC. Expression of activation- induced cytidine deaminase splicing variants in patients with ankylosing spondylitis. Autoimmunity. 2017 Dec;50(8):435-440. doi: 10.1080/08916934.2017.1385777. Epub 2017 Sep 29. PMID: 28959900.

140: Jones SA, Horiuchi S, Topley N, Yamamoto N, Fuller GM. The soluble interleukin 6 receptor: mechanisms of production and implications in disease. FASEB J. 2001 Jan;15(1):43-58. doi: 10.1096/fj.99-1003rev. PMID: 11149892.

141: Sender LY, Gibbert K, Suezer Y, Radeke HH, Kalinke U, Waibler Z. CD40 ligand-triggered human dendritic cells mount interleukin-23 responses that are further enhanced by danger signals. Mol Immunol. 2010 Mar;47(6):1255-61. doi: 10.1016/j.molimm.2009.12.008. Epub 2010 Jan 13. PMID: 20071030.

142: Aigner T, Zien A, Hanisch D, Zimmer R. Gene expression in chondrocytes assessed with use of microarrays. J Bone Joint Surg Am. 2003;85-A Suppl 2:117-23. doi: 10.2106/00004623-200300002-00016. PMID: 12721354.

143: Sun W, Xiao X, Li S, Jia X, Zhang Q. A novel deep intronic COL2A1 mutation in a family with early-onset high myopia/ocular-only Stickler syndrome. Ophthalmic Physiol Opt. 2020 May;40(3):281-288. doi: 10.1111/opo.12682. Epub 2020 Mar 20. PMID: 32196734.

144: Lainez B, Fernandez-Real JM, Romero X, Esplugues E, Cañete JD, Ricart W, Engel P. Identification and characterization of a novel spliced variant that encodes human soluble tumor necrosis factor receptor 2. Int Immunol. 2004 Jan;16(1):169-77. doi: 10.1093/intimm/dxh014. PMID: 14688072.

145: Schoonheim PJ, Chatzopoulou A, Schaaf MJ. The zebrafish as an in vivo model system for glucocorticoid resistance. Steroids. 2010 Dec;75(12):918-25. doi: 10.1016/j.steroids.2010.05.010. Epub 2010 May 21. PMID: 20493895.

146: Badot V, Durez P, Van den Eynde BJ, Nzeusseu-Toukap A, Houssiau FA, Lauwerys BR. Rheumatoid arthritis synovial fibroblasts produce a soluble form of the interleukin-7 receptor in response to pro-inflammatory cytokines. J Cell Mol Med. 2011 Nov;15(11):2335-42. doi: 10.1111/j.1582-4934.2010.01228.x. PMID: 21129157; PMCID: PMC3822945.

147: Scanzello CR, Markova DZ, Chee A, Xiu Y, Adams SL, Anderson G, Zgonis M, Qin L, An HS, Zhang Y. Fibronectin splice variation in human knee cartilage, meniscus and synovial membrane: observations in osteoarthritic knee. J Orthop Res. 2015 Apr;33(4):556-62. doi:

10.1002/jor.22787. Epub 2015 Jan 6. PMID: 25410897; PMCID: PMC4586164.

148: Zwicky R, Müntener K, Goldring MB, Baici A. Cathepsin B expression and down-regulation by gene silencing and antisense DNA in human chondrocytes. Biochem J. 2002 Oct 1;367(Pt 1):209-17. doi: 10.1042/BJ20020210. PMID: 12086583; PMCID: PMC1222861.

149: Feng D, Stone RC, Eloranta ML, Sangster-Guity N, Nordmark G, Sigurdsson S, Wang C, Alm G, Syvänen AC, Rönnblom L, Barnes BJ. Genetic variants and disease- associated factors contribute to enhanced interferon regulatory factor 5 expression in blood cells of patients with systemic lupus erythematosus. Arthritis Rheum. 2010 Feb;62(2):562-73. doi: 10.1002/art.27223. PMID: 20112383; PMCID: PMC3213692.

150: Ni Choileain S, Weyand NJ, Neumann C, Thomas J, So M, Astier AL. The dynamic processing of CD46 intracellular domains provides a molecular rheostat for T cell activation. PLoS One. 2011 Jan 19;6(1):e16287. doi: 10.1371/journal.pone.0016287. PMID: 21283821; PMCID: PMC3023775.

151: Nambiar MP, Enyedy EJ, Warke VG, Krishnan S, Dennis G, Wong HK, Kammer GM, Tsokos GC. T cell signaling abnormalities in systemic lupus erythematosus are associated with increased mutations/polymorphisms and splice variants of T cell receptor zeta chain messenger RNA. Arthritis Rheum. 2001 Jun;44(6):1336-50. doi: 10.1002/1529-0131(200106)44:6<1336::AID-ART226>3.0.CO;2-8. PMID: 11407693.

152: Ahmed S, Marotte H, Kwan K, Ruth JH, Campbell PL, Rabquer BJ, Pakozdi A, Koch AE. Epigallocatechin-3-gallate inhibits IL-6 synthesis and suppresses transsignaling by enhancing soluble gp130 production. Proc Natl Acad Sci U S A. 2008 Sep 23;105(38):14692-7. doi: 10.1073/pnas.0802675105. Epub 2008 Sep 16. PMID: 18796608; PMCID: PMC2567200.

153: Bestall SM, Hulse RP, Blackley Z, Swift M, Ved N, Paton K, Beazley-Long N, Bates DO, Donaldson LF. Sensory neuronal sensitisation occurs through HMGB-1-RAGE and TRPV1 in high-glucose conditions. J Cell Sci. 2018 Jul 26;131(14):jcs215939. doi: 10.1242/jcs.215939. PMID: 29930087; PMCID: PMC6080605.

154: Bachmann M, Hilker M, Grölz D, Tellmann G, Hake U, Kater L, de Wilde P, Tröster H. Different La/SS-B mRNA isoforms are expressed in salivary gland tissue of patients with primary Sjögren's syndrome. J Autoimmun. 1996 Dec;9(6):757-66. doi: 10.1006/jaut.1996.0098. PMID: 9115578.

155: Jéru I, Papin S, L'hoste S, Duquesnoy P, Cazeneuve C, Camonis J, Amselem S. Interaction of pyrin with 14.3.3 in an isoform-specific and phosphorylation- dependent manner regulates its translocation to the nucleus. Arthritis Rheum. 2005 Jun;52(6):1848-57. doi: 10.1002/art.21050. PMID: 15934090.

156: Giuliani AL, Colognesi D, Ricco T, Roncato C, Capece M, Amoroso F, Wang QG, De Marchi E, Gartland A, Di Virgilio F, Adinolfi E. Trophic activity of human P2X7 receptor isoforms A and B in osteosarcoma. PLoS One. 2014 Sep 16;9(9):e107224. doi: 10.1371/journal.pone.0107224. PMID: 25226385; PMCID: PMC4165768.

157: Rafael MS, Cavaco S, Viegas CS, Santos S, Ramos A, Willems BA, Herfs M, Theuwissen E, Vermeer C, Simes DC. Insights into the association of Gla-rich protein and osteoarthritis, novel splice variants and γ-carboxylation status. Mol Nutr Food Res. 2014 Aug;58(8):1636-46. doi: 10.1002/mnfr.201300941. Epub 2014 May 28. PMID: 24867294.

158: Rousseau JC, Sandell LJ, Delmas PD, Garnero P. Development and clinical application in arthritis of a new immunoassay for serum type IIA procollagen NH2 propeptide. Methods Mol Med. 2004;101:25-37. doi: 10.1385/1-59259-821-8:025. PMID: 15299208.

159: Peffers MJ, Goljanek-Whysall K, Collins J, Fang Y, Rushton M, Loughlin J, Proctor C, Clegg PD. Decoding the Regulatory Landscape of Ageing in Musculoskeletal Engineered Tissues Using Genome-Wide DNA Methylation and RNASeq. PLoS One. 2016 Aug 17;11(8):e0160517. doi: 10.1371/journal.pone.0160517. PMID: 27533049; PMCID: PMC4988628.

160: Zabeau L, Jensen CJ, Seeuws S, Venken K, Verhee A, Catteeuw D, van Loo G, Chen H, Walder K, Hollis J, Foote S, Morris MJ, Van der Heyden J, Peelman F, Oldfield BJ, Rubio JP, Elewaut D, Tavernier J. Leptin's metabolic and immune functions can be uncoupled at the ligand/receptor interaction level. Cell Mol Life Sci. 2015 Feb;72(3):629-644. doi: 10.1007/s00018-014-1697-x. Epub 2014 Aug 7. PMID: 25098352; PMCID: PMC4293488.

161: Schwager K, Villa A, Rösli C, Neri D, Rösli-Khabas M, Moser G. A comparative immunofluorescence analysis of three clinical-stage antibodies in head and neck cancer. Head Neck Oncol. 2011 May 8;3:25. doi: 10.1186/1758-3284-3-25. PMID: 21548989; PMCID: PMC3108933.

162: Polgár A, Brózik M, Tóth S, Holub M, Hegyi K, Kádár A, Hodinka L, Falus A. Soluble interleukin-6 receptor in plasma and in lymphocyte culture supernatants of healthy individuals and patients with systemic lupus erythematosus and rheumatoid arthritis. Med Sci Monit. 2000 Jan-Feb;6(1):13-8. PMID: 11208277.

163: Misener VL, Hui C, Malapitan IA, Ittel ME, Joyner AL, Jongstra J. Expression of mouse LSP1/S37 isoforms. S37 is expressed in embryonic mesenchymal cells. J Cell Sci. 1994 Dec;107 (Pt 12):3591-600. doi: 10.1242/jcs.107.12.3591. PMID: 7706408.

164: Kriegsmann J, Berndt A, Hansen T, Borsi L, Zardi L, Bräuer R, Petrow PK, Otto M, Kirkpatrick CJ, Gay S, Kosmehl H. Expression of fibronectin splice variants and oncofetal glycosylated fibronectin in the synovial membranes of patients with rheumatoid arthritis and osteoarthritis. Rheumatol Int. 2004 Jan;24(1):25-33. doi: 10.1007/s00296-003-0316-1. Epub 2003 Apr 24. PMID: 12712258.

165: Malik NM, Jin P, Raatz Y, Sumariwalla PF, Kiriakidis S, Shepard M, Feldmann M, Paleolog EM. Regulation of the angiopoietin-Tie ligand-receptor system with a novel splice variant of Tie1 reduces the severity of murine arthritis. Rheumatology (Oxford). 2010 Oct;49(10):1828-39. doi: 10.1093/rheumatology/keq163. Epub 2010 Jun 14. PMID: 20547659.

166: Madden J, Shearman CP, Dunn RL, Dastur ND, Tan RM, Nash GB, Rainger GE, Brunner A, Calder PC, Grimble RF. Altered monocyte CD44 expression in peripheral arterial disease is corrected by fish oil supplementation. Nutr Metab Cardiovasc Dis. 2009 May;19(4):247-52. doi: 10.1016/j.numecd.2008.06.011. Epub 2008 Sep 19. PMID: 18804988.

167: Ebe H, Matsumoto I, Kawaguchi H, Kurata I, Tanaka Y, Inoue A, Kondo Y, Tsuboi H, Sumida T. Clinical and functional significance of STEAP4-splice variant in CD14⁺ monocytes in patients with rheumatoid arthritis. Clin Exp Immunol. 2018 Mar;191(3):338-348. doi: 10.1111/cei.13076. Epub 2017 Nov 16. PMID: 29080328; PMCID: PMC5801491.

168: Wu J, Edberg JC, Gibson AW, Tsao B, Kimberly RP. Single-nucleotide polymorphisms of T cell receptor zeta chain in patients with systemic lupus erythematosus. Arthritis Rheum. 1999 Dec;42(12):2601-5. doi: 10.1002/1529-0131(199912)42:12<2601::AID-ANR13>3.0.CO;2-4. PMID: 10616006.

169: Suzuki Y, Ohya S, Yamamura H, Giles WR, Imaizumi Y. A New Splice Variant of Large Conductance Ca2+-activated K+ (BK) Channel α Subunit Alters Human Chondrocyte Function. J Biol Chem. 2016 Nov 11;291(46):24247-24260. doi: 10.1074/jbc.M116.743302. Epub 2016 Oct 7. PMID: 27758860; PMCID: PMC5104946.

170: Du M, Roy KM, Zhong L, Shen Z, Meyers HE, Nichols RC. VEGF gene expression is regulated post-transcriptionally in macrophages. FEBS J. 2006 Feb;273(4):732-45. doi: 10.1111/j.1742-4658.2006.05106.x. PMID: 16441660.

171: Friese MA, Hellwage J, Jokiranta TS, Meri S, Peter HH, Eibel H, Zipfel PF. FHL-1/reconectin and factor H: two human complement regulators which are encoded by the same gene are differently expressed and regulated. Mol Immunol. 1999 Sep- Oct;36(13-14):809-18. doi: 10.1016/s0161-5890(99)00101-7. PMID: 10698334.

172: Baici A, Lang A, Zwicky R, Müntener K. Cathepsin B in osteoarthritis: uncontrolled proteolysis in the wrong place. Semin Arthritis Rheum. 2005 Jun;34(6 Suppl 2):24-8. doi: 10.1016/j.semarthrit.2004.03.008. PMID: 16206953.

173: AlFadhli S, Nizam R. Differential expression of alternative splice variants of CTLA4 in Kuwaiti autoimmune disease patients. Gene. 2014 Jan 25;534(2):307-12. PMID: 24498648.

174: Bauer S, Jendro MC, Wadle A, Kleber S, Stenner F, Dinser R, Reich A, Faccin E, Gödde S, Dinges H, Müller-Ladner U, Renner C. Fibroblast activation protein is expressed by rheumatoid myofibroblast-like

synoviocytes. Arthritis Res Ther. 2006;8(6):R171. doi: 10.1186/ar2080. PMID: 17105646; PMCID: PMC1794515.

175: Horiuchi S, Ampofo W, Koyanagi Y, Yamashita A, Waki M, Matsumoto A, Yamamoto M, Yamamoto N. High-level production of alternatively spliced soluble interleukin-6 receptor in serum of patients with adult T-cell leukaemia/HTLV-I- associated myelopathy. Immunology. 1998 Nov;95(3):360-9. doi: 10.1046/j.1365-2567.1998.00622.x. PMID: 9824498; PMCID: PMC1364401.

176: Muller IB, Lin M, Lems WF, Ter Wee MM, Wojtuszkiewicz A, Nurmohamed MT, Cloos J, Assaraf YG, Jansen G, de Jonge R. Association of altered folylpolyglutamate synthetase pre-mRNA splicing with methotrexate unresponsiveness in early rheumatoid arthritis. Rheumatology (Oxford). 2021 Mar 2;60(3):1273-1281. doi: 10.1093/rheumatology/keaa428. PMID: 32940699; PMCID: PMC7937028.

177: Chiba T, Miyashita K, Sugoh T, Warita T, Inoko H, Kimura M, Sato T. IκBL, a novel member of the nuclear IκB family, inhibits inflammatory cytokine expression. FEBS Lett. 2011 Nov 16;585(22):3577-81. doi: 10.1016/j.febslet.2011.10.024. Epub 2011 Oct 20. PMID: 22024480.

178: Hulse RP, Beazley-Long N, Ved N, Bestall SM, Riaz H, Singhal P, Ballmer Hofer K, Harper SJ, Bates DO, Donaldson LF. Vascular endothelial growth factor-A165b prevents diabetic neuropathic pain and sensory neuronal degeneration. Clin Sci (Lond). 2015 Oct 1;129(8):741-56. doi: 10.1042/CS20150124. PMID: 26201024.

179: Punwani D, Wang H, Chan AY, Cowan MJ, Mallott J, Sunderam U, Mollenauer M, Srinivasan R, Brenner SE, Mulder A, Claas FH, Weiss A, Puck JM. Combined immunodeficiency due to MALT1 mutations, treated by hematopoietic cell transplantation. J Clin Immunol. 2015 Feb;35(2):135-46. doi: 10.1007/s10875-014-0125-1. Epub 2015 Jan 28. PMID: 25627829; PMCID: PMC4352191.

180: Lemaire R, Prasad J, Kashima T, Gustafson J, Manley JL, Lafyatis R. Stability of a PKCI-1-related mRNA is controlled by the splicing factor ASF/SF2: a novel function for SR proteins. Genes Dev. 2002 Mar 1;16(5):594-607. doi: 10.1101/gad.939502. PMID: 11877379; PMCID: PMC155348.

181: Weisbart RH, Chan G, Li E, Farmani N, Heinze E, Rubell A, Nishimura RN, Colburn K. BRAF splice variants in rheumatoid arthritis synovial fibroblasts activate MAPK through CRAF. Mol Immunol. 2013 Oct;55(3-4):247-52. doi: 10.1016/j.molimm.2013.02.001. Epub 2013 Mar 19. PMID: 23517740.

182: Pedretti M, Rancic Z, Soltermann A, Herzog BA, Schliemann C, Lachat M, Neri D, Kaufmann PA. Comparative immunohistochemical staining of atherosclerotic plaques using F16, F8 and L19: Three clinical-grade fully human antibodies. Atherosclerosis. 2010 Feb;208(2):382-9. doi: 10.1016/j.atherosclerosis.2009.07.043. Epub 2009 Jul 30. PMID: 19699478.

183: Cai L, Brophy RH, Tycksen ED, Duan X, Nunley RM, Rai MF. Distinct expression pattern of periostin splice variants in chondrocytes and ligament progenitor cells. FASEB J. 2019 Jul;33(7):8386-8405. doi: 10.1096/fj.201802281R. Epub 2019 Apr 16. PMID: 30991832; PMCID: PMC6593895.

184: Boyle DL, Shi Y, Gay S, Firestein GS. Regulation of CS1 fibronectin expression and function by IL-1 in endothelial cells. Cell Immunol. 2000 Feb 25;200(1):1-7. doi: 10.1006/cimm.2000.1610. PMID: 10716877.

185: Schwager K, Kaspar M, Bootz F, Marcolongo R, Paresce E, Neri D, Trachsel E. Preclinical characterization of DEKAVIL (F8-IL10), a novel clinical-stage immunocytokine which inhibits the progression of collagen-induced arthritis. Arthritis Res Ther. 2009;11(5):R142. doi: 10.1186/ar2814. Epub 2009 Sep 25. PMID: 19781067; PMCID: PMC2787264.

186: Kono M, Kurita T, Yasuda S, Kono M, Fujieda Y, Bohgaki T, Katsuyama T, Tsokos GC, Moulton VR, Atsumi T. Decreased Expression of Serine/Arginine-Rich Splicing Factor 1 in T Cells From Patients With Active Systemic Lupus Erythematosus Accounts for Reduced Expression of RasGRP1 and DNA Methyltransferase 1. Arthritis Rheumatol. 2018 Dec;70(12):2046-2056. doi: 10.1002/art.40585. Epub 2018 Oct 1. PMID: 29905030.

187: AlFadhli S. Overexpression and secretion of the soluble CTLA-4 splice variant in various autoimmune diseases and in cases with overlapping autoimmunity. Genet Test Mol Biomarkers. 2013 Apr;17(4):336-41. doi: 10.1089/gtmb.2012.0391. Epub 2013 Feb 28. PMID: 23448385; PMCID: PMC3609615.

188: Oparina NY, Delgado-Vega AM, Martinez-Bueno M, Magro-Checa C, Fernández C, Castro RO, Pons-Estel BA, D'Alfonso S, Sebastiani GD, Witte T, Lauwerys BR, Endreffy E, Kovács L, Escudero A, López-Pedrera C, Vasconcelos C, da Silva BM, Frostegård J, Truedsson L, Martin J, Raya E, Ortego-Centeno N, de Los Angeles Aguirre M, de Ramón Garrido E, Palma MJ, Alarcon-Riquelme ME, Kozyrev SV. PXK locus in systemic lupus erythematosus: fine mapping and functional analysis reveals novel susceptibility gene ABHD6. Ann Rheum Dis. 2015 Mar;74(3):e14. doi: 10.1136/annrheumdis-2013-204909. Epub 2014 Feb 17. PMID: 24534757.

189: Wei G, Almeida M, Pintacuda G, Coker H, Bowness JS, Ule J, Brockdorff N. Acute depletion of METTL3 implicates <i>N</i>⁶-methyladenosine in alternative intron/exon inclusion in the nascent transcriptome. Genome Res. 2021 Aug;31(8):1395-1408. doi: 10.1101/gr.271635.120. Epub 2021 Jun 15. PMID: 34131006; PMCID: PMC8327914.

190: Thomas H, Beck K, Adamczyk M, Aeschlimann P, Langley M, Oita RC, Thiebach L, Hils M, Aeschlimann D. Transglutaminase 6: a protein associated with central nervous system development and motor function. Amino Acids. 2013 Jan;44(1):161-77. doi: 10.1007/s00726-011-1091-z. Epub 2011 Oct 8. PMID: 21984379; PMCID: PMC3535377.

191: Mamegano K, Kuroki K, Miyashita R, Kusaoi M, Kobayashi S, Matsuta K, Maenaka K, Colonna M, Ozaki S, Hashimoto H, Takasaki Y, Tokunaga K, Tsuchiya N. Association of LILRA2 (ILT1, LIR7) splice site polymorphism with systemic lupus erythematosus and microscopic polyangiitis. Genes Immun. 2008 Apr;9(3):214-23. doi: 10.1038/gene.2008.5. Epub 2008 Feb 14. Erratum in: Genes Immun. 2008 Oct;9(7):650. PMID: 18273033.

192: Terkeltaub R, Lotz M, Johnson K, Deng D, Hashimoto S, Goldring MB, Burton D, Deftos LJ. Parathyroid hormone-related proteins is abundant in osteoarthritic cartilage, and the parathyroid hormone-related protein 1-173 isoform is selectively induced by transforming growth factor beta in articular chondrocytes and suppresses generation of extracellular inorganic pyrophosphate. Arthritis Rheum. 1998 Dec;41(12):2152-64. doi: 10.1002/1529-0131(199812)41:12<2152::AID-ART10>3.0.CO;2-X. PMID: 9870872.

193: Reichenbach G, Starzinski-Powitz A, Doll M, Hrgovic I, Valesky EM, Kippenberger S, Bernd A, Kaufmann R, Meissner M. Ligand activation of peroxisome proliferator-activated receptor delta suppresses cathepsin B expression in human endothelial cells in a posttranslational manner. Exp Dermatol. 2012 Oct;21(10):751-7. doi: 10.1111/exd.12002. PMID: 23078396.

194: Chang HH, Tseng W, Cui J, Costenbader K, Ho IC. Altered expression of protein tyrosine phosphatase, non-receptor type 22 isoforms in systemic lupus erythematosus. Arthritis Res Ther. 2014 Jan 17;16(1):R14. doi: 10.1186/ar4440. PMID: 24433447; PMCID: PMC3979039.

195: Wibulswas A, Croft D, Pitsillides AA, Bacarese-Hamilton I, McIntyre P, Genot E, Kramer IM. Influence of epitopes CD44v3 and CD44v6 in the invasive behavior of fibroblast-like synoviocytes derived from rheumatoid arthritic joints. Arthritis Rheum. 2002 Aug;46(8):2059-64. doi: 10.1002/art.10421. PMID: 12209509.

196: Carrea A, Preisegger MA, Velasco Zamora J, Dewey RA. The mRNA levels of TGF-β Type II receptor splice variants in monocytes are associated with disease activity in patients with rheumatoid arthritis. Clin Exp Rheumatol. 2021 Mar- Apr;39(2):310-318. doi: 10.55563/clinexprheumatol/ck1ksi. Epub 2020 May 20. PMID: 32452354.

197: Raben N, Nichols RC, Martiniuk F, Plotz PH. A model of mRNA splicing in adult lysosomal storage disease (glycogenosis type II). Hum Mol Genet. 1996 Jul;5(7):995-1000. doi: 10.1093/hmg/5.7.995. PMID: 8817337.

198: Peake NJ, Khawaja K, Myers A, Nowell MA, Jones SA, Rowan AD, Cawston TE, Foster HE. Interleukin-6 signalling in juvenile idiopathic arthritis is limited by proteolytically cleaved soluble interleukin-6 receptor. Rheumatology (Oxford). 2006 Dec;45(12):1485-9. doi: 10.1093/rheumatology/kel154. Epub 2006 May 11. PMID: 16690760.

199: Claudepierre P, Allanore Y, Belec L, Larget-Piet B, Zardi L, Chevalier X. Increased Ed-B fibronectin plasma levels in spondyloarthropathies: comparison with rheumatoid arthritis patients

and a healthy population. Rheumatology (Oxford). 1999 Nov;38(11):1099-103. doi: 10.1093/rheumatology/38.11.1099. PMID: 10556262.

200: Diaz-Gallo LM, Martin J. PTPN22 splice forms: a new role in rheumatoid arthritis. Genome Med. 2012 Feb 24;4(2):13. doi: 10.1186/gm312. PMID: 22364193; PMCID: PMC3392759.

201: Bäckdahl L, Ekman D, Jagodic M, Olsson T, Holmdahl R. Identification of candidate risk gene variations by whole-genome sequence analysis of four rat strains commonly used in inflammation research. BMC Genomics. 2014 May 21;15(1):391. doi: 10.1186/1471-2164-15-391. PMID: 24885425; PMCID: PMC4041999.

202: Ala-Kokko L, Prockop DJ. Completion of the intron-exon structure of the gene for human type II procollagen (COL2A1): variations in the nucleotide sequences of the alleles from three chromosomes. Genomics. 1990 Nov;8(3):454-60. doi: 10.1016/0888-7543(90)90031-o. PMID: 1981048.

203: Kojo S, Tsutsumi A, Goto D, Sumida T. Low expression levels of soluble CD1d gene in patients with rheumatoid arthritis. J Rheumatol. 2003 Dec;30(12):2524-8. PMID: 14719188.

204: Fattorusso R, Pellecchia M, Viti F, Neri P, Neri D, Wüthrich K. NMR structure of the human oncofoetal fibronectin ED-B domain, a specific marker for angiogenesis. Structure. 1999 Apr 15;7(4):381-90. doi: 10.1016/s0969-2126(99)80051-3. PMID: 10196121.

205: Valas S, Rolland M, Perrin C, Perrin G, Mamoun RZ. Characterization of a new 5' splice site within the caprine arthritis encephalitis virus genome: evidence for a novel auxiliary protein. Retrovirology. 2008 Feb 29;5:22. doi: 10.1186/1742-4690-5-22. PMID: 18312636; PMCID: PMC2291067.

206: Carsons S. Extra domain-positive fibronectins in arthritis: wolf in sheep's clothing? Rheumatology (Oxford). 2001 Jul;40(7):721-3. doi: 10.1093/rheumatology/40.7.721. PMID: 11477275.

207: Kim IG, McBride OW, Wang M, Kim SY, Idler WW, Steinert PM. Structure and organization of the human transglutaminase 1 gene. J Biol Chem. 1992 Apr 15;267(11):7710-7. PMID: 1348508.

208: Dickie LJ, Aziz AM, Savic S, Lucherini OM, Cantarini L, Geiler J, Wong CH, Coughlan R, Lane T, Lachmann HJ, Hawkins PN, Robinson PA, Emery P, McGonagle D, McDermott MF. Involvement of X-box binding protein 1 and reactive oxygen species pathways in the pathogenesis of tumour necrosis factor receptor-associated periodic syndrome. Ann Rheum Dis. 2012 Dec;71(12):2035-43. doi: 10.1136/annrheumdis-2011-201197. Epub 2012 Jun 7. PMID: 22679299.

209: Del Galdo F, Maul GG, Jiménez SA, Artlett CM. Expression of allograft inflammatory factor 1 in tissues from patients with systemic sclerosis and in vitro differential expression of its isoforms in response to transforming growth factor beta. Arthritis Rheum. 2006 Aug;54(8):2616-25. doi: 10.1002/art.22010. PMID: 16868985.

210: Guo S, Zhu Q, Jiang T, Wang R, Shen Y, Zhu X, Wang Y, Bai F, Ding Q, Zhou X, Chen G, He DY. Genome-wide DNA methylation patterns in CD4+ T cells from Chinese Han patients with rheumatoid arthritis. Mod Rheumatol. 2017 May;27(3):441-447. doi: 10.1080/14397595.2016.1218595. Epub 2016 Sep 1. PMID: 27585642.

211: Faggian J, Fosang AJ, Zieba M, Wallace MJ, Hooper SB. Changes in versican and chondroitin sulfate proteoglycans during structural development of the lung. Am J Physiol Regul Integr Comp Physiol. 2007 Aug;293(2):R784-92. doi: 10.1152/ajpregu.00801.2006. Epub 2007 May 23. PMID: 17522116.

212: Gill RB, Day A, Barstow A, Liu H, Zaman G, Dhoot GK. Sulf2 gene is alternatively spliced in mammalian developing and tumour tissues with functional implications. Biochem Biophys Res Commun. 2011 Oct 28;414(3):468-73. doi: 10.1016/j.bbrc.2011.09.088. Epub 2011 Sep 24. PMID: 21968018.

213: Croft DR, Dall P, Davies D, Jackson DG, McIntyre P, Kramer IM. Complex CD44 splicing combinations in synovial fibroblasts from arthritic joints. Eur J Immunol. 1997 Jul;27(7):1680-4. doi: 10.1002/eji.1830270713. PMID: 9247577.

214: Michel J, Langstein J, Hofstädter F, Schwarz H. A soluble form of CD137 (ILA/4-1BB), a member of the TNF receptor family, is released by activated lymphocytes and is detectable in sera of patients with rheumatoid arthritis. Eur J Immunol. 1998 Jan;28(1):290-5. doi: 10.1002/(SICI)1521-4141(199801)28:01<290::AID-IMMU290>3.0.CO;2-S. PMID: 9485208.

215: Wen F, Ellingson SM, Kyogoku C, Peterson EJ, Gaffney PM. Exon 6 variants carried on systemic lupus erythematosus (SLE) risk haplotypes modulate IRF5 function. Autoimmunity. 2011 Mar;44(2):82-9. doi: 10.3109/08916934.2010.491842. Epub 2010 Aug 9. PMID: 20695768; PMCID: PMC3104271.

216: Li H, Reksten TR, Ice JA, Kelly JA, Adrianto I, Rasmussen A, Wang S, He B, Grundahl KM, Glenn SB, Miceli-Richard C, Bowman S, Lester S, Eriksson P, Eloranta ML, Brun JG, Gøransson LG, Harboe E, Guthridge JM, Kaufman KM, Kvarnström M, Cunninghame Graham DS, Patel K, Adler AJ, Farris AD, Brennan MT, Chodosh J, Gopalakrishnan R, Weisman MH, Venuturupalli S, Wallace DJ, Hefner KS, Houston GD, Huang AJW, Hughes PJ, Lewis DM, Radfar L, Vista ES, Edgar CE, Rohrer MD, Stone DU, Vyse TJ, Harley JB, Gaffney PM, James JA, Turner S, Alevizos I, Anaya JM, Rhodus NL, Segal BM, Montgomery CG, Scofield RH, Kovats S, Mariette X, Rönnblom L, Witte T, Rischmueller M, Wahren-Herlenius M, Omdal R, Jonsson R, Ng WF; for UK Primary Sjögren's Syndrome Registry; Nordmark G, Lessard CJ, Sivils KL. Identification of a Sjögren's syndrome susceptibility locus at OAS1 that influences isoform switching, protein expression, and responsiveness to type I interferons. PLoS Genet. 2017 Jun 22;13(6):e1006820. doi: 10.1371/journal.pgen.1006820. PMID: 28640813; PMCID: PMC5501660.

217: Cutolo M, Picasso M, Ponassi M, Sun MZ, Balza E. Tenascin and fibronectin distribution in human normal and pathological synovium. J Rheumatol. 1992 Sep;19(9):1439-47. PMID: 1279171.

218: Schaaf MJ, Cidlowski JA. AUUUA motifs in the 3'UTR of human glucocorticoid receptor alpha and beta mRNA destabilize mRNA and decrease receptor protein expression. Steroids. 2002 Jun;67(7):627-36. doi: 10.1016/s0039-128x(02)00015-6. PMID: 11996936.

219: Kerna I, Kisand K, Suutre S, Murde M, Tamm A, Kumm J, Tamm A. The ADAM12 is upregulated in synovitis and postinflammatory fibrosis of the synovial membrane in patients with early radiographic osteoarthritis. Joint Bone Spine. 2014 Jan;81(1):51-6. doi: 10.1016/j.jbspin.2013.03.007. Epub 2013 Apr 9. PMID: 23578941.

220: Rogers GR, Markova NG, De Laurenzi V, Rizzo WB, Compton JG. Genomic organization and expression of the human fatty aldehyde dehydrogenase gene (FALDH). Genomics. 1997 Jan 15;39(2):127-35. doi: 10.1006/geno.1996.4501. PMID: 9027499.

221: Tsukada Y, Ichikawa H, Chai Z, Lai FP, Dunster K, Sentry JW, Toh BH. Novel variant of p230 trans-Golgi network protein identified by serum from Sjögren's syndrome patient. Eur J Cell Biol. 2000 Nov;79(11):790-4. doi: 10.1078/0171-9335-00114. PMID: 11139141.

222: Nedvetzki S, Walmsley M, Alpert E, Williams RO, Feldmann M, Naor D. CD44 involvement in experimental collagen-induced arthritis (CIA). J Autoimmun. 1999 Aug;13(1):39-47. doi: 10.1006/jaut.1999.0294. PMID: 10441166.

223: Mokuda S, Miyazaki T, Ito Y, Yamasaki S, Inoue H, Guo Y, Kong WS, Kanno M, Takasugi K, Sugiyama E, Masumoto J. The proto-oncogene survivin splice variant 2B is induced by PDGF and leads to cell proliferation in rheumatoid arthritis fibroblast-like synoviocytes. Sci Rep. 2015 May 22;5:9795. doi: 10.1038/srep09795. PMID: 25997820; PMCID: PMC4441133.

224: Ohkubo T, Takei M, Mitamura K, Horie T, Fujiwara S, Shimizu K, Ryu J, Shiraiwa H, Sawada S. Increased soluble CD4 molecules and the role of soluble CD4 production in patients with rheumatoid arthritis. J Int Med Res. 2001 Nov- Dec;29(6):488-96. doi: 10.1177/147323000102900604. PMID: 11803732.

225: Vollmer S, Vater A, Licha K, Gemeinhardt I, Gemeinhardt O, Voigt J, Ebert B, Schnorr J, Taupitz M, Macdonald R, Schirner M. Extra domain B fibronectin as a target for near-infrared fluorescence imaging of rheumatoid arthritis affected joints in vivo. Mol Imaging. 2009 Dec;8(6):330-40. PMID: 20003891.

226: Hikichi Y, Yoshimura K, Takigawa M. All-trans retinoic acid-induced ADAM28 degrades proteoglycans in human chondrocytes. Biochem Biophys Res Commun. 2009 Aug 21;386(2):294-9. doi: 10.1016/j.bbrc.2009.06.052. Epub 2009 Jun 13. PMID: 19527685.

227: Roescher N, Vosters JL, Alsaleh G, Dreyfus P, Jacques S, Chiocchia G, Sibilia J, Tak PP, Chiorini JA, Mariette X, Gottenberg JE. Targeting the splicing of mRNA in autoimmune diseases: BAFF inhibition in Sjögren's syndrome as a proof of concept. Mol Ther. 2014 Apr;22(4):821-7. doi: 10.1038/mt.2013.275. Epub 2013 Dec 5. PMID: 24304965; PMCID: PMC3982500.

228: Kapsogeorgou EK, Manoussakis MN. Salivary gland epithelial cells (SGEC): carriers of exquisite B7-2 (CD86) costimulatory molecules. J Autoimmun. 2010 Nov;35(3):188-91. doi: 10.1016/j.jaut.2010.06.006. PMID: 20643530.

229: Fay J, Varoga D, Wruck CJ, Kurz B, Goldring MB, Pufe T. Reactive oxygen species induce expression of vascular endothelial growth factor in chondrocytes and human articular cartilage explants. Arthritis Res Ther. 2006;8(6):R189. doi: 10.1186/ar2102. PMID: 17187682; PMCID: PMC1794535.

230: Jiang H, Knudson CB, Knudson W. Antisense inhibition of CD44 tailless splice variant in human articular chondrocytes promotes hyaluronan internalization. Arthritis Rheum. 2001 Nov;44(11):2599-610. doi: 10.1002/1529-0131(200111)44:11<2599::aid-art440>3.0.co;2-y. PMID: 11710716.

231: Tolboom TC, Huidekoper AL, Kramer IM, Pieterman E, Toes RE, Huizinga TW. Correlation between expression of CD44 splice variant v8-v9 and invasiveness of fibroblast-like synoviocytes in an in vitro system. Clin Exp Rheumatol. 2004 Mar-Apr;22(2):158-64. PMID: 15083882.

232: Peters JH, Carsons S, Kalunian K, McDougall S, Yoshida M, Ko F, van der Vliet-Hristova M, Hahn TJ. Preferential recognition of a fragment species of osteoarthritic synovial fluid fibronectin by antibodies to the alternatively spliced EIIIA segment. Arthritis Rheum. 2001 Nov;44(11):2572-85. doi: 10.1002/1529-0131(200111)44:11<2572::aid-art438>3.0.co;2-y. PMID: 11710714.

233: Niarakis A, Giannopoulou E, Ravazoula P, Panagiotopoulos E, Zarkadis IK, Aletras AJ. Detection of a latent soluble form of membrane type 1 matrix metalloprotease bound with tissue inhibitor of matrix metalloproteinases-2 in periprosthetic tissues and fluids from loose arthroplasty endoprostheses. FEBS J. 2013 Dec;280(24):6541-55. doi: 10.1111/febs.12555. Epub 2013 Oct 25. PMID: 24112707.

234: Hirayasu K, Ohashi J, Kashiwase K, Takanashi M, Satake M, Tokunaga K, Yabe T. Long-term persistence of both functional and non-functional alleles at the leukocyte immunoglobulin-like receptor A3 (LILRA3) locus suggests balancing selection. Hum Genet. 2006 May;119(4):436-43. doi: 10.1007/s00439-006-0152-y. Epub 2006 Feb 24. PMID: 16501917.

235: Yousaf N, Low WY, Onipinla A, Mein C, Caulfield M, Munroe PB, Chernajovsky Y. Differences between disease-associated endoplasmic reticulum aminopeptidase 1 (ERAP1) isoforms in cellular expression, interactions with tumour necrosis factor receptor 1 (TNF-R1) and

regulation by cytokines. Clin Exp Immunol. 2015 May;180(2):289-304. doi: 10.1111/cei.12575. PMID: 25545008; PMCID: PMC4408164.

236: Hasegawa M, Nakoshi Y, Muraki M, Sudo A, Kinoshita N, Yoshida T, Uchida A. Expression of large tenascin-C splice variants in synovial fluid of patients with rheumatoid arthritis. J Orthop Res. 2007 May;25(5):563-8. doi: 10.1002/jor.20366. PMID: 17262825.

237: Wainwright SD, Bondeson J, Hughes CE. An alternative spliced transcript of ADAMTS4 is present in human synovium from OA patients. Matrix Biol. 2006 Jul;25(5):317-20. doi: 10.1016/j.matbio.2006.03.006. Epub 2006 Apr 6. PMID: 16723216.

238: Buyon JP, Tseng CE, Di Donato F, Rashbaum W, Morris A, Chan EK. Cardiac expression of 52beta, an alternative transcript of the congenital heart block- associated 52-kd SS-A/Ro autoantigen, is maximal during fetal development. Arthritis Rheum. 1997 Apr;40(4):655-60. doi: 10.1002/art.1780400410. PMID: 9125247.

239: Sciore P, Frank CB, Hart DA. Identification of sex hormone receptors in human and rabbit ligaments of the knee by reverse transcription-polymerase chain reaction: evidence that receptors are present in tissue from both male and female subjects. J Orthop Res. 1998 Sep;16(5):604-10. doi: 10.1002/jor.1100160513. PMID: 9820285.

240: Greetham D, Ellis CD, Mewar D, Fearon U, an Ultaigh SN, Veale DJ, Guesdon F, Wilson AG. Functional characterization of NF-kappaB inhibitor-like protein 1 (NFkappaBIL1), a candidate susceptibility gene for rheumatoid arthritis. Hum Mol Genet. 2007 Dec 15;16(24):3027-36. doi: 10.1093/hmg/ddm261. Epub 2007 Sep 12. PMID: 17855452.

241: Hoornaert KP, Vereecke I, Dewinter C, Rosenberg T, Beemer FA, Leroy JG, Bendix L, Björck E, Bonduelle M, Boute O, Cormier-Daire V, De Die-Smulders C, Dieux-Coeslier A, Dollfus H, Elting M, Green A, Guerci VI, Hennekam RC, Hilhorts-Hofstee Y, Holder M, Hoyng C, Jones KJ, Josifova D, Kaitila I, Kjaergaard S, Kroes YH, Lagerstedt K, Lees M, Lemerrer M, Magnani C, Marcelis C, Martorell L, Mathieu M, McEntagart M, Mendicino A, Morton J, Orazio G, Paquis V, Reish O, Simola KO, Smithson SF, Temple KI, Van Aken E, Van Bever Y, van den Ende J, Van Hagen JM, Zelante L, Zordania R, De Paepe A, Leroy BP, De Buyzere M, Coucke PJ, Mortier GR. Stickler syndrome caused by COL2A1 mutations: genotype- phenotype correlation in a series of 100 patients. Eur J Hum Genet. 2010 Aug;18(8):872-80. doi: 10.1038/ejhg.2010.23. Epub 2010 Feb 24. Erratum in: Eur J Hum Genet. 2010 Aug;18(8):881. PMID: 20179744; PMCID: PMC2987380.

242: Alvarez-Errico D, Yamashita Y, Suzuki R, Odom S, Furumoto Y, Yamashita T, Rivera J. Functional analysis of Lyn kinase A and B isoforms reveals redundant and distinct roles in Fc epsilon RI-dependent mast cell activation. J Immunol. 2010 May 1;184(9):5000-8. doi: 10.4049/jimmunol.0904064. Epub 2010 Mar 22. PMID: 20308635; PMCID: PMC2948211.

243: Kalinski H, Yaniv A, Mashiah P, Miki T, Tronick SR, Gazit A. rev-like transcripts of caprine arthritis encephalitis virus. Virology. 1991 Aug;183(2):786-92. doi: 10.1016/0042-6822(91)91012-6. PMID: 1649509.

244: Derijk RH, Schaaf MJ, Turner G, Datson NA, Vreugdenhil E, Cidlowski J, de Kloet ER, Emery P, Sternberg EM, Detera-Wadleigh SD. A human glucocorticoid receptor gene variant that increases the stability of the glucocorticoid receptor beta-isoform mRNA is associated with rheumatoid arthritis. J Rheumatol. 2001 Nov;28(11):2383-8. PMID: 11708406.

245: Shiozawa K, Hino K, Shiozawa S. Alternatively spliced EDA-containing fibronectin in synovial fluid as a predictor of rheumatoid joint destruction. Rheumatology (Oxford). 2001 Jul;40(7):739-42. doi: 10.1093/rheumatology/40.7.739. PMID: 11477277.

246: Ray BK, Murphy R, Ray P, Ray A. SAF-2, a splice variant of SAF-1, acts as a negative regulator of transcription. J Biol Chem. 2002 Nov 29;277(48):46822-30. doi: 10.1074/jbc.M206299200. Epub 2002 Sep 20. PMID: 12270922.

247: Lemaire R, Flipo RM, Migaud H, Fontaine C, Huet G, Dacquembronne E, Lafyatis R. Alternative splicing of the 5' region of cathepsin B pre-messenger RNA in rheumatoid synovial tissue. Arthritis Rheum. 1997 Aug;40(8):1540-2. doi: 10.1002/art.1780400824. PMID: 9259437.

248: Liu JH, Wei S, Lamy T, Li Y, Epling-Burnette PK, Djeu JY, Loughran TP Jr. Blockade of Fas-dependent apoptosis by soluble Fas in LGL leukemia. Blood. 2002 Aug 15;100(4):1449-53. PMID: 12149230.

249: Dugan J, Griffiths E, Snow P, Rosenzweig H, Lee E, Brown B, Carr DW, Rose C, Rosenbaum J, Davey MP. Blau syndrome-associated Nod2 mutation alters expression of full-length NOD2 and limits responses to muramyl dipeptide in knock-in mice. J Immunol. 2015 Jan 1;194(1):349-57. doi: 10.4049/jimmunol.1402330. Epub 2014 Nov 26. PMID: 25429073; PMCID: PMC4722953.

250: Maretzky T, Le Gall SM, Worpenberg-Pietruk S, Eder J, Overall CM, Huang XY, Poghosyan Z, Edwards DR, Blobel CP. Src stimulates fibroblast growth factor receptor-2 shedding by an ADAM15 splice variant linked to breast cancer. Cancer Res. 2009 Jun 1;69(11):4573-6. doi: 10.1158/0008-5472.CAN-08-4766. PMID: 19487280.

251: Banda NK, Mehta G, Kjaer TR, Takahashi M, Schaack J, Morrison TE, Thiel S, Arend WP, Holers VM. Essential role for the lectin pathway in collagen antibody- induced arthritis revealed through use of adenovirus programming complement inhibitor MAp44 expression. J Immunol. 2014 Sep 1;193(5):2455-68. doi: 10.4049/jimmunol.1400752. Epub 2014 Jul 28. PMID: 25070856; PMCID: PMC4134985.

252: Sarkissian M, Lafyatis R. Transforming growth factor-beta and platelet derived growth factor regulation of fibrillar fibronectin matrix formation by synovial fibroblasts. J Rheumatol. 1998 Apr;25(4):613-22. PMID: 9558159.

253: Labat-Robert J, Chevalier X. Fibronectine, vieillissement et pathologies associées [Fibronectin, aging and related pathologies]. C R Seances Soc Biol Fil. 1991;185(3):121-6. French. PMID: 1835421.

254: Wan B, Nie H, Liu A, Feng G, He D, Xu R, Zhang Q, Dong C, Zhang JZ. Aberrant regulation of synovial T cell activation by soluble costimulatory molecules in rheumatoid arthritis. J Immunol. 2006 Dec 15;177(12):8844-50. doi: 10.4049/jimmunol.177.12.8844. PMID: 17142787.

255: Proussakova OV, Rabaya NA, Moshnikova AB, Telegina ES, Turanov A, Nanazashvili MG, Beletsky IP. Oligomerization of soluble Fas antigen induces its cytotoxicity. J Biol Chem. 2003 Sep 19;278(38):36236-41. doi: 10.1074/jbc.M305896200. Epub 2003 Jul 10. Erratum in: J Biol Chem. 2004 Feb 20;279(8):7359. PMID: 12855687.

256: Pufe T, Petersen W, Tillmann B, Mentlein R. The splice variants VEGF121 and VEGF189 of the angiogenic peptide vascular endothelial growth factor are expressed in osteoarthritic cartilage. Arthritis Rheum. 2001 May;44(5):1082-8. doi: 10.1002/1529-0131(200105)44:5<1082::AID-ANR188>3.0.CO;2-X. PMID: 11352239.

257: Wainwright SD, Bondeson J, Caterson B, Hughes CE. ADAMTS-4_v1 is a splice variant of ADAMTS-4 that is expressed as a protein in human synovium and cleaves aggrecan at the interglobular domain. Arthritis Rheum. 2013 Nov;65(11):2866-75. doi: 10.1002/art.38102. PMID: 23897278; PMCID: PMC4312973.

258: Banda NK, Desai D, Scheinman RI, Pihl R, Sekine H, Fujita T, Sharma V, Hansen AG, Garred P, Thiel S, Borodovsky A, Holers VM. Targeting of Liver Mannan-Binding Lectin-Associated Serine Protease-3 with RNA Interference Ameliorates Disease in a Mouse Model of Rheumatoid Arthritis. Immunohorizons. 2018 Sep;2(8):274-295. doi: 10.4049/immunohorizons.1800053. PMID: 30417171; PMCID: PMC6220895.

259: Oda H, Beck DB, Kuehn HS, Sampaio Moura N, Hoffmann P, Ibarra M, Stoddard J, Tsai WL, Gutierrez-Cruz G, Gadina M, Rosenzweig SD, Kastner DL, Notarangelo LD, Aksentijevich I. Second Case of HOIP Deficiency Expands Clinical Features and Defines Inflammatory Transcriptome Regulated by LUBAC. Front Immunol. 2019 Mar 18;10:479. doi: 10.3389/fimmu.2019.00479. PMID: 30936877; PMCID: PMC6431612.

260: Ryder LR, Ryder LP, Bartels EM, Woetmann A, Madsen HO, Ødum N, Danneskiold- Samsøe B, Ribel-Madsen S, Bliddal H. Differential effects of decoy receptor- and antibody-mediated tumour necrosis factor blockage on FoxP3 expression in responsive arthritis patients. APMIS. 2013 Apr;121(4):337-47. doi: 10.1111/apm.12004. Epub 2012 Sep 23. PMID: 23031059.

261: Claus R, Bittorf T, Walzel H, Brock J, Uhde R, Meiske D, Schulz U, Hobusch D, Schumacher K, Witt M, Bartel F, Hausmann S. High concentration of soluble HLA-DR in the synovial fluid: generation and significance in "rheumatoid-like" inflammatory joint diseases. Cell

Immunol. 2000 Dec 15;206(2):85-100. doi: 10.1006/cimm.2000.1729. PMID: 11161440.

262: Weissbach L, Tran K, Colquhoun SA, Champliaud MF, Towle CA. Detection of an interleukin-1 intracellular receptor antagonist mRNA variant. Biochem Biophys Res Commun. 1998 Mar 6;244(1):91-5. doi: 10.1006/bbrc.1998.8217. PMID: 9514884.

263: Seperack PK, Mercer JA, Strobel MC, Copeland NG, Jenkins NA. Retroviral sequences located within an intron of the dilute gene alter dilute expression in a tissue-specific manner. EMBO J. 1995 May 15;14(10):2326-32. doi: 10.1002/j.1460-2075.1995.tb07227.x. PMID: 7774591; PMCID: PMC398341.

264: Andersson SE, Svensson MN, Erlandsson MC, Dehlin M, Andersson KM, Bokarewa MI. Activation of Fms-like tyrosine kinase 3 signaling enhances survivin expression in a mouse model of rheumatoid arthritis. PLoS One. 2012;7(10):e47668. doi: 10.1371/journal.pone.0047668. Epub 2012 Oct 17. PMID: 23082191; PMCID: PMC3474718.

265: Richards AJ, Laidlaw M, Meredith SP, Shankar P, Poulson AV, Scott JD, Snead MP. Missense and silent mutations in COL2A1 result in Stickler syndrome but via different molecular mechanisms. Hum Mutat. 2007 Jun;28(6):639. doi: 10.1002/humu.9497. PMID: 17437277.

266: Woolard J, Wang WY, Bevan HS, Qiu Y, Morbidelli L, Pritchard-Jones RO, Cui TG, Sugiono M, Waine E, Perrin R, Foster R, Digby-Bell J, Shields JD, Whittles CE, Mushens RE, Gillatt DA, Ziche M, Harper SJ, Bates DO. VEGF165b, an inhibitory vascular endothelial growth factor splice variant: mechanism of action, in vivo effect on angiogenesis and endogenous protein expression. Cancer Res. 2004 Nov 1;64(21):7822-35. doi: 10.1158/0008-5472.CAN-04-0934. PMID: 15520188.

267: Kaur G, Goodall JC, Jarvis LB, Hill Gaston JS. Characterisation of Foxp3 splice variants in human CD4+ and CD8+ T cells--identification of Foxp3Δ7 in human regulatory T cells. Mol Immunol. 2010 Nov-Dec;48(1-3):321-32. doi: 10.1016/j.molimm.2010.07.008. Epub 2010 Aug 5. PMID: 20688398.

268: Wibulswas A, Croft D, Bacarese-Hamilton I, McIntyre P, Genot E, Kramer IM. The CD44v7/8 epitope as a target to restrain proliferation of fibroblast-like synoviocytes in rheumatoid arthritis. Am J Pathol. 2000 Dec;157(6):2037-44. doi: 10.1016/S0002-9440(10)64842-0. PMID: 11106576; PMCID: PMC1885774.

269: Nambiar MP, Enyedy EJ, Fisher CU, Krishnan S, Warke VG, Gilliland WR, Oglesby RJ, Tsokos GC. Abnormal expression of various molecular forms and distribution of T cell receptor zeta chain in patients with systemic lupus erythematosus. Arthritis Rheum. 2002 Jan;46(1):163-74. doi: 10.1002/1529-0131(200201)46:1<163::AID-ART10065>3.0.CO;2-J. PMID: 11817588.

270: Aksentijevich I, Galon J, Soares M, Mansfield E, Hull K, Oh HH, Goldbach- Mansky R, Dean J, Athreya B, Reginato AJ, Henrickson M, Pons-Estel B, O'Shea JJ, Kastner DL. The tumor-necrosis-factor receptor-

associated periodic syndrome: new mutations in TNFRSF1A, ancestral origins, genotype-phenotype studies, and evidence for further genetic heterogeneity of periodic fevers. Am J Hum Genet. 2001 Aug;69(2):301-14. doi: 10.1086/321976. Epub 2001 Jul 6. Erratum in: Am J Hum Genet 2001 Nov;69(5):1160. PMID: 11443543; PMCID: PMC1235304.

271: Miyashita K, Sakashita E, Miyamoto K, Tokita M, Komai T. Development of the selective adsorbent for EDA containing fibronectin using heparin immobilized cellulose. Int J Biol Macromol. 1998 Apr;22(2):91-5. doi: 10.1016/s0141-8130(97)00093-7. PMID: 9585886.

272: Elices MJ, Tsai V, Strahl D, Goel AS, Tollefson V, Arrhenius T, Wayner EA, Gaeta FC, Fikes JD, Firestein GS. Expression and functional significance of alternatively spliced CS1 fibronectin in rheumatoid arthritis microvasculature. J Clin Invest. 1994 Jan;93(1):405-16. doi: 10.1172/JCI116975. PMID: 8282813; PMCID: PMC293796.

273: Pufe T, Petersen W, Tillmann B, Mentlein R. Splice variants VEGF121 and VEGF165 of the angiogenic peptide vascular endothelial cell growth factor are expressed in the synovial tissue of patients with rheumatoid arthritis. J Rheumatol. 2001 Jul;28(7):1482-5. PMID: 11469450.

274: Sherman JB, Raben N, Nicastri C, Argov Z, Nakajima H, Adams EM, Eng CM, Cowan TM, Plotz PH. Common mutations in the phosphofructokinase-M gene in Ashkenazi Jewish patients with glycogenesis VII--and their population frequency. Am J Hum Genet. 1994 Aug;55(2):305-13. PMID: 8037209; PMCID: PMC1918380.

275: Del Galdo F, Jiménez SA. T cells expressing allograft inflammatory factor 1 display increased chemotaxis and induce a profibrotic phenotype in normal fibroblasts in vitro. Arthritis Rheum. 2007 Oct;56(10):3478-88. doi: 10.1002/art.22877. PMID: 17907195.

276: Horie R, Ito K, Tatewaki M, Nagai M, Aizawa S, Higashihara M, Ishida T, Inoue J, Takizawa H, Watanabe T. A variant CD30 protein lacking extracellular and transmembrane domains is induced in HL-60 by tetradecanoylphorbol acetate and is expressed in alveolar macrophages. Blood. 1996 Oct 1;88(7):2422-32. PMID: 8839832.

277: Diaz A, Hu C, Kastner DL, Schaner P, Reginato AM, Richards N, Gumucio DL. Lipopolysaccharide-induced expression of multiple alternatively spliced MEFV transcripts in human synovial fibroblasts: a prominent splice isoform lacks the C-terminal domain that is highly mutated in familial Mediterranean fever. Arthritis Rheum. 2004 Nov;50(11):3679-89. doi: 10.1002/art.20600. PMID: 15529356.

278: Chan EK, Di Donato F, Hamel JC, Tseng CE, Buyon JP. 52-kD SS-A/Ro: genomic structure and identification of an alternatively spliced transcript encoding a novel leucine zipper-minus autoantigen expressed in fetal and adult heart. J Exp Med. 1995 Oct 1;182(4):983-92. doi: 10.1084/jem.182.4.983. PMID: 7561701; PMCID: PMC2192297.

279: Kozyrev SV, Lewén S, Reddy PM, Pons-Estel B; Argentine Collaborative Group; Witte T; German Collaborative Group; Junker P,

Laustrup H, Gutiérrez C, Suárez A, Francisca González-Escribano M, Martín J; Spanish Collaborative Group; Alarcón-Riquelme ME. Structural insertion/deletion variation in IRF5 is associated with a risk haplotype and defines the precise IRF5 isoforms expressed in systemic lupus erythematosus. Arthritis Rheum. 2007 Apr;56(4):1234-41. doi: 10.1002/art.22497. PMID: 17393452.

280: Hospach T, Lohse P, Heilbronner H, Dannecker GE, Lohse P. Pseudodominant inheritance of the hyperimmunoglobulinemia D with periodic fever syndrome in a mother and her two monozygotic twins. Arthritis Rheum. 2005 Nov;52(11):3606-10. doi: 10.1002/art.21381. PMID: 16255052.

281: Raben N, Sherman J, Miller F, Mena H, Plotz P. A 5' splice junction mutation leading to exon deletion in an Ashkenazic Jewish family with phosphofructokinase deficiency (Tarui disease). J Biol Chem. 1993 Mar 5;268(7):4963-7. PMID: 8444874.

282: Jackson JR, Minton JA, Ho ML, Wei N, Winkler JD. Expression of vascular endothelial growth factor in synovial fibroblasts is induced by hypoxia and interleukin 1beta. J Rheumatol. 1997 Jul;24(7):1253-9. PMID: 9228120.

283: Bär KJ, Natura G, Telleria-Diaz A, Teschner P, Vogel R, Vasquez E, Schaibl HG, Ebersberger A. Changes in the effect of spinal prostaglandin E2 during inflammation: prostaglandin E (EP1-EP4) receptors in spinal nociceptive processing of input from the normal or inflamed knee joint. J Neurosci. 2004 Jan 21;24(3):642-51. doi: 10.1523/JNEUROSCI.0882-03.2004. PMID: 14736850; PMCID: PMC6729260.

284: Tröster H, Metzger TE, Semsei I, Schwemmle M, Winterpacht A, Zabel B, Bachmann M. One gene, two transcripts: isolation of an alternative transcript encoding for the autoantigen La/SS-B from a cDNA library of a patient with primary Sjögrens' syndrome. J Exp Med. 1994 Dec 1;180(6):2059-67. doi: 10.1084/jem.180.6.2059. PMID: 7964483; PMCID: PMC2191769.

285: Hu SI, Klein M, Carozza M, Rediske J, Peppard J, Qi JS. Identification of a splice variant of neutrophil collagenase (MMP-8). FEBS Lett. 1999 Jan22;443(1):8-10. doi: 10.1016/s0014-5793(98)01654-8. PMID: 9928942.

286: Hino K, Maeda T, Sekiguchi K, Shiozawa K, Hirano H, Sakashita E, Shiozawa S. Adherence of synovial cells on EDA-containing fibronectin. Arthritis Rheum. 1996 Oct;39(10):1685-92. doi: 10.1002/art.1780391011. PMID: 8843859.

287: Shevchenko YO, Compton JG, Toro JR, DiGiovanna JJ, Bale SJ. Splice-site mutation in TGM1 in congenital recessive ichthyosis in American families: molecular, genetic, genealogic, and clinical studies. Hum Genet. 2000 May;106(5):492-9. doi: 10.1007/s004390000284. PMID: 10914678.

288: Hübener C, Mincheva A, Lichter P, Schraven B, Bruyns E. Genomic organization and chromosomal localization of the human gene encoding

the T-cell receptor-interacting molecule (TRIM). Immunogenetics. 2000 Feb;51(2):154-8. doi: 10.1007/s002510050024. PMID: 10663578.

289: Møller HJ, Ingemann-Hansen T, Poulsen JH. The epidermal growth factor-like domain of the human cartilage large aggregating proteoglycan, aggrecan: increased serum concentration in rheumatoid arthritis. Br J Rheumatol. 1994 Jan;33(1):44-7. doi: 10.1093/rheumatology/33.1.44. PMID: 8162456.

290: Kaufman KM, Kirby MY, McClain MT, Harley JB, James JA. Lupus autoantibodies recognize the product of an alternative open reading frame of SmB/B'. Biochem Biophys Res Commun. 2001 Aug 3;285(5):1206-12. doi: 10.1006/bbrc.2001.5302. PMID: 11478783.

291: Chae JJ, Komarow HD, Cheng J, Wood G, Raben N, Liu PP, Kastner DL. Targeted disruption of pyrin, the FMF protein, causes heightened sensitivity to endotoxin and a defect in macrophage apoptosis. Mol Cell. 2003 Mar;11(3):591-604. doi:10.1016/s1097-2765(03)00056-x. PMID: 12667444.

292: Feyertag J, Haberhauer G, Skoumal M, Kittl EM, Bauer K, Dunky A. Serumspiegel löslicher CD44-Isoform-Variante 5 von Patienten mit seropositiver Rheumatoid-Arthritis unter Cyclosporin-A-Therapie [Serum soluble CD44 isoform variant 5 level in patients with seropositive rheumatoid arthritis treated with cyclosporin A]. Acta Med Austriaca. 2000;27(5):156-9. German. PMID: 11261266.

293: Zhang H, Phang D, Laxer RM, Silverman ED, Pan S, Doherty PJ. Evolution of the T cell receptor beta repertoire from synovial fluid T cells of patients with juvenile onset rheumatoid arthritis. J Rheumatol. 1997 Jul;24(7):1396-402. PMID: 9228144.

294: Cole WG. Abnormal skeletal growth in Kniest dysplasia caused by type II collagen mutations. Clin Orthop Relat Res. 1997 Aug;(341):162-9. PMID: 9269170.

295: Gazit A, Mashiah P, Kalinski H, Gast A, Rosin-Abersfeld R, Tronick SR, Yaniv A. Two species of Rev proteins, with distinct N termini, are expressed by caprine arthritis encephalitis virus. J Virol. 1996 Apr;70(4):2674-7. doi: 10.1128/JVI.70.4.2674-2677.1996. PMID: 8642706; PMCID: PMC190122.

296: Nichols RC, Rudolphi O, Ek B, Exelbert R, Plotz PH, Raben N. Glycogenosis type VII (Tarui disease) in a Swedish family: two novel mutations in muscle phosphofructokinase gene (PFK-M) resulting in intron retentions. Am J Hum Genet. 1996 Jul;59(1):59-65. PMID: 8659544; PMCID: PMC1915105.

297: Hiraoka M, Saito I, Tsubota K, Sugai S, Miyasaka N. Augmented expression of CD44 splice variants in lymphoproliferative disorder of the lacrimal gland in Sjögren's syndrome. Jpn J Ophthalmol. 1997 Sep-Oct;41(5):312-8. doi:10.1016/s0021-5155(97)00072-5. PMID: 9363560.

298: Müller-Ladner U, Kriegsmann J, Strahl D, Gay RE, Elices M, Gay S. Messenger-RNA-Expression der alternativ gesplicten CS-1 Fibronektin-

Isoformen im Rheumatoid-Arthritis (RA)-Synovium [Messenger RNA expression of alternatively spliced CS-1 fibronectin isoforms in Rheumatoid arthritis (RA) synovium]. VerhDtsch Ges Pathol. 1996;80:329. German. PMID: 9065044.

299: Li C, Wei P, Wang L, Wang Q, Wang H, Zhang Y. Integrated Analysis of Transcriptome Changes in Osteoarthritis: Gene Expression, Pathways and Alternative Splicing. Cartilage. 2023 Jun;14(2):235-246. doi: 10.1177/19476035231154511. Epub 2023 Feb 17. PMID: 36799242.

300: Muller IB, Lin M, Jonge R, Will N, López-Navarro B, Laken CV, Struys EA, Oudejans CBM, Assaraf YG, Cloos J, Puig-Kröger A, Jansen G. Methotrexate Provokes Disparate Folate Metabolism Gene Expression and Alternative Splicing in Ex Vivo Monocytes and GM-CSF- and M-CSF-Polarized Macrophages. Int J Mol Sci. 2023 Jun 1;24(11):9641. doi: 10.3390/ijms24119641. PMID: 37298590; PMCID: PMC10253671.
